# Solvent- and transition metal-free amide synthesis from phenyl esters and aryl amines†

**DOI:** 10.1039/c8ra10040c

**Published:** 2019-01-11

**Authors:** Sergey A. Rzhevskiy, Alexandra A. Ageshina, Gleb A. Chesnokov, Pavel S. Gribanov, Maxim A. Topchiy, Mikhail S. Nechaev, Andrey F. Asachenko

**Affiliations:** A.V. Topchiev Institute of Petrochemical Synthesis, Russian Academy of Sciences Leninsky Prospect 29 Moscow 119991 Russia aasachenko@ips.ac.ru; M.V. Lomonosov Moscow State University Leninskie Gory 1 (3) Moscow 119991 Russia m.s.nechaev@org.chem.msu.ru; A.N. Nesmeyanov Institute of Organoelement Compounds, Russian Academy of Sciences Vavilov str. 28 Moscow 119991 Russian Federation

## Abstract

A general, economical, and environmentally friendly method of amide synthesis from phenyl esters and aryl amines was developed. This new method has significant advantages compared to previously reported palladium-catalyzed approaches. The reaction is performed transition metal- and solvent-free, using a cheap and environmentally benign base, NaH. This approach enabled us to obtain target amides in high yields with high atom economy.

## Abstract 2

A new environmentally friendly approach to transition metal- and solvent-free amide synthesis from aryl esters and aryl amines is described.

## Graphical abstract

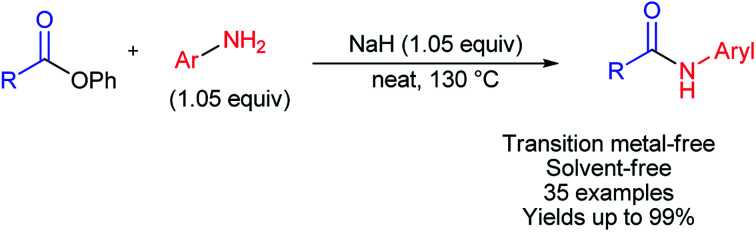

## Introduction

Amides are one of the widest classes of compounds found in natural products, as well as in pharmaceuticals. Their synthesis has been, and continues to be, the focus of significant attention in synthetic chemistry.^1–3^ Many of the well-established methods for amide synthesis involve reagents that are difficult to handle and lead to generation of large quantities of waste products. Recent publications demonstrated the increasing interest of the pharmaceutical industry (*e.g.* ACS Green Chemistry Institute Pharmaceutical Roundtable) in amide bond formation. Thus, amide bond formation is among the most important synthetic transformations requiring improved methods.^4^

Most popular approaches of amide synthesis utilize preliminary preparation of expensive activated esters or use of stoichiometric quantities of peptide-coupling reagents, followed by treatment with amines.^2,5,6^ Several methods of one-pot direct synthesis of amides from carboxylic acids as well as a number of non-conventional approaches, such as oxidative amidation of alcohols, are reported.^3,4,7–9^ Transamidation route to amides is also well-known.^4,10–15^ A number of reviews were published on the methods of amide synthesis.^9,16–19^

Amide synthesis from amines and cheap unactivated esters using various catalysts is rather promising.^20–26^ Recent publications have documented rapid transformation of Buchwald–Hartwig cross-coupling into an efficient tool to create C–N bonds from aryl halides and amines used abundantly in industrial fine organic synthesis.^27–31^

**Scheme 1:**
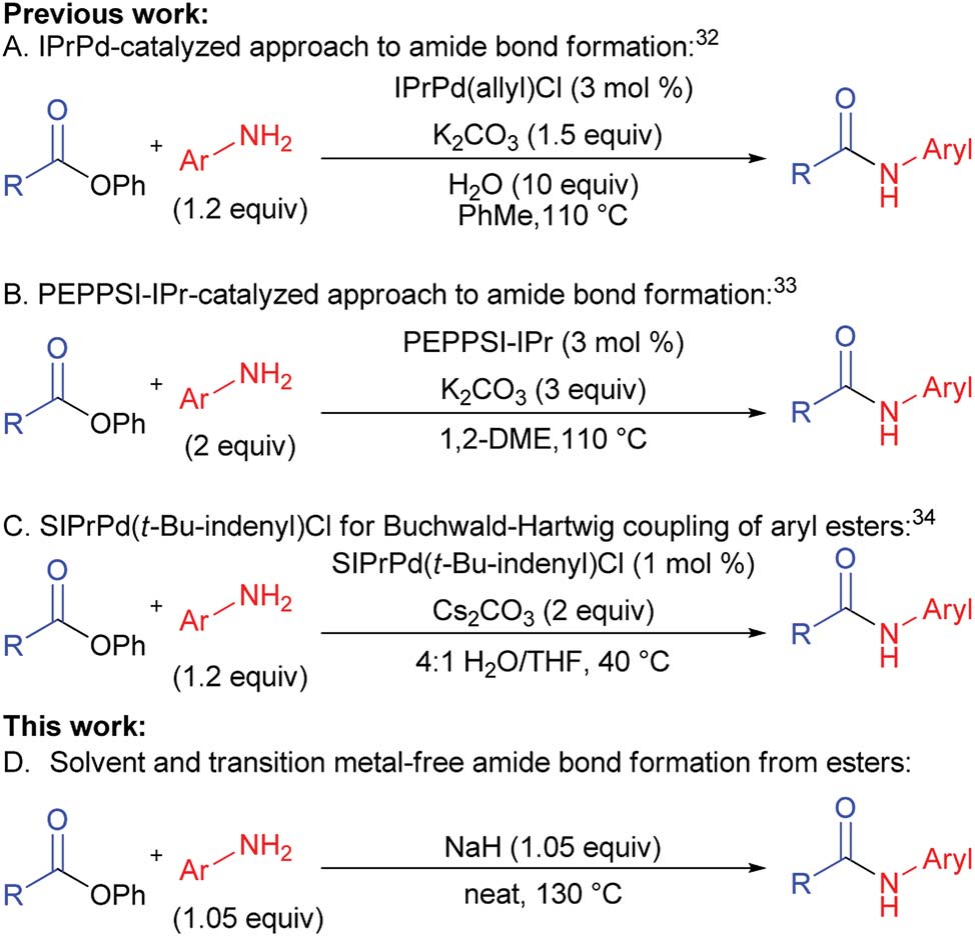
Cross-coupling reactions of amines and esters.

In the past two years, efforts of groups lead by Stephen G. Newman (Scheme 1A),^32^ Michal Szostak (Scheme 1B),^33^ and Nilay Hazari (Scheme 1C)^34^ resulted in a successful transfer of aryl halide cross-coupling techniques onto esters.^35–37^ Chemoselective cleavage of the C(acyl)–O bond provided easy access to various arylamides hardly available by traditional methods.^19,38–40^ These new cross-coupling methods utilize easily available unactivated esters, non-nucleophilic amines, and air-stable catalytic systems. Despite the fact that the above-mentioned methods are rather efficient, they are not free from drawbacks, requiring toxic solvents, transition metal-based catalysts, and generating a lot of waste (low atom economy).

One of the major challenges in organic chemistry is the development of methods that are of high performance, ecologically benign, and economically feasible. Application of solvents as reaction media negatively affects product cost through solvent price and cost of solvent processing or utilization. Besides, usage of solvents can cause harm for employees and environment. Therefore, development of cross-coupling reaction conditions requiring no use of solvents is rather promising since it could lower direct and indirect expenses by means of lower amount of waste, increase of reaction rate, lower catalyst load, and better synthesis scaling up.

For the last several years our group was active in the development of “green” chemical approaches that might be relevant both for academia and industry. Our target is “to eliminate organic solvents from organic chemistry” by development of solvent-free synthetic approaches. This motivated us to report for the first time a new general method of amides synthesis from phenyl esters under green solvent- and transition metal-free conditions. The implementation of this approach promises simplicity, high efficiency, atom economy and ecological safety, while giving opportunities to avoid particular disadvantages of conventional methods (Scheme 1D*vs.*Scheme 1A–C).

## Results and discussion

We performed optimization of solvent-free amidation of phenyl esters using model reaction of *o*-toluidine and phenyl benzoate (Table 1). Initially, we compared performance of IPrPd(allyl)Cl, proposed by Stephen G. Newman, under solvent (Table 1, example 1),^32^ and solvent-free conditions (Table 1, example 2). Yield of product under solvent-free conditions was found to decrease from 98 to 90%. Performing reaction in the absence of both solvent and catalyst resulted in product yield of 32% (Table 1, example 3). In the absence of the catalyst, solvent, and base the reaction proceeded with only 3% yield (Table 1, example 3). Temperature increase up to 150 °C in the absence of base and catalyst have led to a considerable increase of amide yield from 3 to 32% (Table 1, example 4). Utilization of K_2_CO_3_ base under these conditions resulted in a small increase of the product yield compared to base-free conditions, from 32 to 39% (Table 1, example 4). Since further increase of temperature seemed unreasonable, we screened bases available at 150 °C. Replacement of K_2_CO_3_ with Cs_2_CO_3_, decreased the yield down to 15% (Table 1, example 6). Utilization of a strong base *t*-BuOK did not lead to increase of the reaction yield (Table 1, example 7). Reaction proceeded with higher yield (57%) in case of K_3_PO_4_ (Table 1, example 5). Strong organic bases, such as DBN (1,5-diazabicyclo(4.3.0)non-5-ene)^41^ and DABCO (1,4-diazabicyclo[2.2.2]octane) showed moderate yields, whereas DBU (1,8-diazabicyclo[5.4.0]undec-7-ene)^17,18,23^ was slightly more active (Table 1, examples 8–10). The highest yield (80%) was obtained when NaH was used as a base (Table 1, example 11).

**Table 1:** Optimization of the reaction conditions^a^

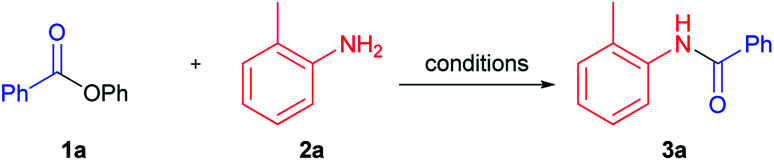				
Entry	Catalyst	Base	*T* (°C)	Yield^b^, %
1	3 mol% IPrPd(allyl)Cl	K_2_CO_3_^c^^,^^d^	110	98
2	3 mol% IPrPd(allyl)Cl	K_2_CO_3_^c^	110	90
3	—	K_2_CO_3_	110	32(3)^e^
4	—	K_2_CO_3_	150	39(32)^e^
5	—	K_3_PO_4_	150	57
6	—	Cs_2_CO_3_	150	15
7	—	*t*-BuOK	150	38
8	—	DBU	150	75
9	—	DBN	150	56
10	—	DABCO	150	61
11	—	NaH	150	80
12	—	NaH	130	97^a^
13	—	NaH	120	95
14	—	NaH	90	80

a Reaction conditions: phenyl benzoate 1a (0.7 mmol), *o*-toluidine 2a (0.735 mmol, 1.05 equiv.), base (0.735 mmol, 1.05 equiv.), *T* °C (oil-bath temperature), 20 h, heat.

b Yield determined by ^1^H NMR of the crude mixture with BHT as internal standard.

c 1.5 equiv. of base.

d Conditions ref. 32.

e Without base.

Next, we studied the temperature effect on the product yield employing NaH as the most suitable base (Table 1, examples 11–14). It turned out that practically quantitative yield was achieved at 130 °C (Table 1, example 12). Thus, heating of nearly equimolar mixture of phenyl benzoate, *ortho*-toluidine and sodium hydride in absence of solvent and palladium catalyst produced target amide 3a in almost quantitative yield.

With optimal conditions in hand, the scope and limitations of the elaborated conditions was examined; we screened various aryl amines in reaction with phenyl benzoate (Table 2). For example, aniline yielded 82% of corresponding amide (Table 2, 3d). The described conditions were found to tolerate halogen-substituted anilines. In case of *meta*- and *para*-substituted F-, Cl-, and Br-substituted anilines, as well as 2-fluoroaniline, amides were obtained in good to quantitative yields (Table 2, 3j, 3k, 3l, 3v, 3w, 3u).

**Table 2:** Scope of anilides^a^^,^^b^

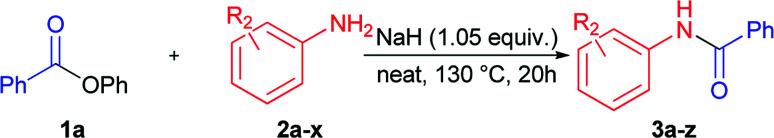
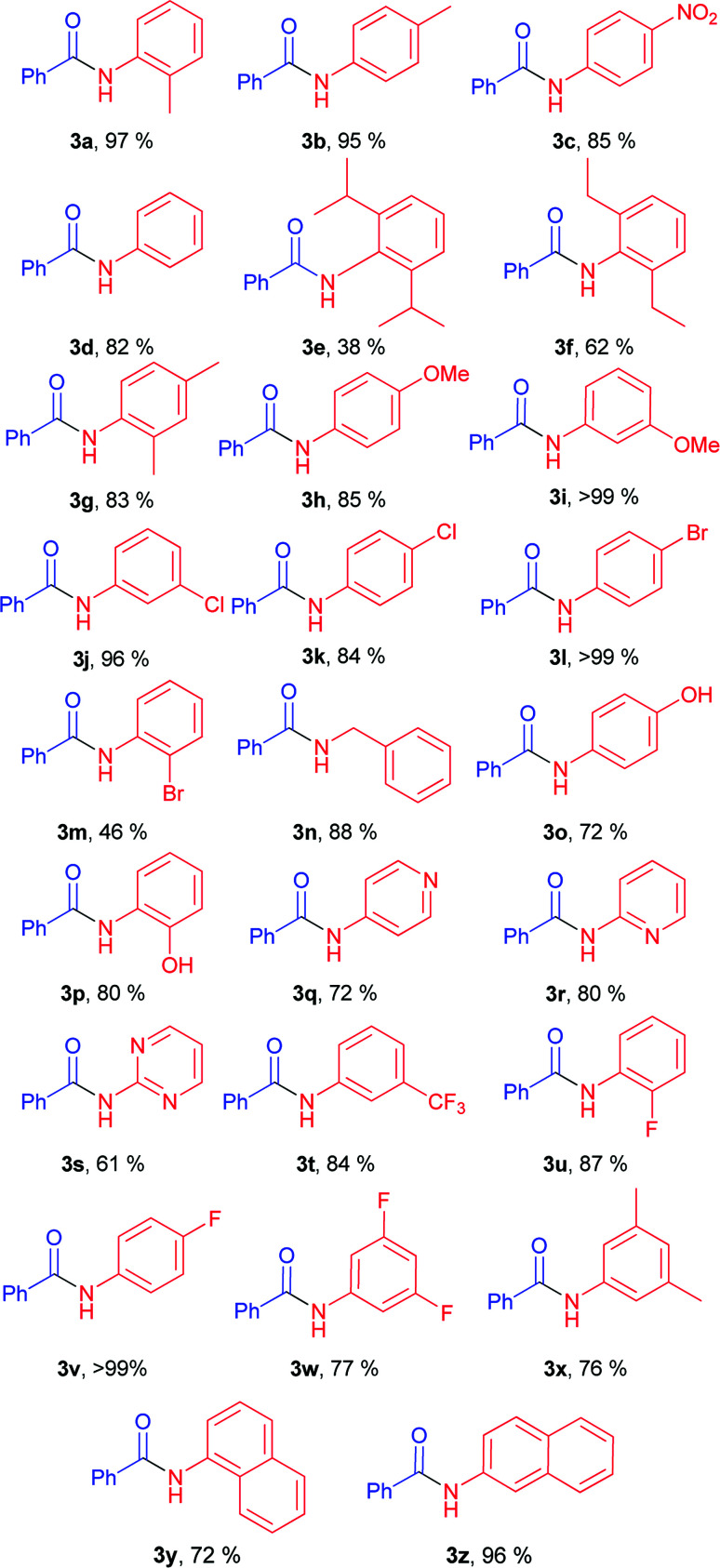

a Reaction conditions: phenyl benzoate 1a (0.7 mmol), amine 2 (0.735 mmol), NaH (0.735 mmol), 130 °C (oil-bath temperature), 20 h.

b Yield determined by ^1^H NMR of the crude mixture with BHT as internal standard.

Anilines bearing one substituent at the *ortho*-position afforded products 3a, 3g, 3p, 3u in high yields. Good yields were obtained for anilines with both donor (Table 2, 3h, 3i, 3o, 3p) and acceptor functional groups (Table 2, 3c, 3t). Utilization of acceptor heterocyclic amines resulted in some decrease in yields (Table 2, 3q, 3r, 3s). Notably, benzylamine also gave good product yield (Table 2, 3n).

Diminished yields were observed in case of anilines bearing bulky substituents in *ortho*-positions, *e.g.* anilines 2e and 2f (38% for 3e, 62% for 3f).

Next stage of our studies was screening of aryl esters in a model reaction with aniline (Table 3).

**Table 3:** Scope of phenyl esters^a^^,^^b^

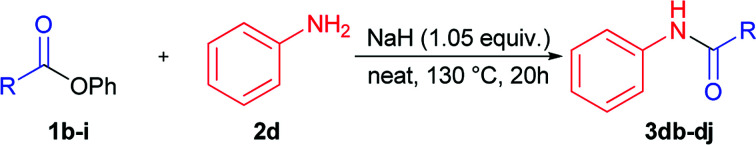
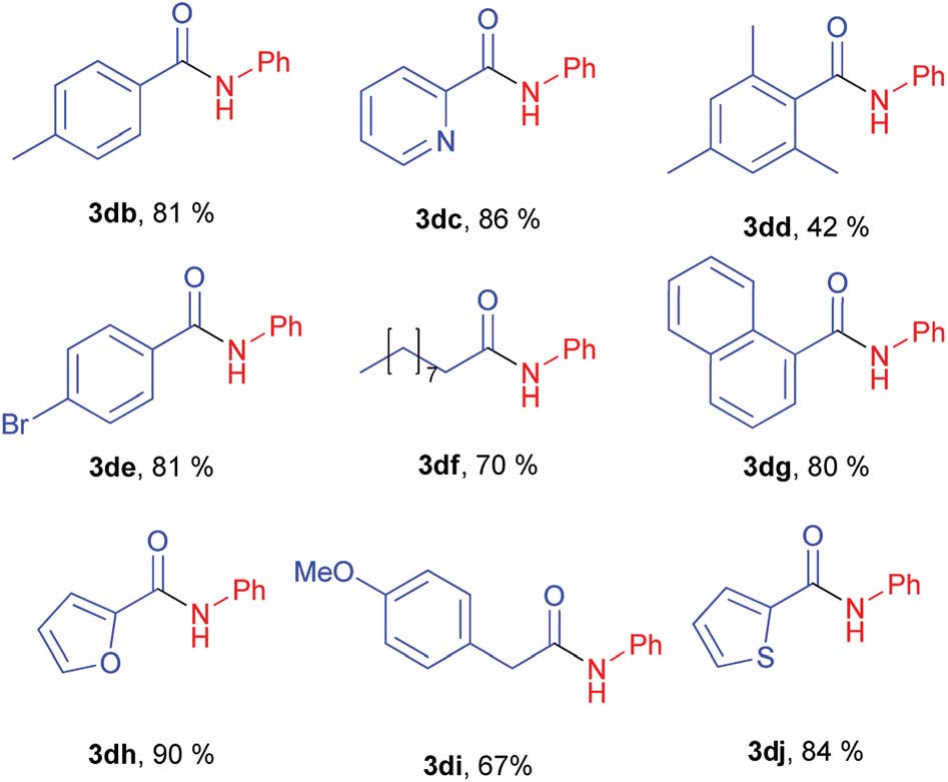

a Reaction conditions: aryl ester 1 (0.7 mmol), aniline 2d (0.735 mmol), NaH (0.735 mmol), 130 °C (oil-bath temperature), 20 h.

b Yield determined by ^1^H NMR of the crude mixture with BHT as internal standard.

For example, esters of aliphatic acids demonstrated lower reactivity compared to phenyl benzoate (Table 3, examples 3df, 3di*vs.*3a of Table 2). The aniline reaction with heterocyclic acids esters proceeded with good yields (Table 3, examples 3dc, 3dh, 3dj).

Interestingly, five-membered heterocyclic esters exhibited higher activity than six-membered counterparts (Table 3, 3dh, 3dj*vs.*3dc). The phenyl esters of *para*-substituted benzoic acids produced amides in high yields (3db, 3de, Table 3*vs.*3d, Table 2). Phenyl ester of sterically hindered 2,4,6-trimethylbenzoic acid showed lower reactivity (Table 3, 3dd).

Thus, we successfully elaborated conditions for high-yield solvent- and transition metal based catalyst-free amide synthesis and tested them on a wide range of substrates.

It should be noted that our method showed product yields comparable to those in palladium-catalyzed methods of amide synthesis (Table 4). In some cases, proposed method showed higher efficiency compared to palladium-catalyzed methods (Table 4, examples 3b, 3o, 3z, 3dh, 3v, 3c).

**Table 4:** Comparison of different methods

Entry	Product	Method A^a^, %	Method B^b^, %	Method C^c^, %	This work^d^, %
1	3a	98	75	96	97
2	3b	—	—	92	95
3	3c	55	—	—	85
4	3d	91	96	90	82
5	3e	—	90	—	38
6	3h	—	84	95	85
7	3o	61	—	—	72
8	3p	93	—	—	80
9	3q	85	—	—	72
10	3v	—	—	95	99
11	3y	—	—	87	72
12	3z	—	—	82	96
13	3dh	68	75	—	90
14	3df	—	78	—	70

a Reaction conditions: 1 (0.2 mmol), 2 (0.24 mmol), IPrPd(allyl)Cl (0.006 mmol), K_2_CO_3_ (0.3 mmol), H_2_O (2 mmol), toluene (1 mL) at 110 °C for 16 h under Ar.

b Reaction conditions: 1 (1.0 equiv.), 2 (2.0 equiv.), K_2_CO_3_ (3.0 equiv.), PEPPSI-IPr (3 mol%), 1,2-DME (0.25 M), 110 °C, 16 h.

c Reaction conditions: 1 (0.50 mmol), 2 (0.60 mmol), Cs_2_CO_3_ (0.75 mmol), SIPrPd(η^3^-1-*t*-Bu-indenyl)Cl (0.005 mmol), H_2_O (2 mL), THF (0.5 mL), 40 °C, 4 h.

d Reaction conditions: 1 (0.7 mmol), 2 (0.735 mmol), NaH (0.735 mmol), 130 °C (oil-bath temperature), 20 h.

Thus, compared with previously reported transition-metal-catalyzed methods, the elaborated method possesses several advantages, the most important of which is exploitation of sodium hydride, one of the simplest and most readily available inorganic bases, whereas catalytic methods require expensive catalysts, bases and solvents. Absence of transition metals, solvents, toxic reagents for carboxylic group activation (*e.g.* carbodiimides) makes proposed protocol a viable alternative for green amide synthesis. At the same time several disadvantages, such as hydrogen evolution and high reaction temperature, should be noted. Therefore, our method is a preparatively useful extension of existing methodology of amide synthesis.

## Conclusions

A general, efficient, green method of aromatic amides synthesis from phenyl esters and aromatic amines under solvent- and transition metal-free conditions using equivalent amounts of NaH as a base was elaborated. Reaction and isolation procedures are simple, robust, easily reproducible, and scalable. The new method is characterized by high atom economy.

## Conflicts of interest

There are no conflicts to declare.

## Supplementary Material

RA-009-C8RA10040C-s001

## References

[cit1] Larock R. C. , Comprehensive Organic Transformations: A Guide to Functional Group Preparations, Wiley, 1999

[cit2] Montalbetti C. A. G. N., Falque V. (2005). Tetrahedron.

[cit3] Pattabiraman V. R., Bode J. W. (2011). Nature.

[cit4] Lanigan R. M., Sheppard T. D. (2013). Eur. J. Org. Chem..

[cit5] Bodanszky M. , in Major Methods of Peptide Bond Formation, ed. E. Gross and J. Meienhofer, Academic Press, 1979, vol. 1, pp. 105–196

[cit6] Valeur E., Bradley M. (2009). Chem. Soc. Rev..

[cit7] de Figueiredo R. M., Suppo J.-S., Campagne J.-M. (2016). Chem. Rev..

[cit8] Krause T., Baader S., Erb B., Gooßen L. J. (2016). Nat. Commun..

[cit9] Gaspa S., Porcheddu A., De Luca L. (2013). Org. Biomol. Chem..

[cit10] Hoerter J. M., Otte K. M., Gellman S. H., Stahl S. S. (2006). J. Am. Chem. Soc..

[cit11] Hoerter J. M., Otte K. M., Gellman S. H., Cui Q., Stahl S. S. (2008). J. Am. Chem. Soc..

[cit12] Acosta-Guzmán P., Mateus-Gómez A., Gamba-Sánchez D. (2018). Molecules.

[cit13] Liu Y., Achtenhagen M., Liu R., Szostak M. (2018). Org. Biomol. Chem..

[cit14] Li G., Szostak M. (2018). Nat. Commun..

[cit15] Becerra-Figueroa L., Ojeda-Porras A., Gamba-Sánchez D. (2014). J. Org. Chem..

[cit16] Banert K. , Aitken K. M., Aitken R. A., Boysen M. M. K. and Bräse S., Science of Synthesis: Houben-Weyl Methods of Molecular Transformations Vol. 41: Nitro, Nitroso, Azo, Azoxy, and Diazonium Compounds, Azides, Triazenes, and Tetrazenes, Thieme, 2014

[cit17] Lima E. C. d., Souza C. C. d., Soares R. d. O., Vaz B. G., Eberlin M. N., Dias A. G., Costa P. R. R. (2011). J. Braz. Chem. Soc..

[cit18] La M. T., Kim H.-K. (2018). Can. J. Chem..

[cit19] Shi S., Nolan S. P., Szostak M. (2018). Acc. Chem. Res..

[cit20] Gnanaprakasam B., Milstein D. (2011). J. Am. Chem. Soc..

[cit21] Ohshima T., Hayashi Y., Agura K., Fujii Y., Yoshiyama A., Mashima K. (2012). Chem. Commun..

[cit22] Han C., Lee J. P., Lobkovsky E., Porco J. A. (2005). J. Am. Chem. Soc..

[cit23] Price K. E., Larrivée-Aboussafy C., Lillie B. M., McLaughlin R. W., Mustakis J., Hettenbach K. W., Hawkins J. M., Vaidyanathan R. (2009). Org. Lett..

[cit24] Bao Y.-S., Zhaorigetu B., Agula B., Baiyin M., Jia M. (2014). J. Org. Chem..

[cit25] Sabot C., Kumar K. A., Meunier S., Mioskowski C. (2007). Tetrahedron Lett..

[cit26] Talvik A.-T., Tuulmets A., Vaino E. (1999). J. Phys. Org. Chem..

[cit27] Heravi M. M., Kheilkordi Z., Zadsirjan V., Heydari M., Malmir M. (2018). J. Organomet. Chem..

[cit28] Ruiz-Castillo P., Buchwald S. L. (2016). Chem. Rev..

[cit29] Surry D. S., Buchwald S. L. (2011). Chem. Sci..

[cit30] Bariwal J., Van der Eycken E. (2013). Chem. Soc. Rev..

[cit31] Burke A. J. and Marques C. S., Catalytic Arylation Methods: From the Academic Lab to Industrial Processes, Wiley, 2015

[cit32] Ben Halima T., Vandavasi J. K., Shkoor M., Newman S. G. (2017). ACS Catal..

[cit33] Shi S., Szostak M. (2017). Chem. Commun..

[cit34] Dardir A. H., Melvin P. R., Davis R. M., Hazari N., Mohadjer Beromi M. (2018). J. Org. Chem..

[cit35] Amaike K., Muto K., Yamaguchi J., Itami K. (2012). J. Am. Chem. Soc..

[cit36] Muto K., Yamaguchi J., Musaev D. G., Itami K. (2015). Nat. Commun..

[cit37] Takise R., Muto K., Yamaguchi J. (2017). Chem. Soc. Rev..

[cit38] Tobisu M., Chatani N. (2015). Acc. Chem. Res..

[cit39] Tollefson E. J., Hanna L. E., Jarvo E. R. (2015). Acc. Chem. Res..

[cit40] Cornella J., Zarate C., Martin R. (2014). Chem. Soc. Rev..

[cit41] Vigante B., Rucins M., Plotniece A., Pajuste K., Luntena I., Cekavicus B., Bisenieks E., Smits R., Duburs G., Sobolev A. (2015). Molecules.

