# Supplementary material for: Solvent- and transition metal-free amide synthesis from phenyl esters and aryl amines
Source: RSC Adv. 2019 Jan 11;9(3):1536–40. doi: 10.1039/c8ra10040c (PMC9059645; doi:10.1039/c8ra10040c)
Supplement: RA-009-C8RA10040C-s001 [file RA-009-C8RA10040C-s001.pdf]

Electronic Supplementary Material (ESI) for RSC Advances.
This journal is © The Royal Society of Chemistry 2018

Solvent- and Transition-Metal-Free Amide Synthesis from Phenyl Esters and Aryl Amines

Sergey A. Rzhevskiy,[a,b] Alexandra A. Ageshina,[a] Gleb A. Chesnokov,[a,b] Pavel S. Gribanov,[a,b] Maxim A. Topchiy,[a,b] Mikhail S. Nechaev*[a,b] and Andrey F. Asachenko*[a,b]

[a] A.V. Topchiev Institute of Petrochemical Synthesis, Russian Academy of Sciences, Leninsky Prospect 29, Moscow, 119991, Russia. E-mail: aasachenko@ips.ac.ru.

[b] M.V. Lomonosov Moscow State University, Leninskie Gory 1 (3), Moscow, 119991, Russia. E-mail: m.s.nechaev@org.chem.msu.ru

Supporting information

Table of Contents

Table of Contents 2

General information 4

General procedure for synthesis of compounds 3a-3dj. 4

N-o-tolylbenzamide (3a) 4

N-phenylbenzamide (3d) 4

N-p-tolylbenzamide (3b) 5

N-(4-nitrophenyl)benzamide (3c) 5

N-(2,6-diisopropylphenyl)benzamide (3e) 5

N-(2,6-diethylphenyl)benzamide (3f) 6

N-(2,4-dimethylphenyl)benzamide (3g) 6

N-(4-methoxyphenyl)benzamide (3h) 7

N-(3-methoxyphenyl)benzamide (3i) 7

N-(3-chlorophenyl)benzamide (3j) 7

N-(4-chlorophenyl)benzamide (3k) 8

N-(4-bromophenyl)benzamide (3l) 8

N-(2-bromophenyl)benzamide (3m) 8

N-benzylbenzamide (3n) 9

N-(4-hydroxyphenyl)benzamide (3o) 9

N-(2-hydroxyphenyl)benzamide (3p) 9

N-(pyridin-4-yl)benzamide (3q) 10

N-(pyridin-2-yl)benzamide (3r) 10

N-(pyrimidin-2-yl)benzamide (3s) 10

N-(3-(trifluoromethyl)phenyl)benzamide (3t) 11

N-(2-fluorophenyl)benzamide (3u) 11

N-(4-fluorophenyl)benzamide (3v) 11

N-(3,5-difluorophenyl)benzamide (3w) 12

N-(3,5-dimethylphenyl)benzamide (3x) 12

N-(naphthalen-1-yl)benzamide (3y) 12

N-(naphthalen-2-yl)benzamide (3z) 13

4-methyl-N-phenylbenzamide (3db) 13

N-phenylpicolinamide (3dc) 13

2,4,6-trimethyl-N-phenylbenzamide (3dd) 14

4-bromo-N-phenylbenzamide (3de) 14

N-phenyldecanamide (3df) 14
N-phenyl-1-naphthamide (3dg) 15
N-phenylfuran-2-carboxamide (3dh)...15
2-(4-methoxyphenyl)-N-phenylacetamide (3di)...15
N-phenylthiophene-2-carboxamide (3dj) 16
References..17
^{1}H and ^{13}C NMR Spectra 19

General information

All arylamines and sodium hydride (60 wt % in mineral oil) were purchased from Sigma-Aldrich. NMR spectra were obtained on a Bruker "Avance 600" (600 MHz ^{1}H, 151 MHz ^{13}C). The chemical shifts are frequency referenced relative to the residual undeuterated solvent peaks. Coupling constants J are given in Hertz as positive values regardless of their real individual signs. The multiplicity of the signals is indicated as ''s", ''d", "t" or ''m" for singlet, doublet, triplet or multiplet, respectively. The abbreviation ''br" is given for broadened signals.

General procedure for synthesis of compounds 3a-3dj.

CAUTION! *Hydrogen gas is evolved during the reaction and proper safety precautions have to be applied. Reactions (at scale larger than indicated below) should not be performed in closed vessels.*

A screw cap vial equipped with magnetic stir bar was charged with 0.7 mmol of phenyl benzoate, 0.735 mmol (1.05 eq.) of arylamine and 29 mg (1.05 eq.) of sodium hydride (60 % dispersion in mineral oil). The reaction vial was placed into a preheated oil bath (130 °C) and allowed to stir (600 RPM) for 20h. The reaction mixture was cooled to room temperature, extracted with EtOAc and washed with water (3x25 ml). The organic extracts were dried over anhydrous sodium sulfate and evaporated to dryness. The solid residue was washed with hexanes followed by dry column vacuum chromatography (EtOAc or EtOAc/hexanes as eluent) or recrystallization (ethanol or EtOAc/hexanes).

Plausible mechanism of reaction[1,2]:

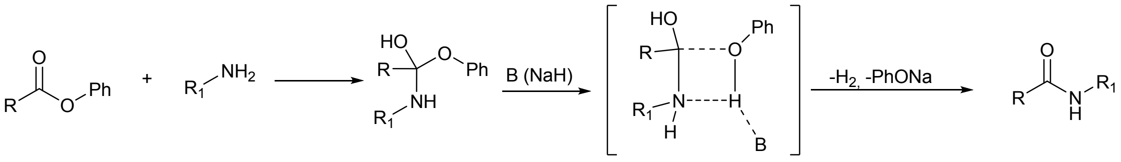

N-o-tolylbenzamide (3a)

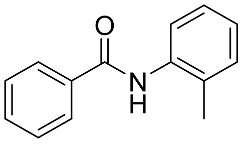

White solid (143 mg, 97 % yield), m.p. 139-141 °C (lit. data: m.p. 143-144 °C).[3]

^{1}H NMR (600 MHz, Chloroform-*d*) δ 7.90-7.89 (m, 3H), 7.79 (s, 1H), 7.56 (t, *J* = 7.4 Hz, 1H), 7.48 (t, *J* = 7.6 Hz, 2H), 7.26 – 7.20 (m, 2H), 7.12 (t, *J* = 7.5 Hz, 1H), 2.32 (s, 3H).

$^{13}C\{^{1}H\}$ NMR (151 MHz, Chloroform-*d*) δ 165.8, 135.9, 135.1, 131.9, 130.7, 129.6, 128.9, 127.2, 126.9, 125.5, 123.4, 17.9.

The NMR data are in agreement with previously reported.[3]

N-phenylbenzamide (3d)

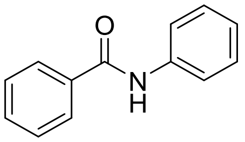

White solid (113 mg, 82 % yield), m.p. 163-165 °C (lit. data: m.p. 163-165 °C).[4]

^{1}H NMR (600 MHz, Chloroform-*d*) δ 7.95 (s, 1H), 7.86 (d, *J* = 7.4 Hz, 2H), 7.65 (d, *J* = 7.8 Hz, 2H), 7.54 (t, *J* = 7.2 Hz, 1H), 7.47 (t, *J* = 7.4 Hz, 2H), 7.36 (t, *J* = 7.6 Hz, 2H), 7.15 (t, *J* = 7.2 Hz, 1H).

^{13}C{^{1}H} NMR (151 MHz, Chloroform-*d*) δ 166.0, 138.1, 135.1, 131.9, 129.2, 128.9, 127.2, 124.7, 120.4.

The NMR data are in agreement with previously reported.[3]

N-p-tolylbenzamide (3b)

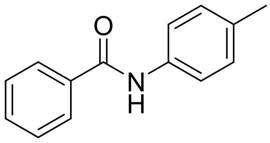

White needles (140.5 mg, 95 % yield), m.p. 148-150 °C (lit. data: m.p. 157-158).[3]

^{1}H NMR (600 MHz, Chloroform-*d*) δ 7.86 (m, 3H), 7.55-7.51 (m, 3H), 7.47 (t, *J* = 7.6 Hz, 2H), 7.17 (d, *J* = 8.1 Hz, 2H), 2.34 (s, 3H).

^{13}C{^{1}H} NMR (151 MHz, Chloroform-*d*) δ 165.8, 135.5, 135.5, 135.2, 135.2, 134.4, 131.9, 129.7, 128.9, 127.1, 120.4, 21.0.

The NMR data are in agreement with previously reported.[5]

N-(4-nitrophenyl)benzamide (3c)

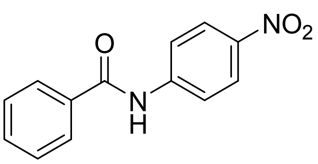

Yellow needles (144 mg, 85 % yield), m.p. 196-198 °C (lit. data: 197-198 °C).[3]

^{1}H NMR (600 MHz, DMSO-d_6) δ 10.81 (s, 1H), 8.27 (d, *J* = 7.4 Hz, 3H), 8.07 (d, *J* = 7.5 Hz, 2H), 7.98 (d, *J* = 7.7 Hz, 2H), 7.64 (t, *J* = 6.7 Hz, 1H), 7.56 (t, *J* = 6.8 Hz, 2H).

^{13}C{^{1}H} NMR (151 MHz, DMSO) δ 166.3, 145.5, 142.5, 134.2, 132.2, 128.5, 127.9, 124.8, 119.8.

The NMR data are in agreement with previously reported.[3]

N-(2,6-diisopropylphenyl)benzamide (3e)

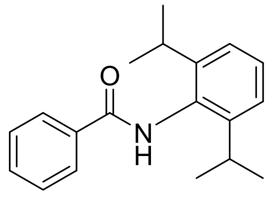

White solid (75 mg, 38 % yield), sublimation at ≤ 210 °C (lit. data: m.p. 257-258 °C).[5]

^{1}H NMR (600 MHz, Chloroform-*d*) δ 7.92 (d, *J* = 7.1 Hz, 2H), 7.57 (t, *J* = 7.4 Hz, 1H), 7.50 (t, *J* = 7.6 Hz, 2H), 7.38 (s, 1H), 7.35 (t, *J* = 7.7 Hz, 1H), 7.23 (d, *J* = 7.7 Hz, 2H), 3.15 (p, *J* = 6.8 Hz, 2H), 1.22 (d, *J* = 6.8 Hz, 12H).

^{13}C{^{1}H} NMR (151 MHz, Chloroform-*d*) δ 167.1, 146.5, 134.8, 131.3, 129.0, 128.6, 127.3, 123.7, 29.1, 23.8.

The NMR data are in agreement with previously reported.[6]

N-(2,6-diethylphenyl)benzamide (3f)

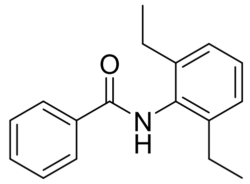

White solid (110 mg, 62 % yield), sublimation at ≤ 170 °C (lit. data: m.p. 236-237 °C).[7]

^{1}H NMR (600 MHz, Chloroform-*d*) δ 7.92 (d, *J* = 7.3 Hz, 2H), 7.57 (t, *J* = 7.1 Hz, 1H), 7.50 (t, *J* = 7.5 Hz, 2H), 7.41 (s, 1H), 7.29 – 7.24 (m, 1H), 7.17 (d, *J* = 7.5 Hz, 2H), 2.65 (q, *J* = 7.5 Hz, 4H), 1.21 (t, *J* = 7.5 Hz, 6H).

^{13}C{^{1}H} NMR (151 MHz, Chloroform-*d*) δ 166.7, 141.8, 134.7, 132.8, 131.9, 128.9, 128.2, 127.3, 126.5, 25.1, 14.6.

The NMR data are in agreement with previously reported.[5]

N-(2,4-dimethylphenyl)benzamide (3g)

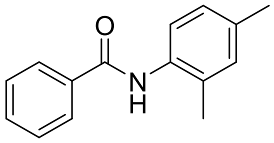

White needles (131 mg, 83 % yield), m.p. 175-180 °C (lit. data: m.p. 193-194 °C).[8]

^{1}H NMR (600 MHz, Chloroform-*d*) δ 7.88 (d, *J* = 7.1 Hz, 2H), 7.80 – 7.70 (m, 1H), 7.62 (s, 1H), 7.55 (t, *J* = 7.3 Hz, 1H), 7.49 (t, *J* = 7.4 Hz, 2H), 7.07-7.05 (m, 2H), 2.32 (s, 3H), 2.30 (s, 3H).

$^{13}C\{^1H\}$ NMR (151 MHz, Chloroform-*d*) δ 165.8, 135.3, 135.2, 133.3, 131.9, 131.4, 129.8, 128.9, 127.5, 127.2, 123.6, 21.0, 17.9.

The NMR data are in agreement with previously reported.[8]

N-(4-methoxyphenyl)benzamide (3h)

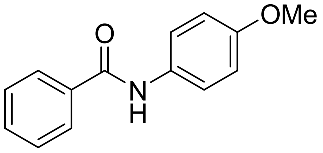

White crystals (135 mg, 85 % yield), m.p. 156-157 °C (lit. data: m.p. 157-158 °C).[8]

^1H NMR (600 MHz, Chloroform-*d*) δ 7.86 (d, *J* = 7.4 Hz, 2H), 7.80 (s, 1H), 7.53 (t, *J* = 7.0 Hz, 3H), 7.47 (t, *J* = 7.5 Hz, 2H), 6.90 (d, *J* = 8.8 Hz, 2H), 3.81 (s, 3H).

$^{13}C\{^1H\}$ NMR (151 MHz, Chloroform-*d*) δ 156.8, 135.2, 131.8, 131.2, 128.9, 127.1, 122.3, 114.4, 55.7.

The NMR data are in agreement with previously reported.[8]

N-(3-methoxyphenyl)benzamide (3i)

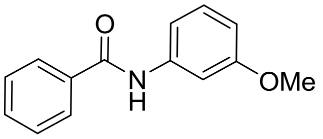

White solid (192 mg, >99 % yield), m.p. 111-113 °C (lit. data: m.p. 112-114 °C).[9]

^1H NMR (600 MHz, Chloroform-*d*) δ 7.96 (s, 1H), 7.85 (d, *J* = 7.6 Hz, 2H), 7.53 (t, *J* = 7.2 Hz, 1H), 7.48 – 7.41 (m, 3H), 7.24 (t, *J* = 8.1 Hz, 1H), 7.11 (d, *J* = 7.8 Hz, 1H), 6.71 (d, *J* = 8.1 Hz, 1H), 3.81 (s, 3H).

$^{13}C\{^1H\}$ NMR (151 MHz, Chloroform-*d*) δ 165.9, 160.4, 139.3, 135.1, 132.0, 129.8, 128.9, 127.1, 112.5, 110.7, 106.0, 55.5.

The NMR data are in agreement with previously reported.[9]

N-(3-chlorophenyl)benzamide (3j)

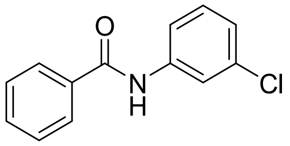

Colorless needles (156 mg, 96 % yield), m.p. 116-118 °C (lit. data: m.p. 116-117 °C).[10]

^1H NMR (600 MHz, Chloroform-*d*) δ 8.05 (s, 1H), 7.84 (d, *J* = 7.3 Hz, 2H), 7.77 (s, 1H), 7.54 (t, *J* = 7.4 Hz, 1H), 7.47 (dt, *J* = 15.4, 7.6 Hz, 3H), 7.28 – 7.23 (m, 1H), 7.12 (d, *J* = 7.9 Hz, 1H).

$^{13}C\{^1H\}$ NMR (151 MHz, Chloroform-*d*) δ 166.1, 139.2, 134.8, 134.6, 132.2, 130.1, 128.9, 127.18, 124.7, 120.5, 118.4.

The NMR data are in agreement with previously reported.[10]

N-(4-chlorophenyl)benzamide (3k)

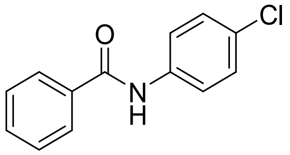

White needles (136 mg, 84 % yield), m.p. 186-187 °C (lit. data: m.p. 188-190 °C).[11]

^1H NMR (600 MHz, DMSO-d_6) δ 10.37 (s, 1H), 8.01 – 7.93 (m, 2H), 7.83 (d, J = 8.8 Hz, 2H), 7.60 (t, J = 7.3 Hz, 1H), 7.53 (t, J = 7.6 Hz, 2H), 7.41 (d, J = 8.8 Hz, 2H).

$^{13}C\{^1H\}$ NMR (151 MHz, DMSO-d_6) δ 165.7, 138.2, 134.7, 131.7, 128.5, 128.4, 127.7, 127.3, 121.8.

The NMR data are in agreement with previously reported.[10]

N-(4-bromophenyl)benzamide (3l)

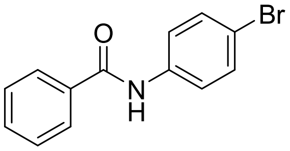

White solid (192 mg, >99 % yield), m.p. 202-203 °C (lit. data: m.p. 203-204 °C).[4]

^1H NMR (600 MHz, DMSO-d_6) δ 10.39 (s, 1H), 7.95 (d, J = 7.3 Hz, 2H), 7.78 (d, J = 8.8 Hz, 2H), 7.60 (t, J = 7.3 Hz, 1H), 7.56 – 7.51 (m, 4H).

$^{13}C\{^1H\}$ NMR (151 MHz, DMSO-d_6) δ 165.7, 138.6, 134.7, 131.8, 131.5, 128.5, 127.7, 122.2, 115.4.

The NMR data are in agreement with previously reported.[4]

N-(2-bromophenyl)benzamide (3m)

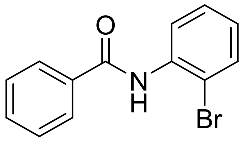

White needles (89 mg, 46 % yield), m.p. 94-95 °C (lit. data: m.p. 107-108 °C).[12]

^1H NMR (600 MHz, Chloroform-*d*) δ 8.59 – 8.54 (m, 1H), 8.47 (s, 1H), 7.94 (d, J = 7.1 Hz, 2H), 7.61 – 7.56 (m, 2H), 7.53 (t, J = 7.5 Hz, 2H), 7.40 – 7.35 (m, 1H), 7.05 – 6.99 (m, 1H).

$^{13}C\{^1H\}$ (151 MHz, Chloroform-*d*) δ 165.4, 136.0, 134.7, 132.4, 132.3, 129.1, 128.7, 127.2, 125.4, 121.9, 113.9.

The NMR data are in agreement with previously reported.[12]

N-benzylbenzamide (3n)

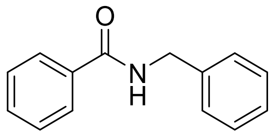

White solid (130 mg, 88 % yield), m.p. 98 °C (lit. data: m.p. 98-100 °C).[13]

^{1}H NMR (600 MHz, Chloroform-*d*) δ 7.79 (d, *J* = 7.4 Hz, 2H), 7.49 (t, *J* = 7.4 Hz, 1H), 7.41 (t, *J* = 7.7 Hz, 2H), 7.34 (d, *J* = 4.3 Hz, 4H), 7.31 – 7.27 (m, 1H), 6.60 (s, 1H), 4.63 (d, *J* = 5.7 Hz, 2H).

^{13}C{^{1}H} NMR (151 MHz, Chloroform-*d*) δ 167.5, 138.4, 134.5, 131.6, 128.9, 128.7, 128.0, 127.7, 127.1, 44.2

The NMR data are in agreement with previously reported.[14]

N-(4-hydroxyphenyl)benzamide (3o)

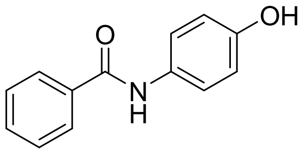

White solid (107 mg, 72 % yield), m.p. 209-210 °C (lit. data: m.p. 207-209 °C.[15]

^{1}H NMR (600 MHz, DMSO-d_6) δ 10.02 (s, 1H), 9.25 (s, 1H), 7.93 (d, *J* = 7.3 Hz, 2H), 7.58 – 7.48 (m, 5H), 6.75 (d, *J* = 8.8 Hz, 2H).

^{13}C{^{1}H} NMR (151 MHz, DMSO-d_6) δ 165.0, 153.7, 135.2, 131.3, 130.7, 128.3, 127.5, 122.3, 115.00.

The NMR data are in agreement with previously reported.[15]

N-(2-hydroxyphenyl)benzamide (3p)

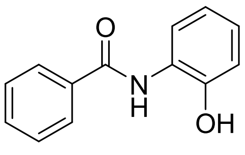

Light pink flakes (119 mg, 80 % yield), m.p. 163-164 °C (lit. data: m.p. 168-169 °C).[16]

^{1}H NMR (600 MHz, DMSO-d_6) δ 9.72 (s, 1H), 9.50 (s, 1H), 7.98 (d, *J* = 7.3 Hz, 2H), 7.70 (d, *J* = 7.5 Hz, 1H), 7.60 (t, *J* = 7.3 Hz, 1H), 7.53 (t, *J* = 7.5 Hz, 2H), 7.04 (t, *J* = 7.7 Hz, 1H), 6.93 (d, *J* = 8.0 Hz, 1H), 6.84 (t, *J* = 7.6 Hz, 1H).

^{13}C{^{1}H} NMR (151 MHz, DMSO-d_6) δ 165.2, 149.3, 134.4, 131.6, 128.5, 127.5, 125.9, 125.6, 124.0, 119.0, 116.0.

The NMR data are in agreement with previously reported.[17]

N-(pyridin-4-yl)benzamide (3q)

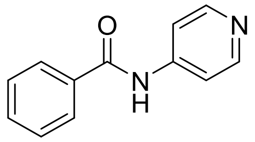

White solid (100 mg, 72 % yield), m.p. 204-206 °C (lit. data: m.p. 209-211 °C).[18]

^{1}H NMR (600 MHz, Chloroform-*d*) δ 8.54 (d, *J* = 5.6 Hz, 2H), 8.10 (s, 1H), 7.88 (d, *J* = 7.3 Hz, 2H), 7.62 (d, *J* = 5.9 Hz, 2H), 7.59 (t, *J* = 7.3 Hz, 1H), 7.51 (t, *J* = 7.6 Hz, 2H).

^{13}C{^{1}H} (151 MHz, Chloroform-*d*) δ 166.3, 150.9, 145.2, 134.2, 132.7, 129.1, 127.3, 114.0.

The NMR data are in agreement with previously reported.[18]

N-(pyridin-2-yl)benzamide (3r)

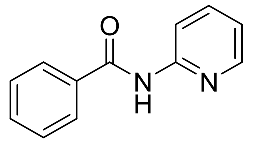

Colorless needles (111 mg, 80 % yield), m.p. 76-78 °C (lit. data: m.p. 76-78 °C).[19]

^{1}H NMR (600 MHz, Chloroform-*d*) δ 9.10 (br. m, 1H), 8.44 (d, *J* = 8.4 Hz, 1H), 8.23 (s, 1H), 7.95 (d, *J* = 7.7 Hz, 2H), 7.79 (t, *J* = 7.9 Hz, 1H), 7.57 (t, *J* = 7.4 Hz, 1H), 7.49 (t, *J* = 7.7 Hz, 2H), 7.10 – 7.05 (m, 1H).

^{13}C{^{1}H} NMR (151 MHz, Chloroform-*d*) δ 166.0, 151.6, 147.2, 139.1, 134.2, 132.5, 129.0, 127.5, 120.0, 114.6.

The NMR data are in agreement with previously reported.[19]

N-(pyrimidin-2-yl)benzamide (3s)

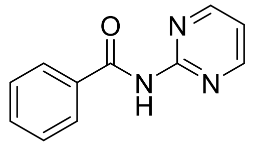

Light yellow needles (85 mg, 61 % yield), m.p. 138 °C (lit. data: m.p. 140-142 °C).[20]

^{1}H NMR (600 MHz, Chloroform-*d*) δ 9.00 (s, 1H), 8.60 (d, *J* = 4.4 Hz, 2H), 7.93 (d, *J* = 8.1 Hz, 2H), 7.56 (t, *J* = 7.0 Hz, 1H), 7.47 (t, *J* = 7.6 Hz, 2H), 7.03 (t, *J* = 4.6 Hz, 1H).

^{13}C{^{1}H} NMR (151 MHz, Chloroform-*d*) δ 165.1, 158.5, 157.9, 134.4, 132.5, 128.9, 127.66, 116.9.

The NMR data are in agreement with previously reported.[21]

N-(3-(trifluoromethyl)phenyl)benzamide (3t)

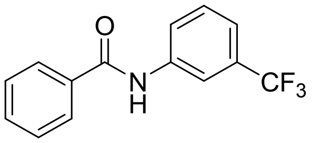

White solid (156 mg, 84 % yield), m.p. 111-113 °C (lit. data: m.p. 88-89 °C).[22]

^{1}H NMR (600 MHz, Chloroform-*d*) δ 8.20 (s, 1H), 7.94 (s, 1H), 7.88 – 7.82 (m, 3H), 7.54 (t, *J* = 7.4 Hz, 1H), 7.47 – 7.41 (m, 3H), 7.39 (d, *J* = 7.5 Hz, 1H).

^{13}C{^{1}H} NMR (151 MHz, Chloroform-*d*) δ 166.24, 138.6, 134.5, 132.3, 131.5 (q, J = 32 Hz), 129.7, 129.0, 127.2, 124.0 (q, J = 270 Hz), 123.5, 121.23 (q, *J* = 3.6 Hz), 117.18 (q, *J* = 3.9 Hz).

The NMR data are in agreement with previously reported.[22]

N-(2-fluorophenyl)benzamide (3u)

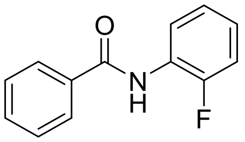

White needles (131 mg, 87 % yield), m.p. 102-103 °C (lit. data: m.p. 108-109 °C [22]).

^{1}H NMR (600 MHz, Chloroform-*d*) δ 8.47 (t, *J* = 7.5 Hz, 1H), 8.09 (s, 1H), 7.89 (d, *J* = 7.3 Hz, 2H), 7.57 (t, *J* = 7.2 Hz, 1H), 7.50 (t, *J* = 7.5 Hz, 2H), 7.19 (t, *J* = 7.3 Hz, 1H), 7.16 – 7.07 (m, 2H).

^{13}C{^{1}H} NMR (151 MHz, Chloroform-*d*) δ 165.6, 152.8 (d, *J* = 243.1 Hz), 134.7, 132.2, 129.0, 127.2, 126.6 (d, *J* = 9.7 Hz), 124.8 (d, *J* = 3.5 Hz), 124.6 (d, *J* = 7.8 Hz), 122.0, 115.0 (d, *J* = 19.2 Hz).

The NMR data are in agreement with previously reported.[22].

N-(4-fluorophenyl)benzamide (3v)

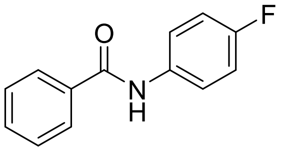

White crystals (149 mg, >99 % yield), m.p. 175-176 °C (lit. data: m.p. 175-177 °C).[16]

^{1}H NMR (600 MHz, DMSO-*d*$_6$) δ 10.31 (s, 1H), 7.95 (d, *J* = 7.1 Hz, 2H), 7.83 – 7.77 (m, 2H), 7.59 (t, *J* = 7.3 Hz, 1H), 7.53 (t, *J* = 7.5 Hz, 2H), 7.19 (t, *J* = 8.9 Hz, 2H).

^{13}C{^{1}H} NMR (151 MHz, DMSO-*d*$_6$) δ 165.5, 158.3 (d, *J* = 240.2 Hz), 135.6 (d, *J* = 2.4 Hz), 134.8, 131.6, 128.4, 127.7, 122.2 (d, *J* = 7.7 Hz), 115.2 (d, *J* = 22.0 Hz).

The NMR data are in agreement with previously reported.[16].

N-(3,5-difluorophenyl)benzamide (3w)

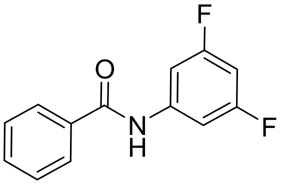

Colorless crystals (125 mg, 77 % yield), m.p. 145-146 °C (lit. data: m.p. 145.8 -146.9 °C).[23]

^{1}H NMR (600 MHz, Chloroform-*d*) δ 8.01 (s, 1H), 7.82 (d, *J* = 6.9 Hz, 2H), 7.55 (t, *J* = 6.4 Hz, 1H), 7.47 (t, *J* = 6.7 Hz, 2H), 7.30 – 7.23 (m, 2H), 6.59 (t, *J* = 7.6 Hz, 1H).

^{13}C{^{1}H} NMR (151 MHz, Chloroform-*d*) δ 166.0, 163.4 (dd, *J* = 246.6, 14.7 Hz), 140.3 (t, *J* = 13.6 Hz), 134.4, 132.5, 129.1, 127.2, 103.4 – 103.2 (m), 99.9 (t, *J* = 25.7 Hz).

The NMR data are in agreement with previously reported.[23]

N-(3,5-dimethylphenyl)benzamide (3x)

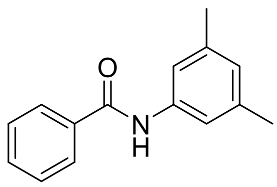

White needles (120 mg, 76 % yield), m.p. 142-143 °C. (lit. data: 144-147 °C [21][24]).

^{1}H NMR (600 MHz, Chloroform-*d*) δ 7.88 – 7.82 (m, 3H), 7.53 (t, *J* = 7.4 Hz, 1H), 7.46 (t, *J* = 7.6 Hz, 2H), 7.28 (s, 2H), 6.79 (s, 1H), 2.31 (s, 6H).

^{13}C NMR (151 MHz, Chloroform-*d*) δ 165.9, 138.9, 137.8, 135.18, 131.98, 128.8, 127.1, 126.4, 118.0, 21.5.

The NMR data are in agreement with previously reported.[25]

N-(naphthalen-1-yl)benzamide (3y)

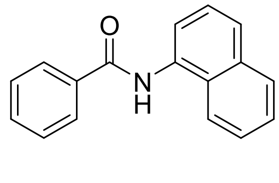

White needles (125 mg, 72 % yield), m.p. 155-156 °C (lit. data: m.p. 167-168 °C).[26]

^{1}H NMR (600 MHz, Chloroform-*d*) δ 8.31 (s, 1H), 8.03 – 7.90 (m, 3H), 7.90 – 7.86 (m, 2H), 7.73 (d, *J* = 8.2 Hz, 1H), 7.57 (t, *J* = 7.3 Hz, 1H), 7.53 – 7.45 (m, 5H).

^{13}C{^{1}H} NMR (151 MHz, Chloroform-*d*) δ 166.4, 134.8, 134.2, 132.5, 132.0, 128.9, 128.9, 127.7, 127.3, 126.5, 126.3, 126.1, 125.8, 121.6, 121.0.

The NMR data are in agreement with previously reported.[26]

N-(naphthalen-2-yl)benzamide (3z)

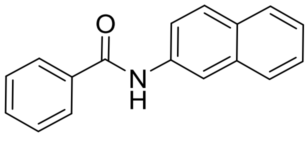

Colorless crystals (166 mg, 96 % yield), m.p. 157-158 °C (lit. data: m.p. 156-159 °C).[27]

^{1}H NMR (600 MHz, Chloroform-*d*) δ 8.33 (s, 1H), 8.10 (s, 1H), 7.91 (d, *J* = 7.3 Hz, 2H), 7.84 – 7.78 (m, 3H), 7.60 (dd, *J* = 8.7, 1.9 Hz, 1H), 7.55 (t, *J* = 7.4 Hz, 1H), 7.51 – 7.45 (m, 3H), 7.45 – 7.40 (m, 1H).

^{13}C{^{1}H}NMR (151 MHz, Chloroform-*d*) δ 166.1, 135.5, 135.1, 134.0, 132.0, 130.9, 129.0, 128.9, 127.9, 127.7, 127.2, 126.7, 125.3, 120.3, 117.3.

The NMR data are in agreement with previously reported.[28]

4-methyl-N-phenylbenzamide (3db)

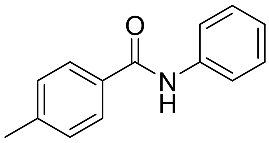

White needles (120 mg, 81 % yield), m.p. 137-138 °C (lit. data: m.p. 143-144 °C).[3]

^{1}H NMR (600 MHz, Chloroform-*d*) δ 7.99 (s, 1H), 7.76 (d, *J* = 8.0 Hz, 2H), 7.64 (d, *J* = 8.1 Hz, 2H), 7.34 (t, *J* = 7.8 Hz, 2H), 7.24 (d, *J* = 7.9 Hz, 2H), 7.13 (t, *J* = 7.4 Hz, 1H), 2.41 (s, 3H).

^{13}C{^{1}H} NMR (151 MHz, Chloroform-*d*) δ 165.9, 142.4, 138.2, 132.2, 129.5, 129.1, 127.2, 124.5, 120.4, 21.6.

The NMR data are in agreement with previously reported.[3]

N-phenylpicolinamide (3dc)

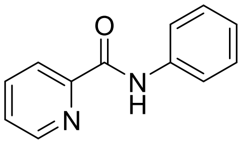

Yellow solid. (119 mg, 86 % yield), m.p. 76-77 °C (lit. data : m.p. 74-75 °C).[3]

^{1}H NMR (600 MHz, Chloroform-*d*) δ 10.04 (s, 1H), 8.60 (d, *J* = 4.1 Hz, 1H), 8.30 (d, *J* = 7.8 Hz, 1H), 7.89 (td, *J* = 7.7, 1.6 Hz, 1H), 7.79 (d, *J* = 7.7 Hz, 2H), 7.49 – 7.43 (m, 1H), 7.39 (t, *J* = 7.9 Hz, 2H), 7.15 (t, *J* = 7.4 Hz, 1H).

^{13}C NMR (151 MHz, Chloroform-*d*) δ 162.1, 149.9, 148.0, 137.8, 137.8, 129.2, 126.5, 124.4, 122.5, 119.8

The NMR data are in agreement with previously reported.[3]

2,4,6-trimethyl-N-phenylbenzamide (3dd)

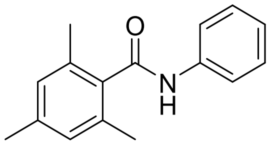

White needles. (70 mg, 42 % yield), m.p. 164-166 °C (lit. data: m.p. 166-167 °C).[29]

^{1}H NMR (600 MHz, Chloroform-*d*) δ 7.61 (d, *J* = 7.5 Hz, 2H), 7.45 (s, 1H), 7.36 (t, *J* = 7.9 Hz, 2H), 7.16 (t, *J* = 7.4 Hz, 1H), 6.88 (s, 2H), 2.34 (s, 6H), 2.31 (s, 3H).

^{13}C NMR (151 MHz, Chloroform-*d*) δ 169.7, 159.2, 137.8, 130.8, 129.1, 126.4, 124.5, 119.9, 114.8, 55.5, 44.19.

The NMR data are in agreement with previously reported.[6]

4-bromo-N-phenylbenzamide (3de)

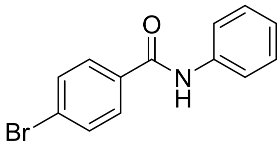

White solid. (156.5 mg, 81 % yield), m.p. 199-201°C (lit. data: m.p. 202-204 °C).[3]

^{1}H NMR (600 MHz, DMSO-d_6) δ 10.30 (s, 1H), 7.91 (d, *J* = 8.5 Hz, 2H), 7.75 (t, *J* = 8.2 Hz, 4H), 7.35 (t, *J* = 8.0 Hz, 2H), 7.11 (t, *J* = 7.4 Hz, 1H).

^{13}C NMR (151 MHz, DMSO) δ 164.5, 138.9, 134.0, 131.4, 129.8, 128.6, 125.3, 123.8, 120.4.

The NMR data are in agreement with previously reported.[3]

N-phenyldecanamide (3df)

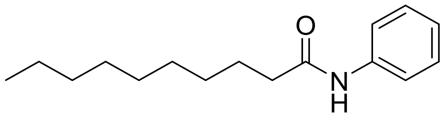

White solid (121 mg, 70 % yield), m.p. 63-64 °C (lit. data: m.p. 69 °C).[30]

^{1}H NMR (600 MHz, Chloroform-*d*) δ 7.52 (d, *J* = 7.9 Hz, 2H), 7.42 (s, 1H), 7.30 (t, *J* = 7.8 Hz, 2H), 7.09 (t, *J* = 7.3 Hz, 1H), 2.34 (t, *J* = 7.6 Hz, 2H), 1.71 (p, *J* = 7.5 Hz, 2H), 1.37 – 1.22 (m, 12H), 0.88 (t, *J* = 7.0 Hz, 3H).

^{13}C NMR (151 MHz, Chloroform-*d*) δ 171.7, 138.1, 129.0, 124.3, 120.0, 37.9, 32.0, 29.6, 29.5, 29.4, 29.4, 25.8, 22.8, 14.2.

The NMR data are in agreement with previously reported.[31]

N-phenyl-1-naphthamide (3dg)

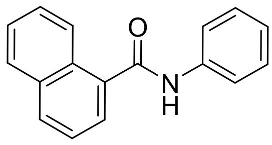

White solid (135 mg, 80 % yield), m.p. 156-158 °C (lit. data: m.p. 164-165 °C).[3]

^{1}H NMR (600 MHz, DMSO-d_6) δ 10.58 (s, 1H), 8.23 – 8.16 (m, 1H), 8.08 (d, *J* = 8.1 Hz, 1H), 8.05 – 7.99 (m, 1H), 7.83 (d, *J* = 7.5 Hz, 2H), 7.76 (d, *J* = 6.4 Hz, 1H), 7.65 – 7.56 (m, 3H), 7.38 (t, *J* = 7.7 Hz, 2H), 7.13 (t, *J* = 7.3 Hz, 1H).

^{13}C{^{1}H} NMR (151 MHz, DMSO-d_6) δ 167.3, 139.3, 134.8, 133.1, 130.1, 129.6, 128.7, 128.3, 127.0, 126.3, 125.4, 125.1, 125.0, 123.7, 119.8.

The NMR data are in agreement with previously reported.[3]

N-phenylfuran-2-carboxamide (3dh)

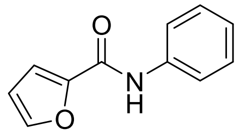

Colorless crystals. (118 mg, 90 % yield), m.p. 120-122 °C (lit. data: m.p. 123-124 °C).[4]

^{1}H NMR (600 MHz, Chloroform-*d*) δ 8.10 (s, 1H), 7.65 (d, *J* = 7.7 Hz, 2H), 7.51 (s, 1H), 7.36 (t, *J* = 7.9 Hz, 2H), 7.24 (d, *J* = 3.4 Hz, 1H), 7.14 (t, *J* = 7.4 Hz, 1H), 6.55 (dd, *J* = 3.4, 1.7 Hz, 1H).

^{13}C NMR (151 MHz, Chloroform-*d*) δ 156.2, 147.9, 144.3, 137.5, 129.2, 124.7, 120.1, 115.4, 112.8.

The NMR data are in agreement with previously reported.[32]

2-(4-methoxyphenyl)-N-phenylacetamide (3di)

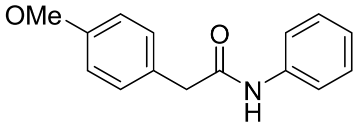

White needles. (99 mg, 67 % yield), m.p. 114-115 °C (lit. data: m.p. 115-117 °C).[33]

^{1}H NMR (600 MHz, Chloroform-*d*) δ 7.41 (d, *J* = 7.9 Hz, 2H), 7.30 – 7.22 (m, 4H), 7.16 (s, 1H), 7.08 (t, *J* = 7.3 Hz, 1H), 6.93 (d, *J* = 8.1 Hz, 2H), 3.83 (s, 3H), 3.68 (s, 2H).

^{13}C NMR (151 MHz, Chloroform-*d*) δ 169.7, 159.2, 137.8, 130.8, 129.1, 126.4, 124.5, 119.9, 114.8, 55.5, 44.1.

The NMR data are in agreement with previously reported.[33]

N-phenylthiophene-2-carboxamide (3dj)

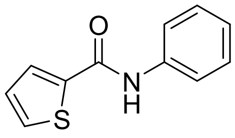

White solid. (119.5 mg, 84 % yield), m.p. 142-144 °C (lit. data: m.p. 140 – 142 °C).[34]

^{1}H NMR (600 MHz, DMSO-d_6) δ 10.21 (s, 1H), 8.02 (dd, J = 3.7, 1.0 Hz, 1H), 7.85 (dd, J = 5.0, 1.0 Hz, 1H), 7.72 (d, J = 7.6 Hz, 2H), 7.35 (t, J = 7.9 Hz, 2H), 7.22 (dd, J = 4.9, 3.8 Hz, 1H), 7.10 (t, J = 7.4 Hz, 1H).

^{13}C NMR (151 MHz, DMSO) δ 159.9, 140.1, 138.7, 131.8, 129.1, 128.7, 128.1, 123.8, 120.4.

The NMR data are in agreement with previously reported.[35]

References

1. A. S. A. S. Shawali and S. S. Biechler, *J. Am. Chem. Soc.*, 1967, **89**, 3020-3026.
2. A.-T. Talvik, A. Tuulmets and E. Vaino, *J. Phys. Org. Chem.*, 1999, **12**, 747-750.
3. Y. Wang, D. Zhu, L. Tang, S. Wang and Z. Wang, *Angew. Chem. Int. Ed.*, 2011, **50**, 8917-8921.
4. C. K. Lee, J. S. Yu and Y. R. Ji, *J. Heterocycl. Chem.*, 2009, **39**, 1219-1227.
5. Y. Wu, S. Wang, L. Zhang, G. Yang, X. Zhu, Z. Zhou, H. Zhu and S. Wu, *Eur. J. Org. Chem.*, 2009, **2010**, 326-332.
6. L. Zhang, S. Su, H. Wu and S. Wang, *Tetrahedron*, 2009, **65**, 10022-10024.
7. P. M. G. Bavin and J. M. W. Scott, *Can. J. Chem.*, 1958, **36**, 1284-1288.
8. M. A. Ali, P. Saha and T. Punniyamurthy, *Synthesis*, 2010, **2010**, 908-910.
9. K. Dooleweerdt, B. P. Fors and S. L. Buchwald, *Org. Lett.*, 2010, **12**, 2350-2353.
10. S. Ueda and H. Nagasawa, *J. Org. Chem.*, 2009, **74**, 4272-4277.
11. Z. Zhang, Y. Yu and L. S. Liebeskind, *Org. Lett.*, 2008, **10**, 3005-3008.
12. G. Evindar and R. A. Batey, *J. Org. Chem.*, 2006, **71**, 1802-1808.
13. E. Y. Kusei, M. S. Novikov and A. F. Khlebnikov, *Russ. J. Gen. Chem.*, 2005, **75**, 1643-1647.
14. M. A. Mohamed, K.-i. Yamada and K. Tomioka, *Tetrahedron Lett.*, 2009, **50**, 3436-3438.
15. J.-E. Won, H.-K. Kim, J.-J. Kim, H.-S. Yim, M.-J. Kim, S.-B. Kang, H.-A. Chung, S.-G. Lee and Y.-J. Yoon, *Tetrahedron*, 2007, **63**, 12720-12730.
16. C. A. Faler and M. M. Joullié, *Tetrahedron Lett.*, 2006, **47**, 7229-7231.
17. D. Yang and H. Fu, *Chem. - Eur. J.*, 2010, **16**, 2366-2370.
18. A. Jóźwiak, Jacek Z. Brzeziński, Mieczysław W. Płotka, Aleksandra K. Szcześniak, Z. Malinowski and J. Epsztajn, *Eur. J. Org. Chem.*, 2004, **2004**, 3254-3261.
19. A. R. Katritzky, B. E.-D. M. El-Gendy, E. Todadze and A. A. A. Abdel-Fattah, *J. Org. Chem.*, 2008, **73**, 5442-5445.
20. Y. Li, Y. Wang and J. Wang, *Heterocycl. Commun.*, 2007, **13**, 251.
21. M. Vimolratana, J. L. Simard and S. P. Brown, *Tetrahedron Lett.*, 2011, **52**, 1020-1022.
22. P. J. Tambade, Y. P. Patil, M. J. Bhanushali and B. M. Bhanage, *Synthesis*, 2008, **2008**, 2347-2352.
23. P. Mocilac, I. A. Osman and J. F. Gallagher, *CrystEngComm*, 2016, **18**, 5764-5776.
24. C. Tietcheu, C. Garcia, D. Gardette, D. Dugat and J.-C. Gramain, *J. Heterocycl. Chem.*, 2009, **39**, 965-973.
25. S. M. Johnson, S. Connelly, I. A. Wilson and J. W. Kelly, *J. Med. Chem.*, 2008, **51**, 6348-6358.
26. H. Al-Awadi, M. R. Ibrahim, H. H. Dib, N. A. Al-Awadi and Y. A. Ibrahim, *Tetrahedron*, 2005, **61**, 10507-10513.
27. R. Huisgen, *Justus Liebigs Ann. Chem.*, 1949, **564**, 16-32.
28. C.-T. Chen, J.-H. Kuo, V. D. Pawar, Y. S. Munot, S.-S. Weng, C.-H. Ku and C.-Y. Liu, *J. Org. Chem.*, 2005, **70**, 1188-1197.
29. H. Suzuki, J. Tsuji, Y. Hiroi, N. Sato and A. Osuka, *Chem. Lett.*, 1983, **12**, 449-452.
30. J. Kennedy, A. Lewis, N. J. McCorkindale and R. A. Raphael, *J. Chem. Soc. (Resumed)*, 1961, 4945-4948.
31. S. Shi and M. Szostak, *Chem. Commun.*, 2017, **53**, 10584-10587.
32. C. H. Kee, A. Ariffin, K. Awang, K. Takeya, H. Morita, S. I. Hussain, K. M. Chan, P. J. Wood, M. D. Threadgill, C. G. Lim, S. Ng, J. F. F. Weber and N. F. Thomas, *Org. Biomol. Chem.*, 2010, **8**, 5646-5660.
33. W. Yu, S. Yang, F. Xiong, T. Fan, Y. Feng, Y. Huang, J. Fu and T. Wang, *Org. Biomol. Chem.*, 2018, **16**, 3099-3103.

34. T. Kitagawa, H. Kuroda, H. Sasaki and K. Kawasaki, *Chem. Pharm. Bull.*, 1987, **35**, 4294-4301.
35. K. N. Kumar, K. Sreeramamurthy, S. Palle, K. Mukkanti and P. Das, *Tetrahedron Lett.*, 2010, **51**, 899-902.

^{1}H and ^{13}C NMR Spectra

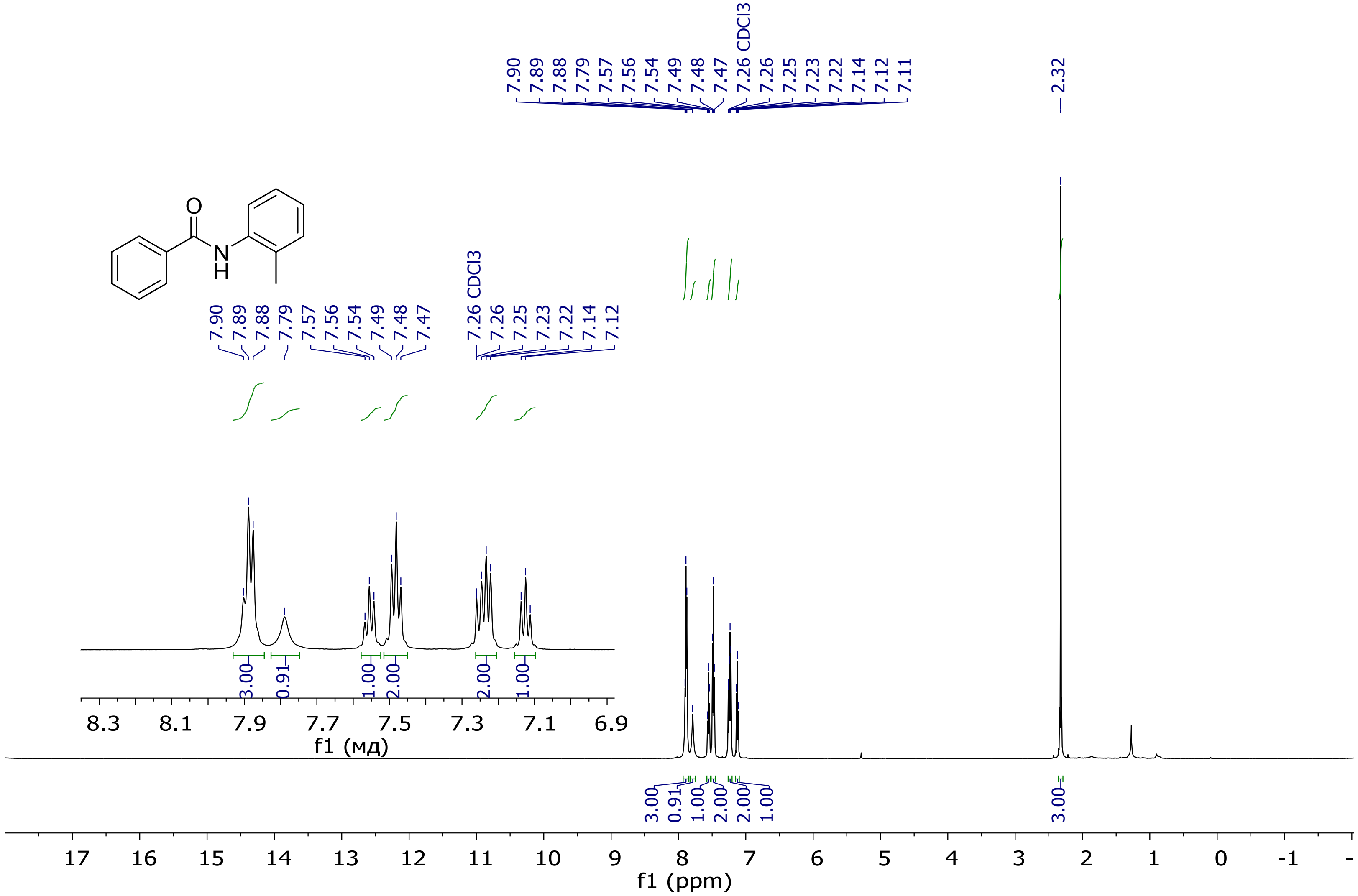

Figure S1. ^{1}H NMR (600 MHz, Chloroform-*d*) of N-o-tolylbenzamide (3a).

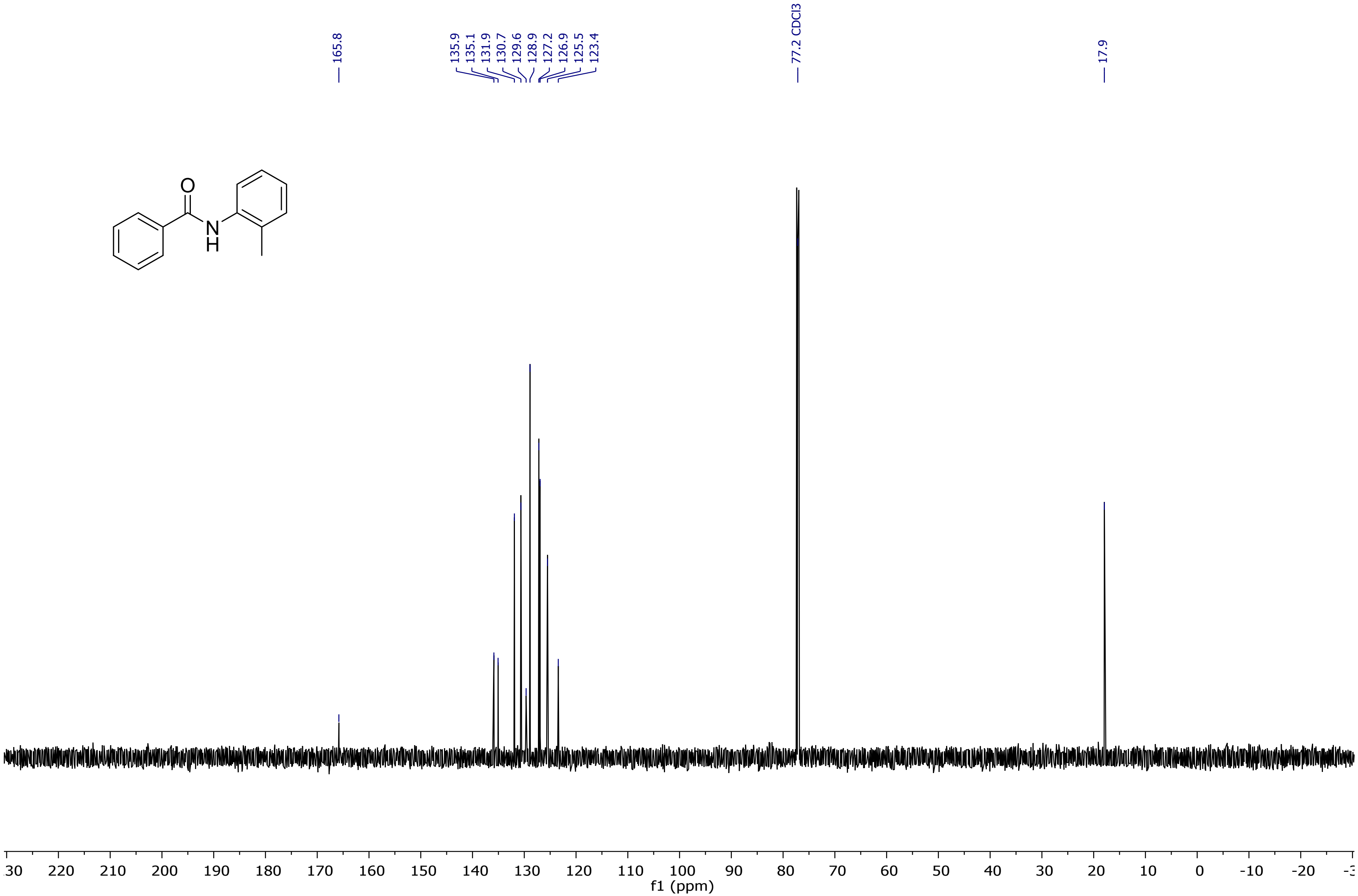

Figure S2. $^{13}C\{^{1}H\}$ **NMR (151 MHz, Chloroform-d) of N-o-tolylbenzamide (3a).**

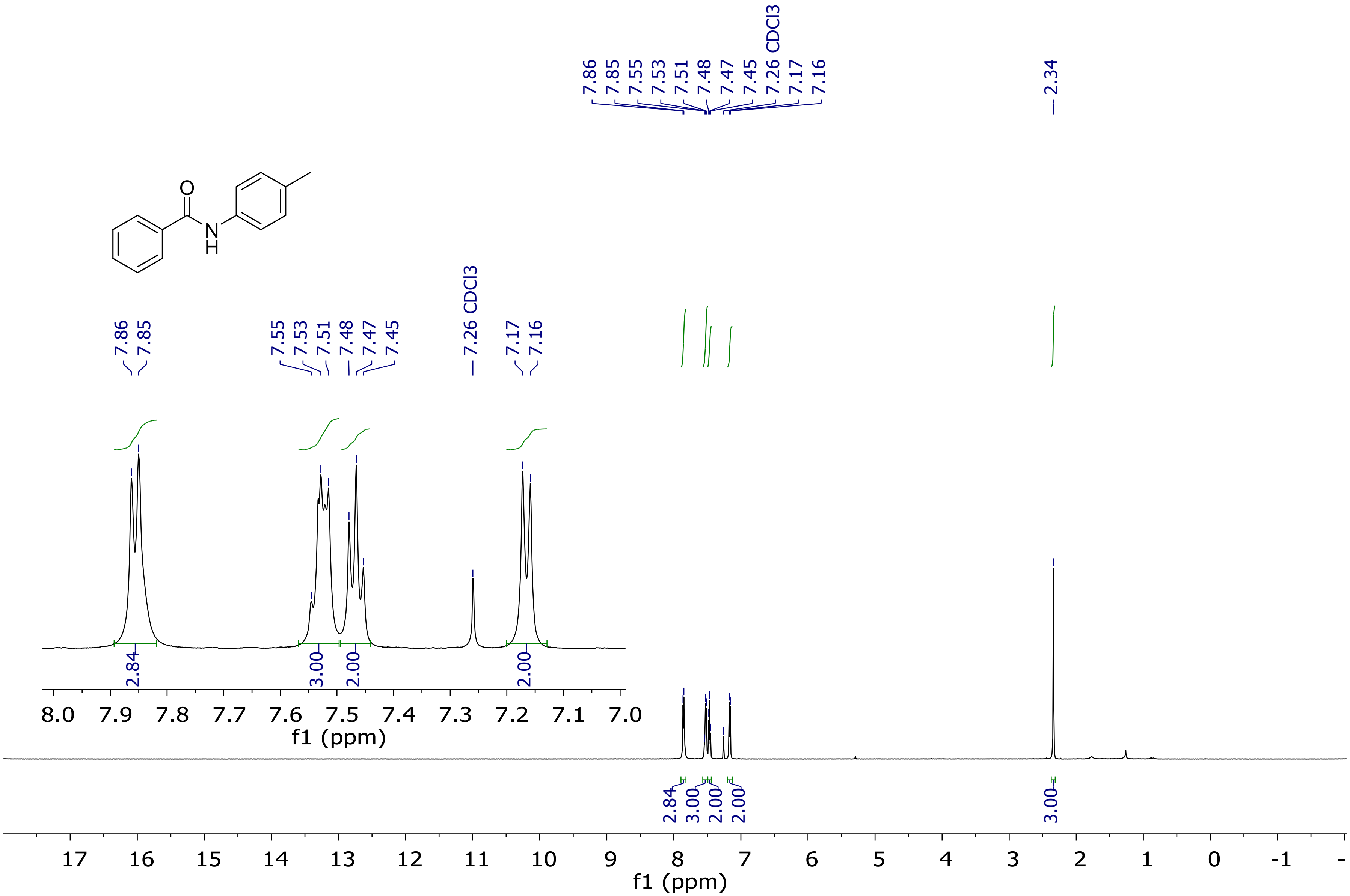

Figure S3. ¹H NMR (600 MHz, Chloroform-*d*) of N-p-tolylbenzamide (3b).

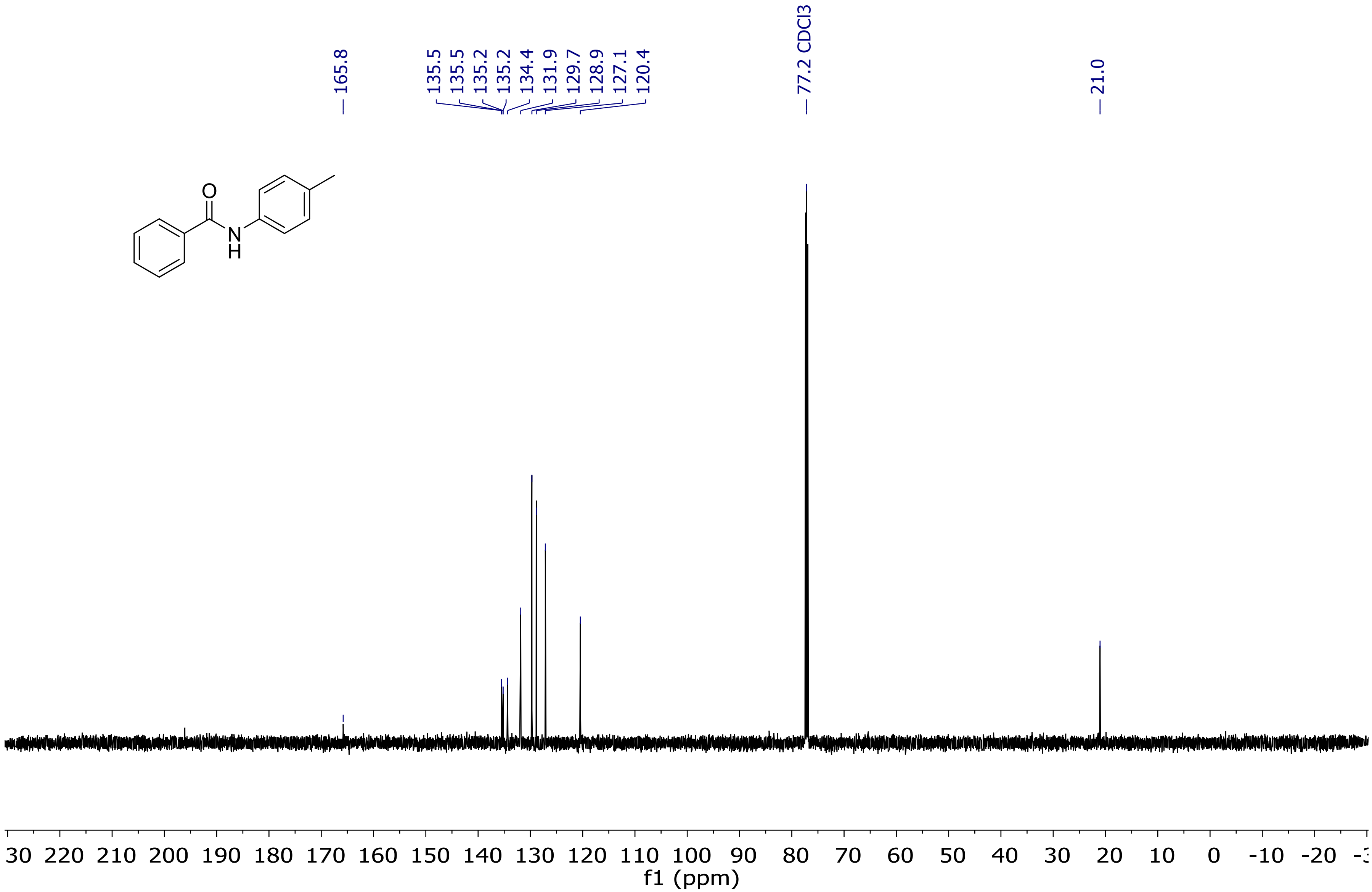

Figure S4. $^{13}C\{^1H\}$ **NMR (151 MHz, Chloroform-d) of N-p-tolylbenzamide (3b).**

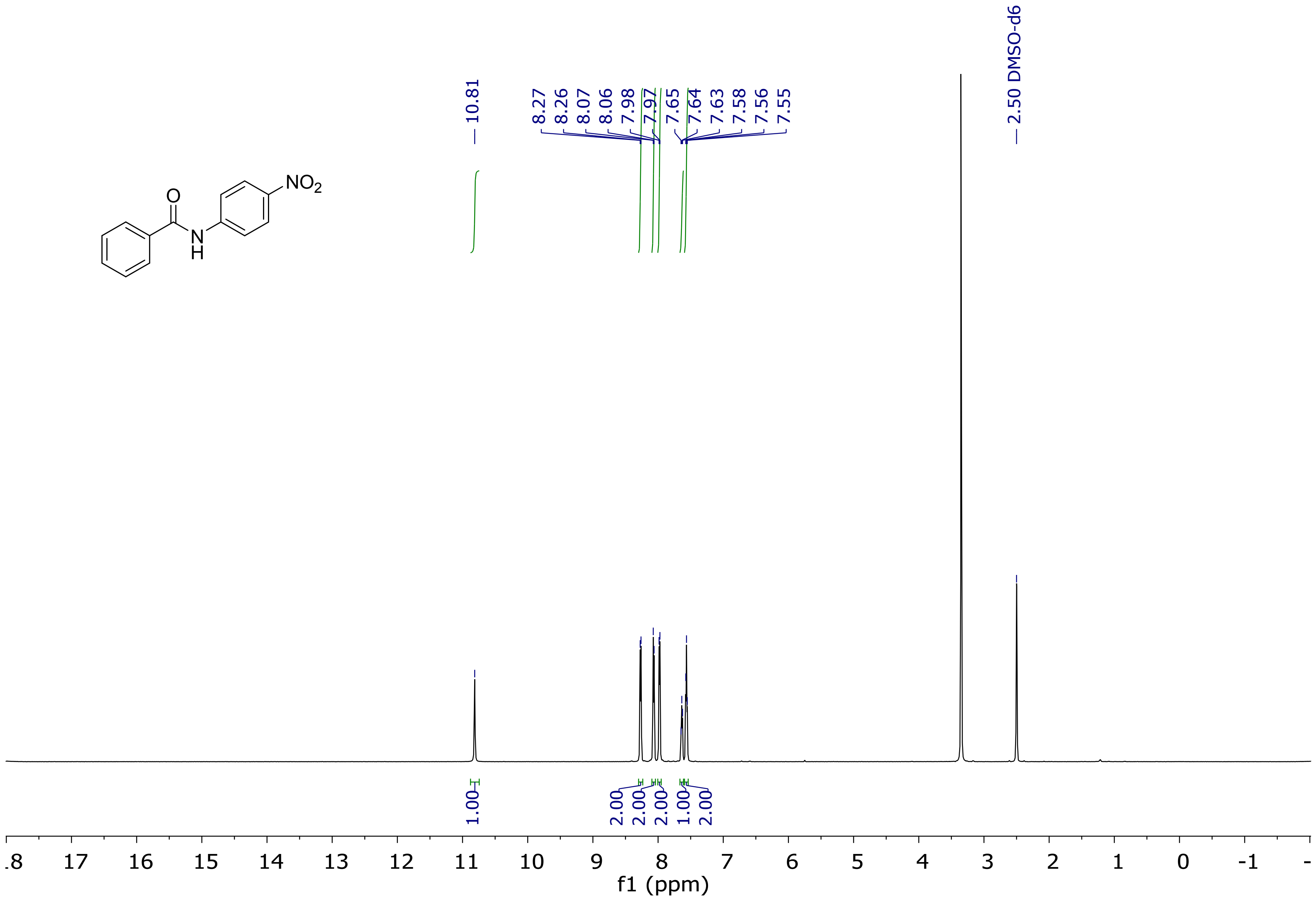

Figure S5. ¹H NMR (600 MHz, Chloroform-*d*) of N-(4-nitrophenyl)benzamide (3c).

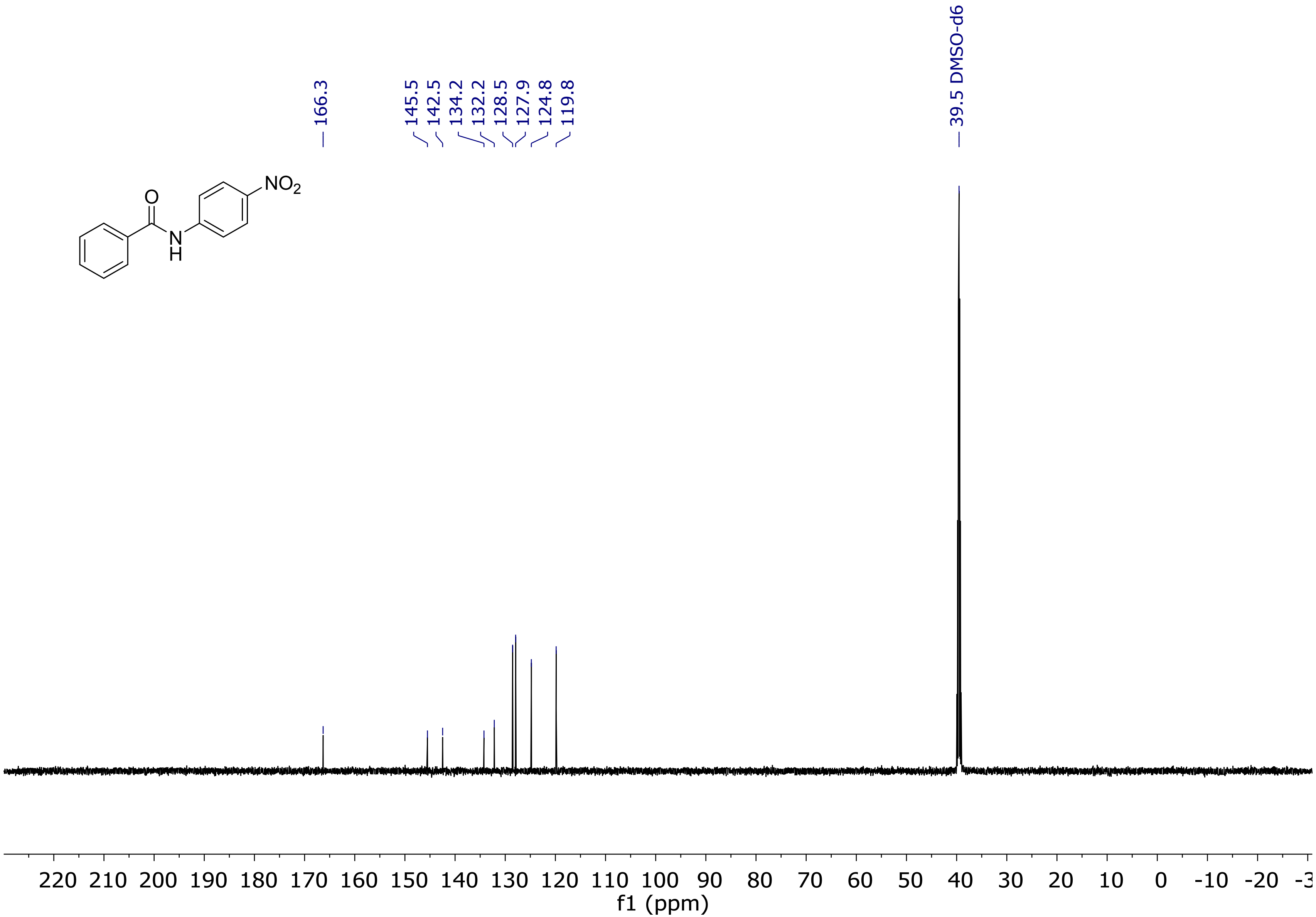

Figure S6. $^{13}C\{^{1}H\}$ NMR (151 MHz, Chloroform-d) of N-(4-nitrophenyl)benzamide (3c).

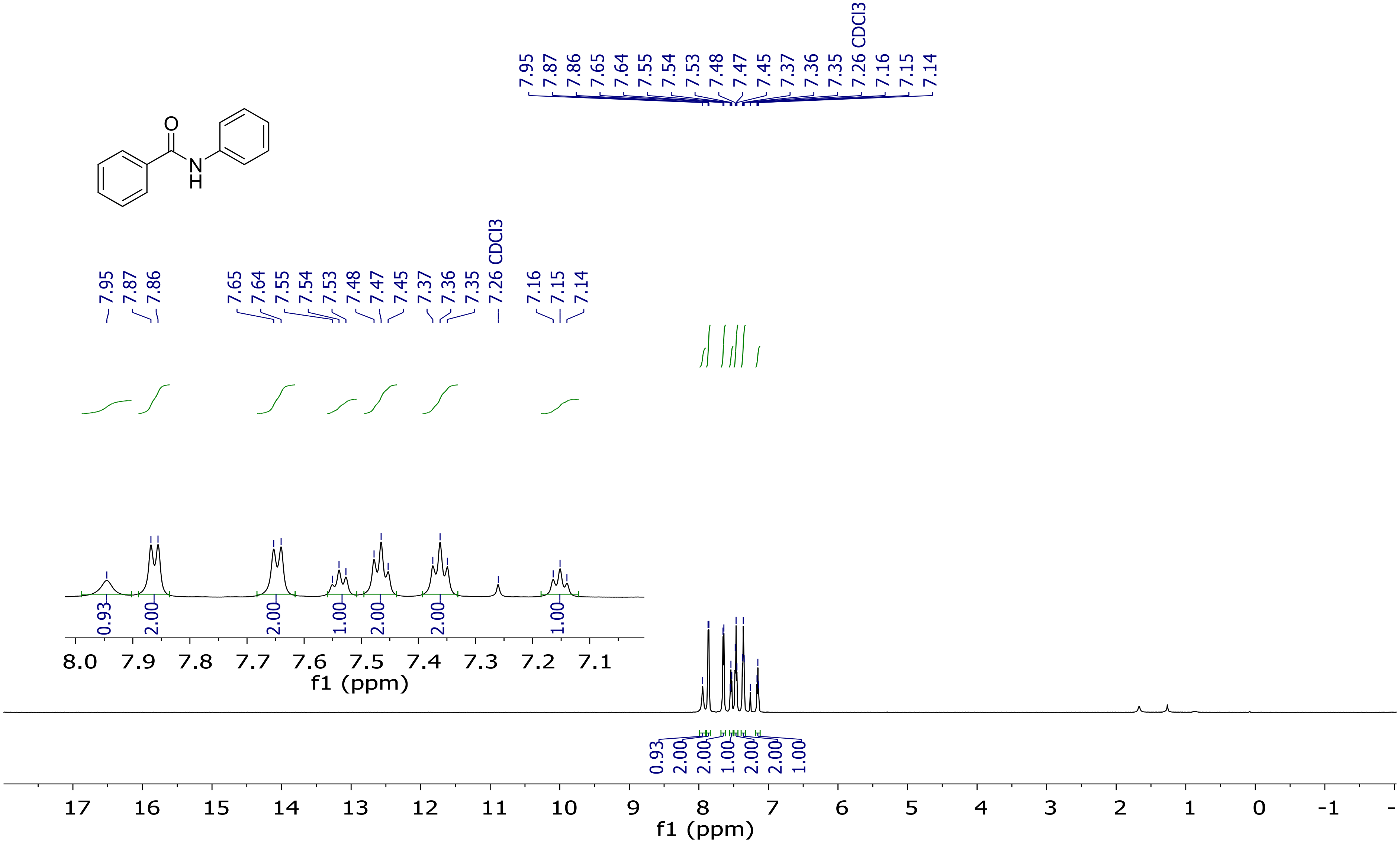

Figure S7. ^{1}**H NMR (600 MHz, Chloroform-*d*) of N-phenylbenzamide (3d).**

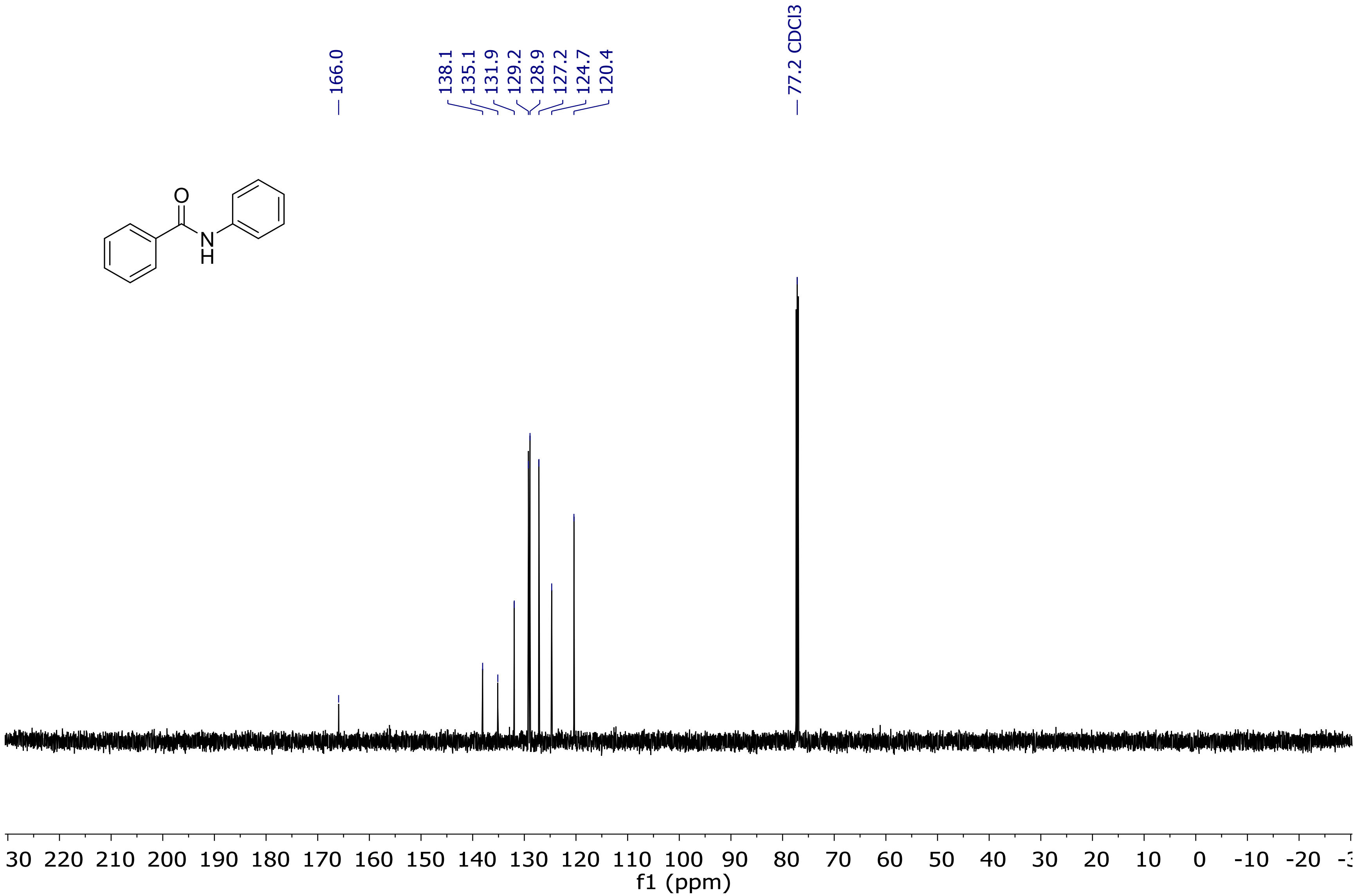

Figure S8. $^{13}C\{^{1}H\}$ NMR (151 MHz, Chloroform-d) of N-phenylbenzamide (3d).

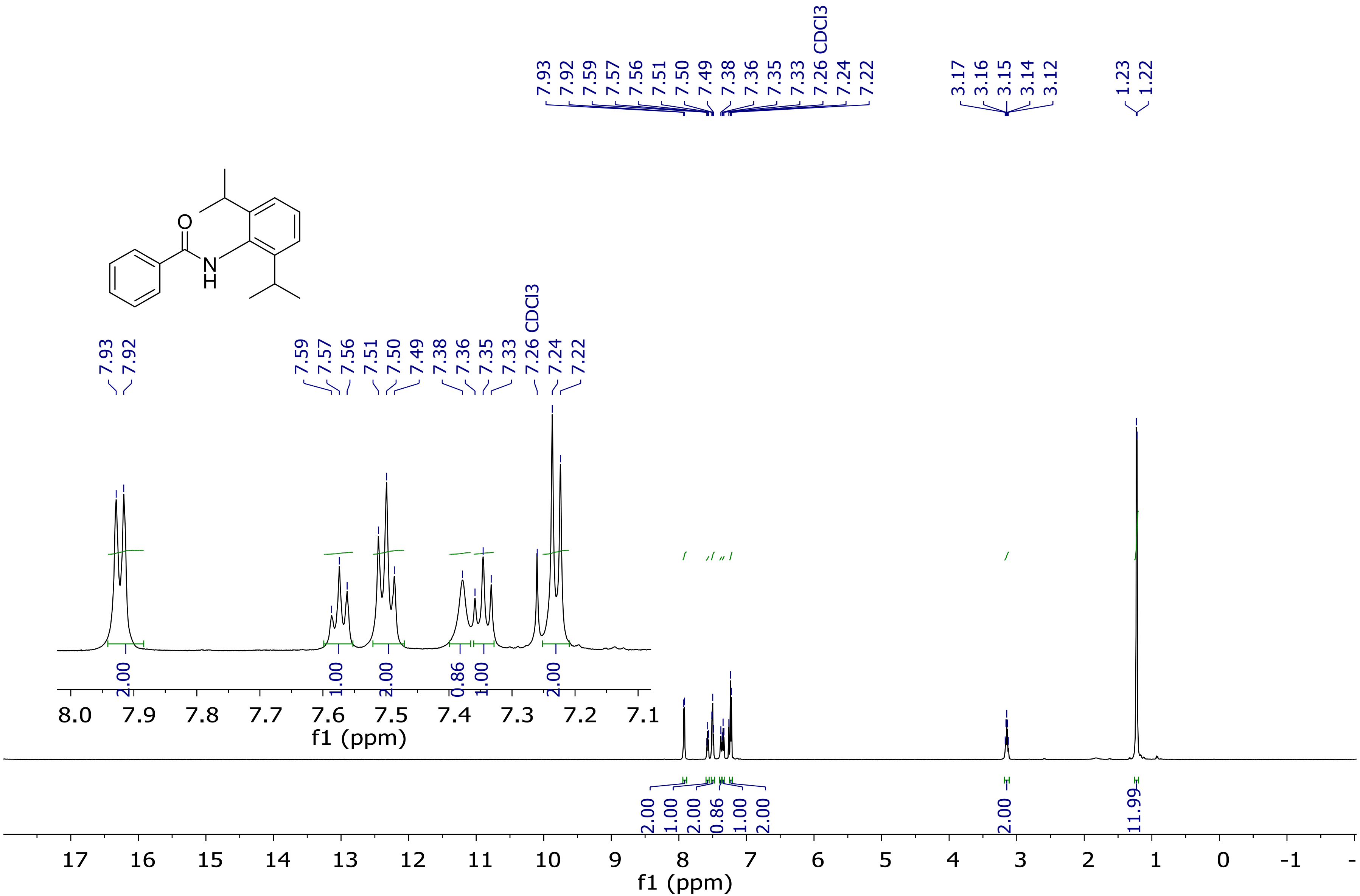

Figure S9. ^{1}**H NMR (600 MHz, Chloroform-*d*) of N-(2,6-diisopropylphenyl)benzamide (3e).**

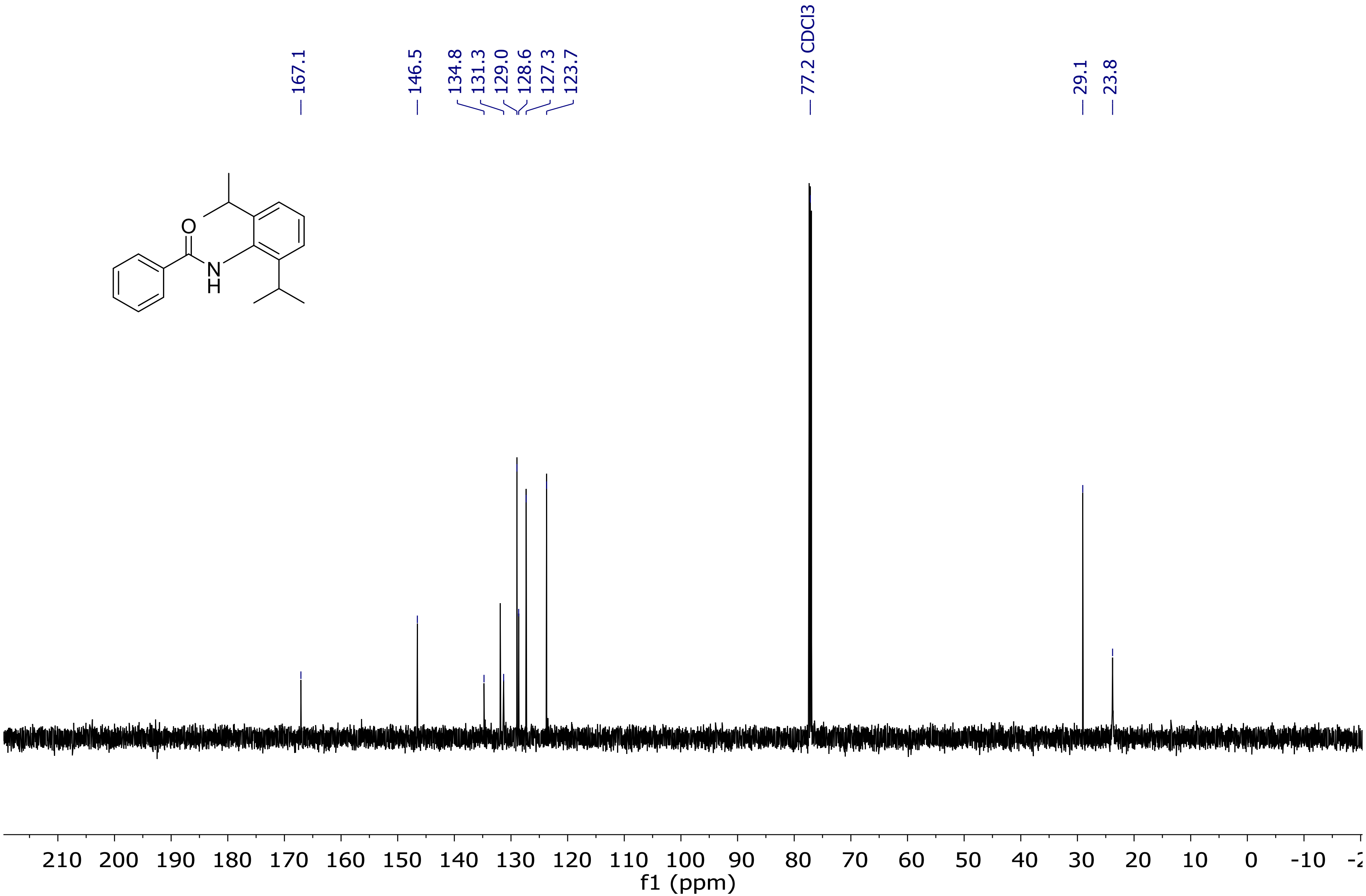

Figure S10. $^{13}C\{^{1}H\}$ **NMR (151 MHz, Chloroform-d) of N-(2,6-diisopropylphenyl)benzamide (3e).**

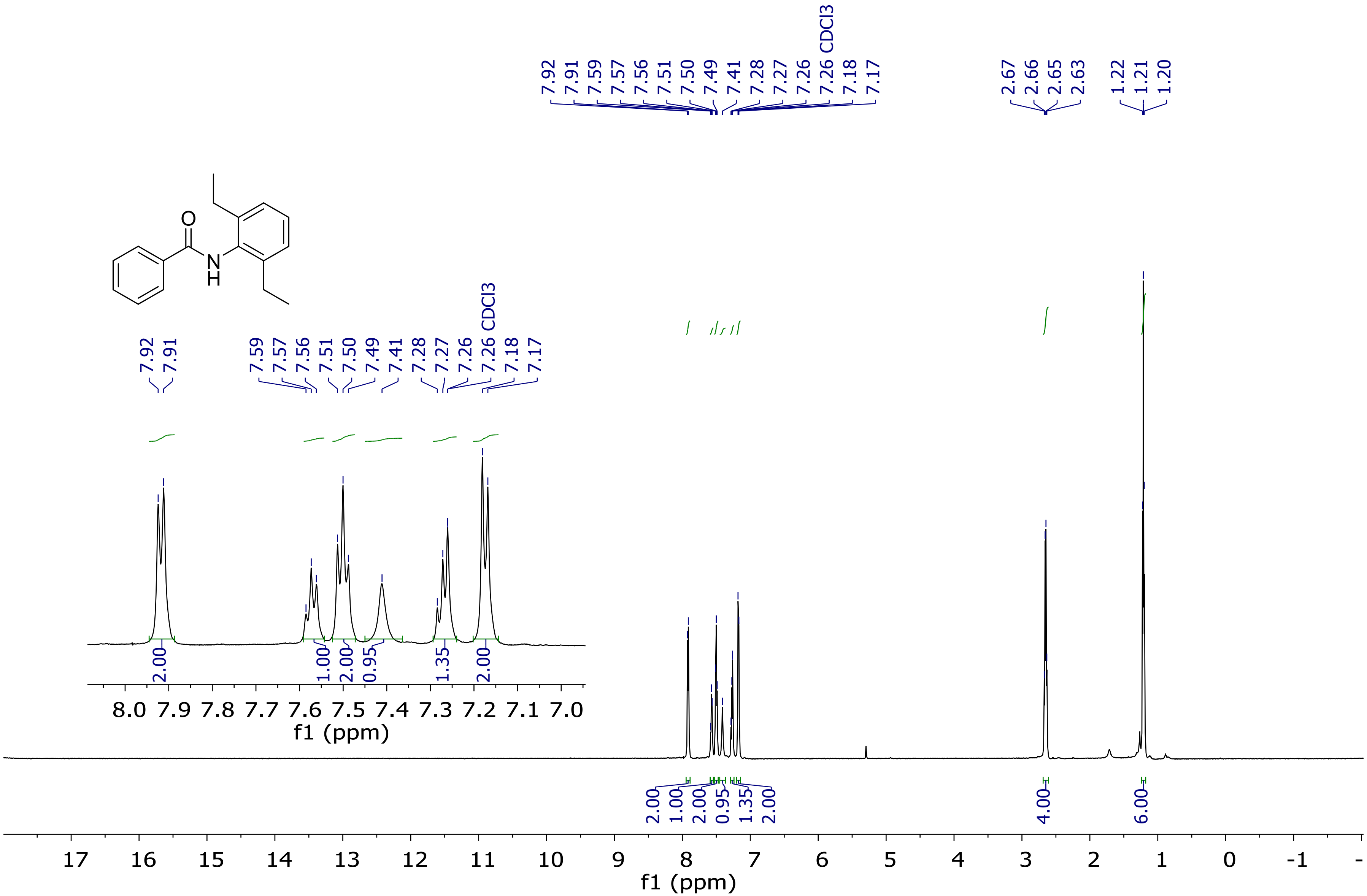

Figure S11. ^{1}**H NMR (600 MHz, Chloroform-*d*) of N-(2,6-diethylphenyl)benzamide (3f).**

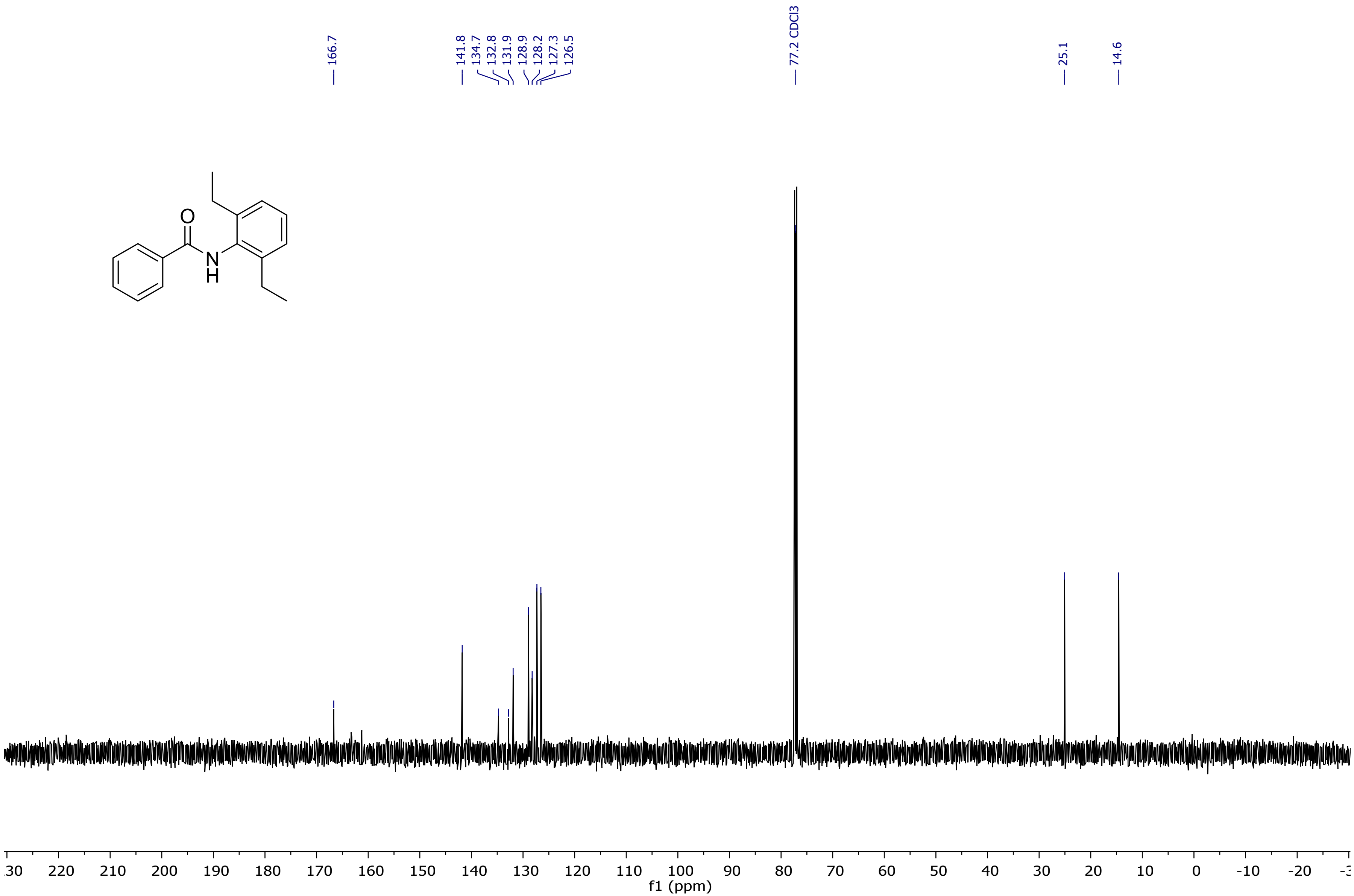

Figure S12. $^{13}C\{^{1}H\}$ **NMR (151 MHz, Chloroform-d) of N-(2,6-diethylphenyl)benzamide (3f).**

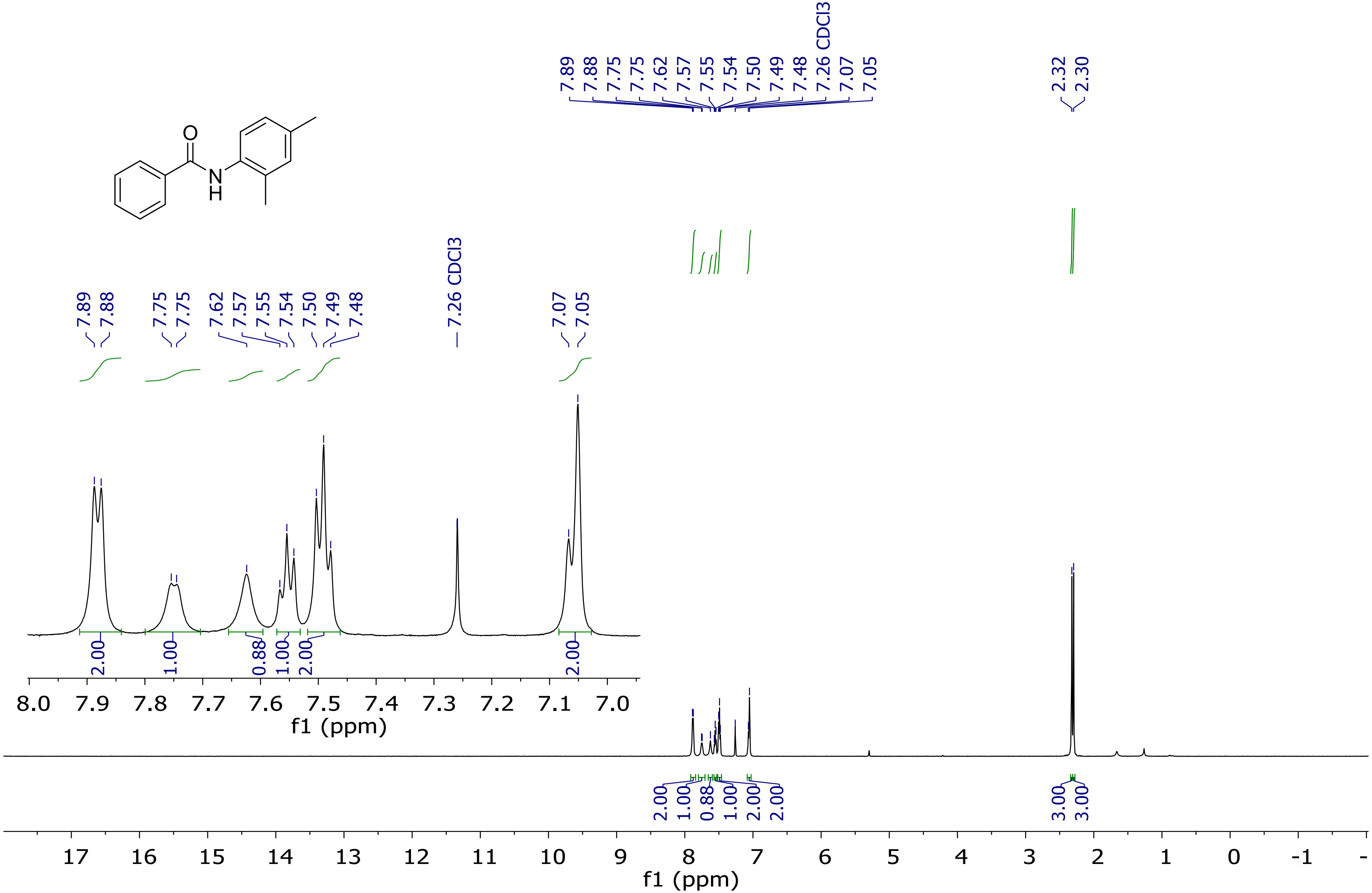

Figure S13. ^{1}**H NMR (600 MHz, Chloroform-*d*) of N-(2,4-dimethylphenyl)benzamide (3g).**

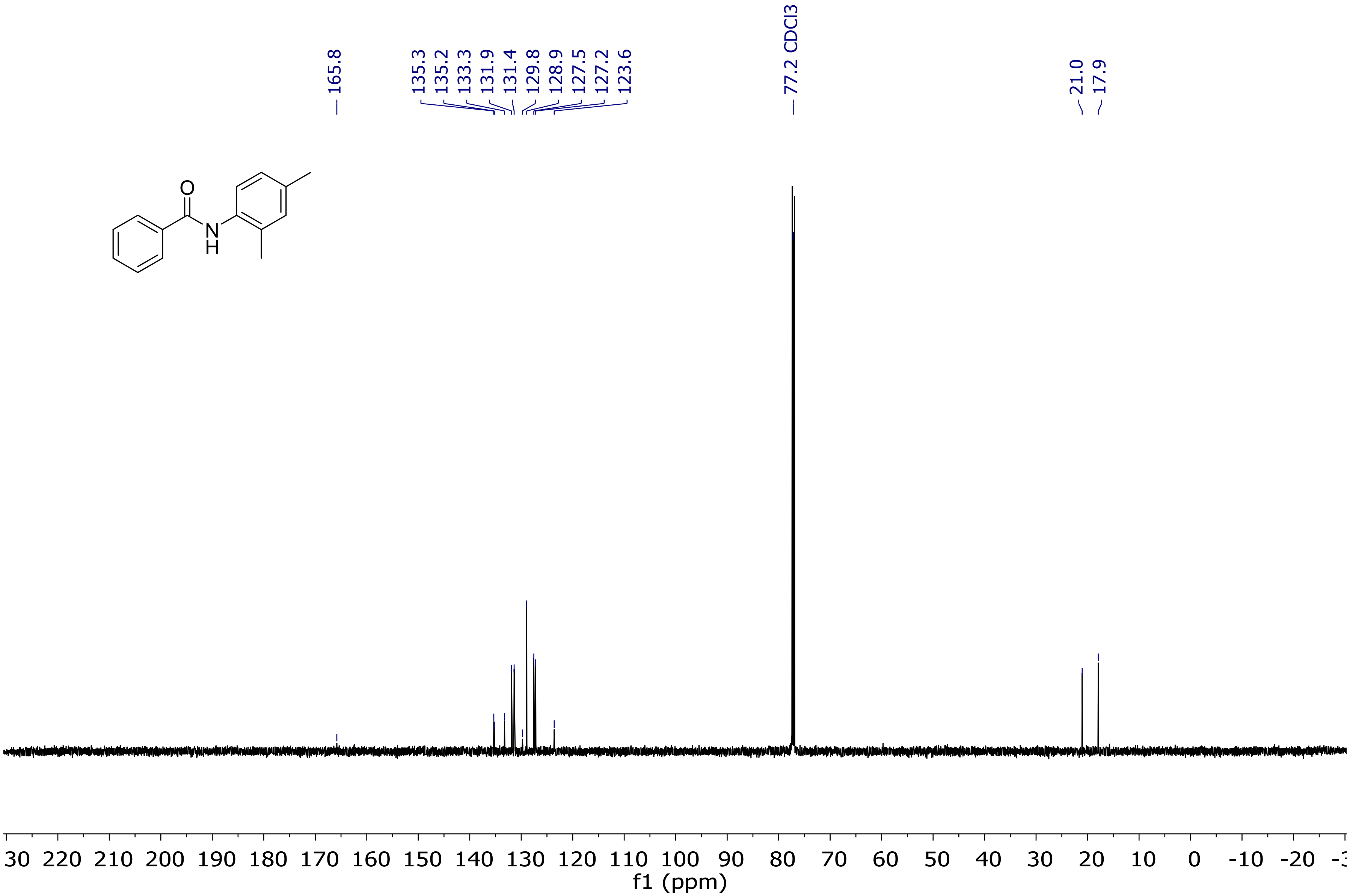

Figure S14. $^{13}C\{^{1}H\}$ NMR (151 MHz, Chloroform-d) of N-(2,4-dimethylphenyl)benzamide (3g).

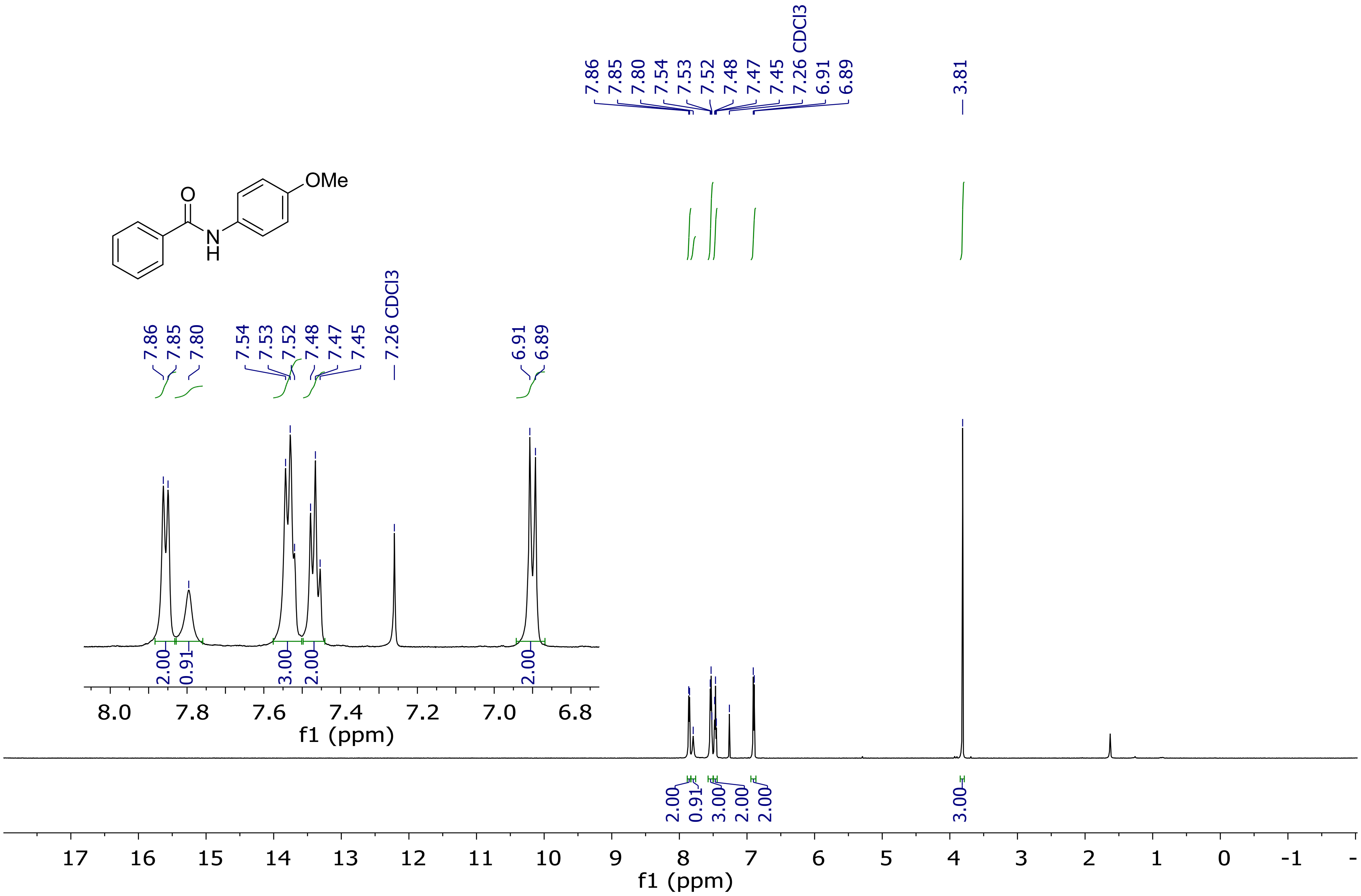

Figure S15. **^{1}H NMR (600 MHz, Chloroform-*d*) of N-(4-methoxyphenyl)benzamide (3h).**

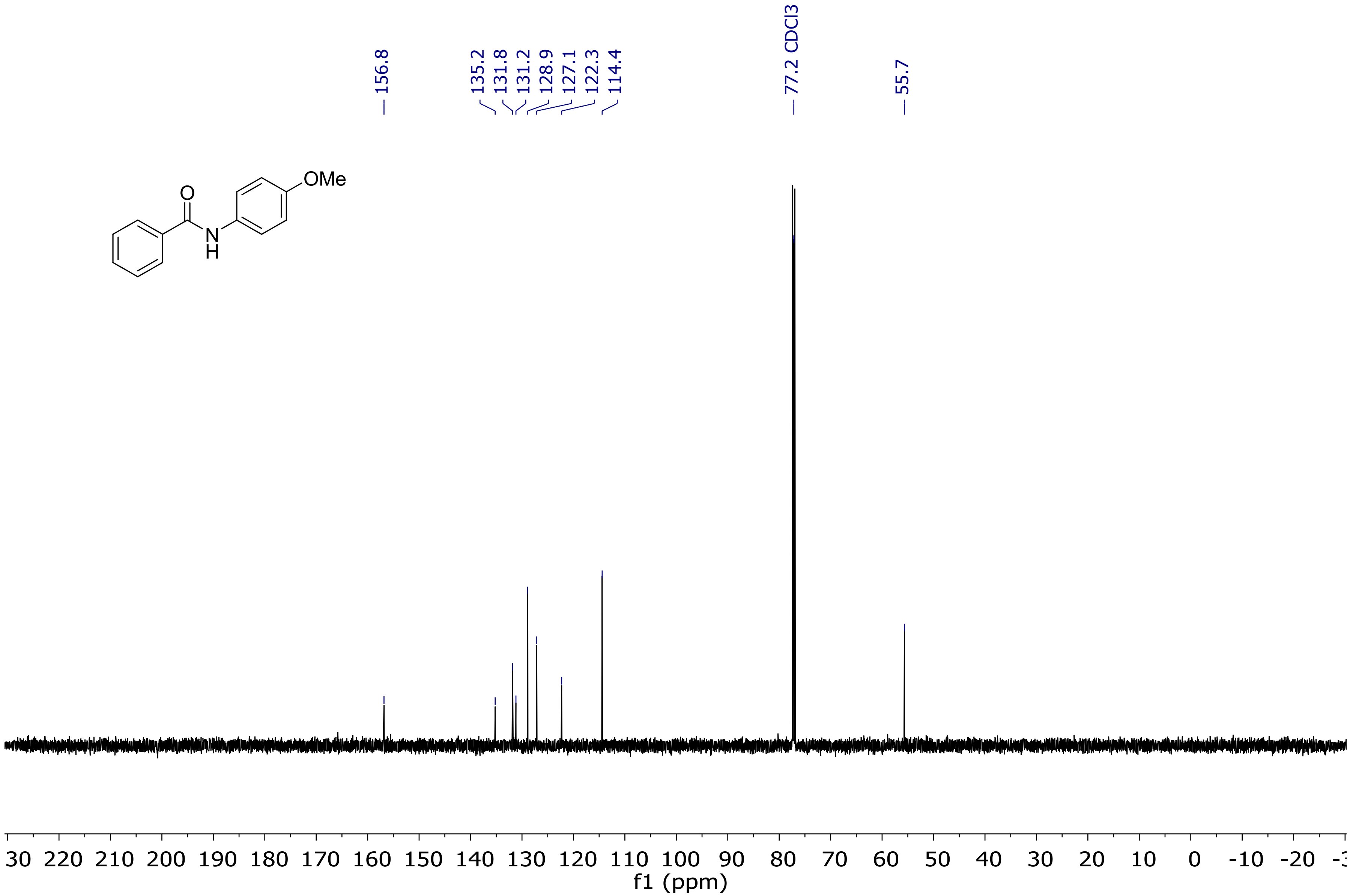

Figure S16. $^{13}C\{^1H\}$ NMR (151 MHz, Chloroform-d) of N-(4-methoxyphenyl)benzamide (3h).

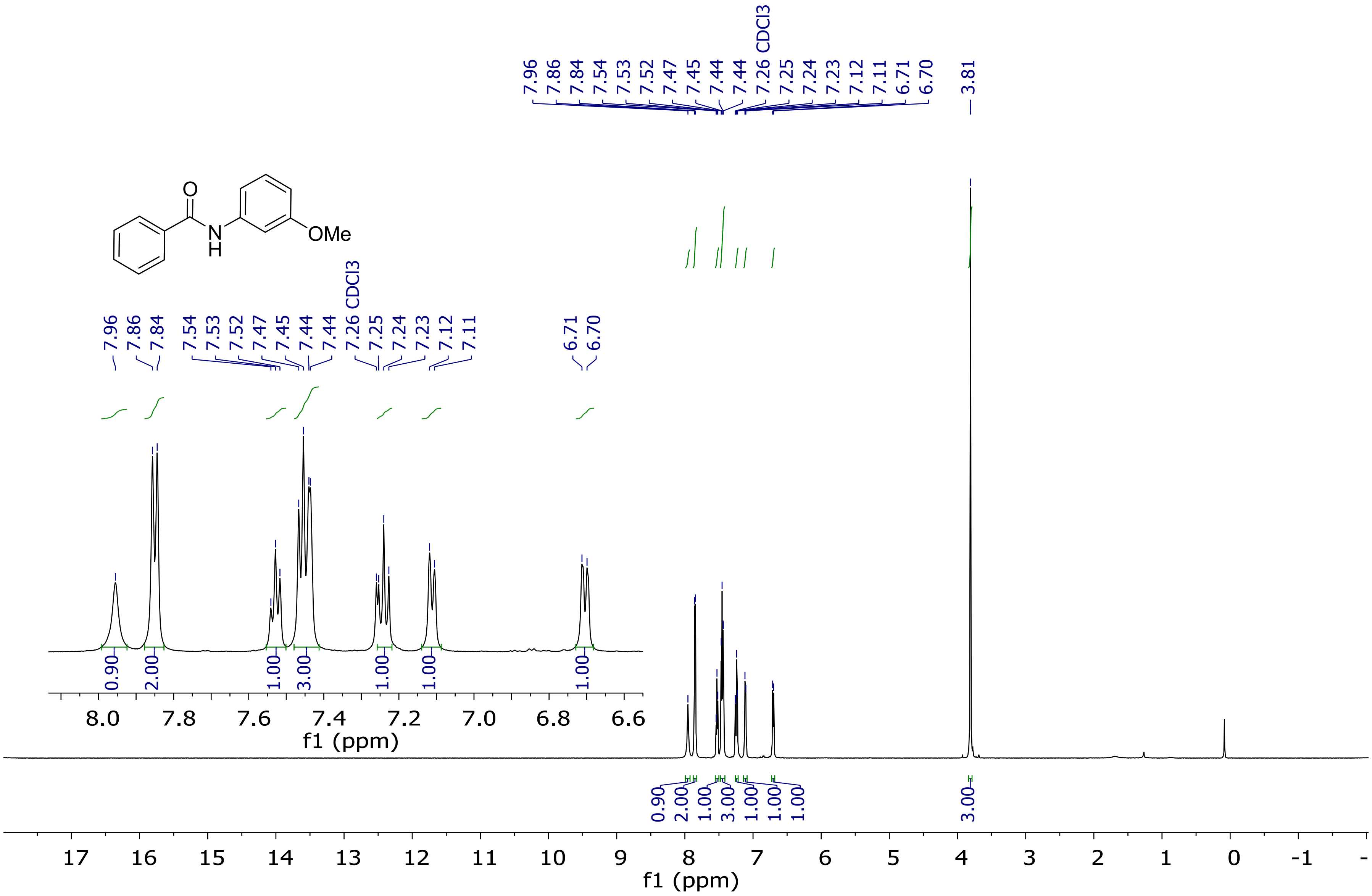

Figure S17. **^{1}H NMR (600 MHz, Chloroform-*d*) of N-(3-methoxyphenyl)benzamide (3i).**

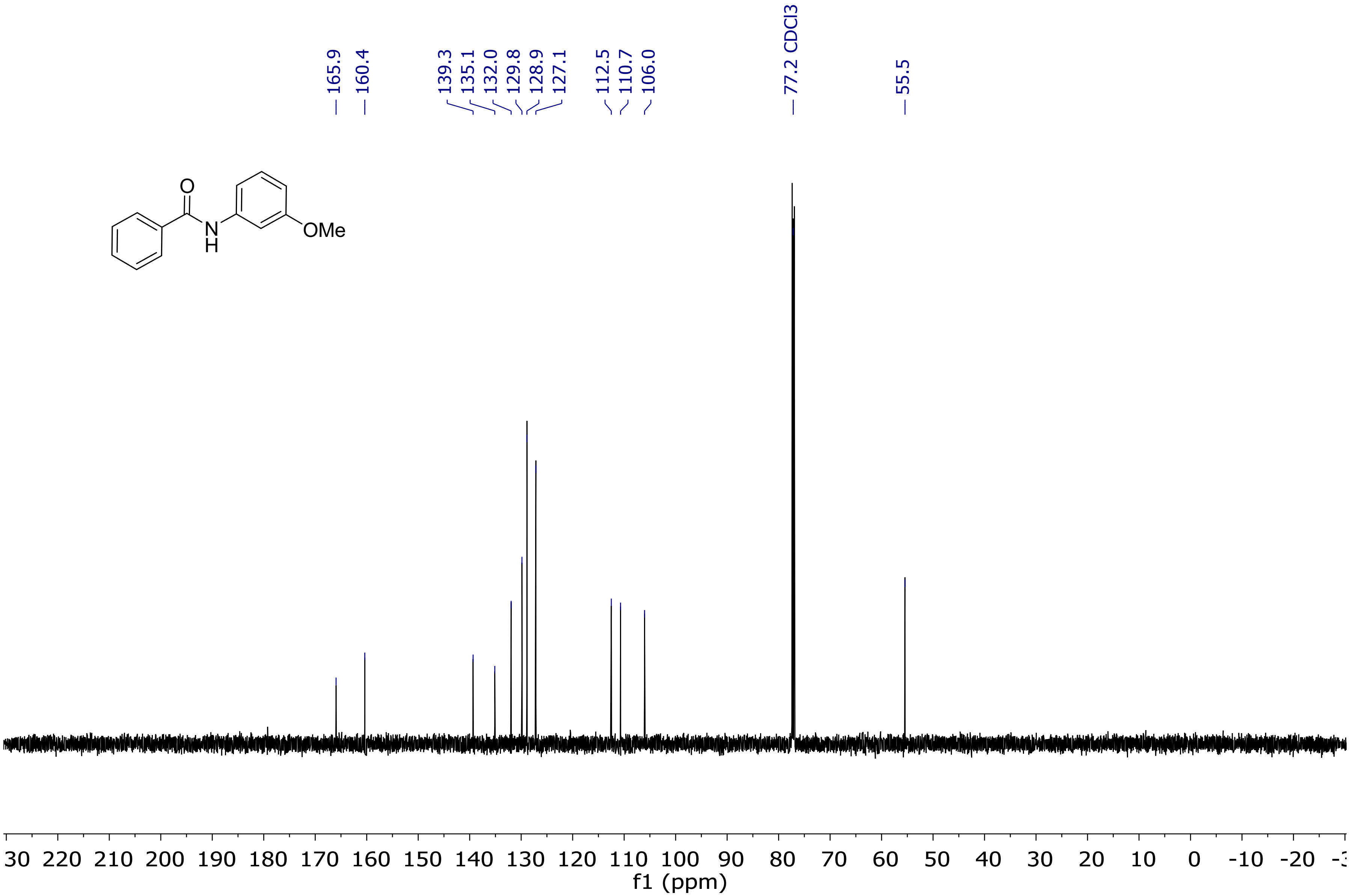

Figure S18. $^{13}C\{^{1}H\}$ **NMR (151 MHz, Chloroform-d) of N-(3-methoxyphenyl)benzamide (3i).**

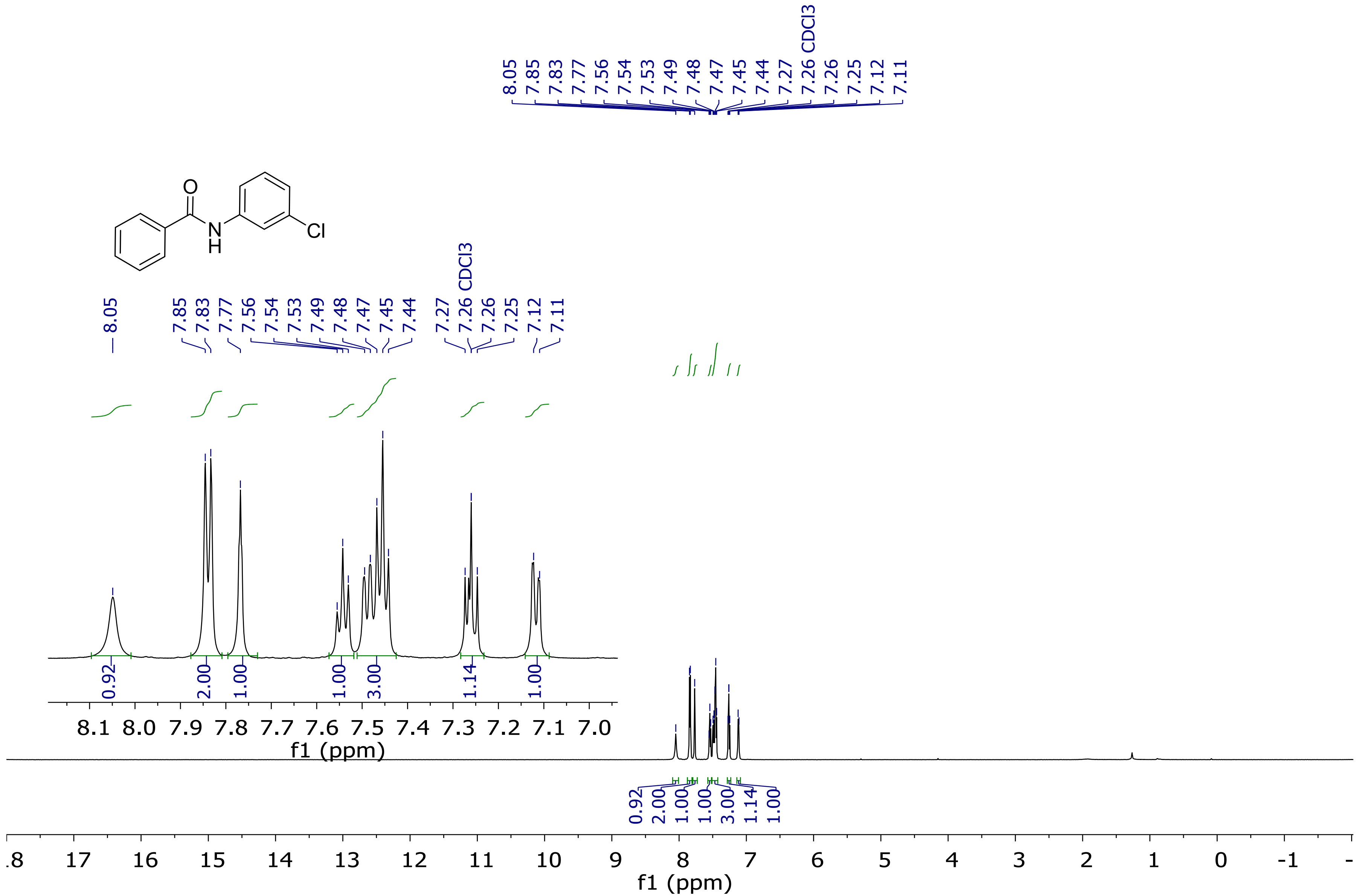

Figure S19. ¹H NMR (600 MHz, Chloroform-*d*) of N-(3-chlorophenyl)benzamide (3j).

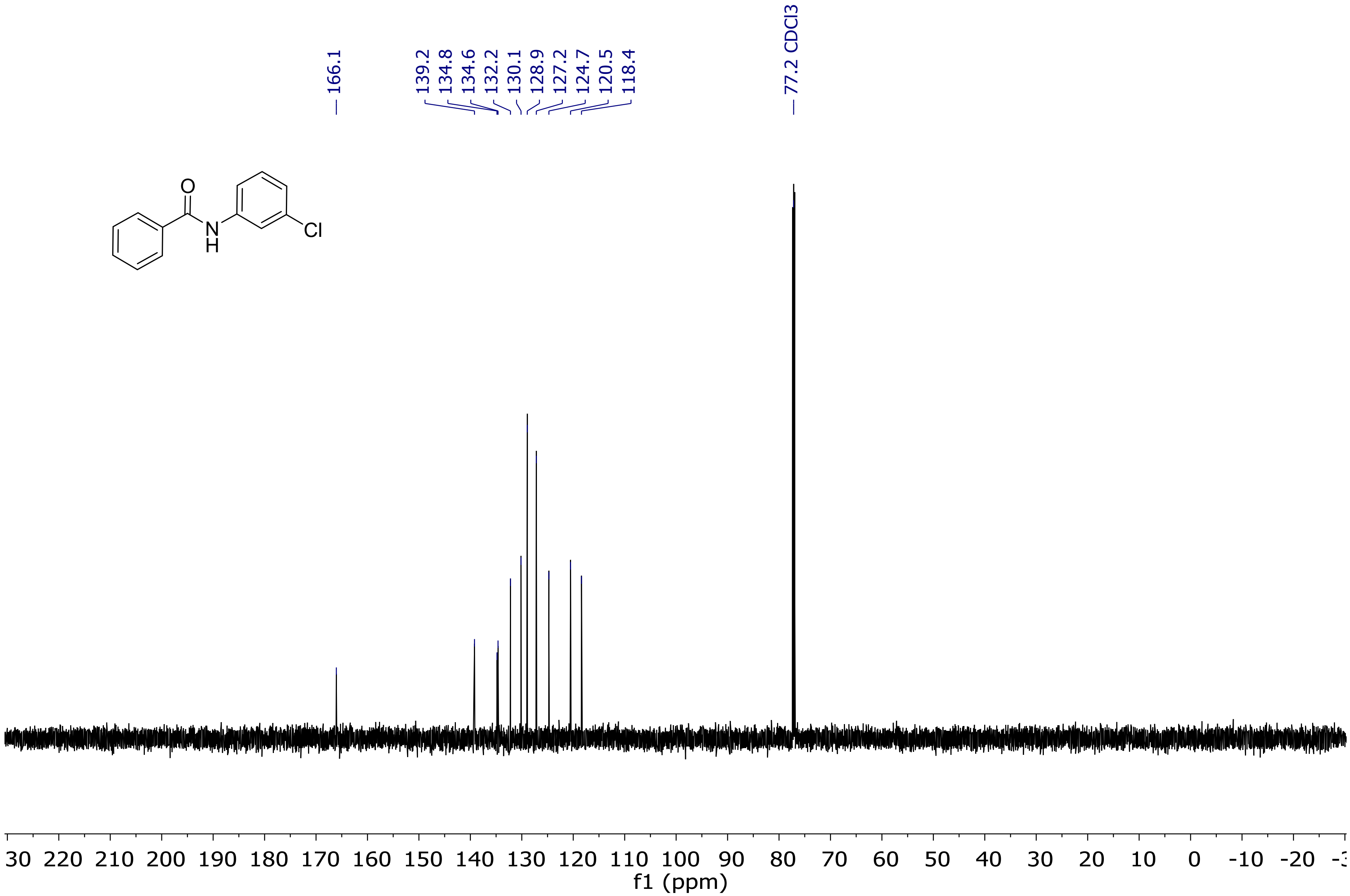

Figure S20. $^{13}C\{^1H\}$ **NMR (151 MHz, Chloroform-d) of N-(3-chlorophenyl)benzamide (3j).**

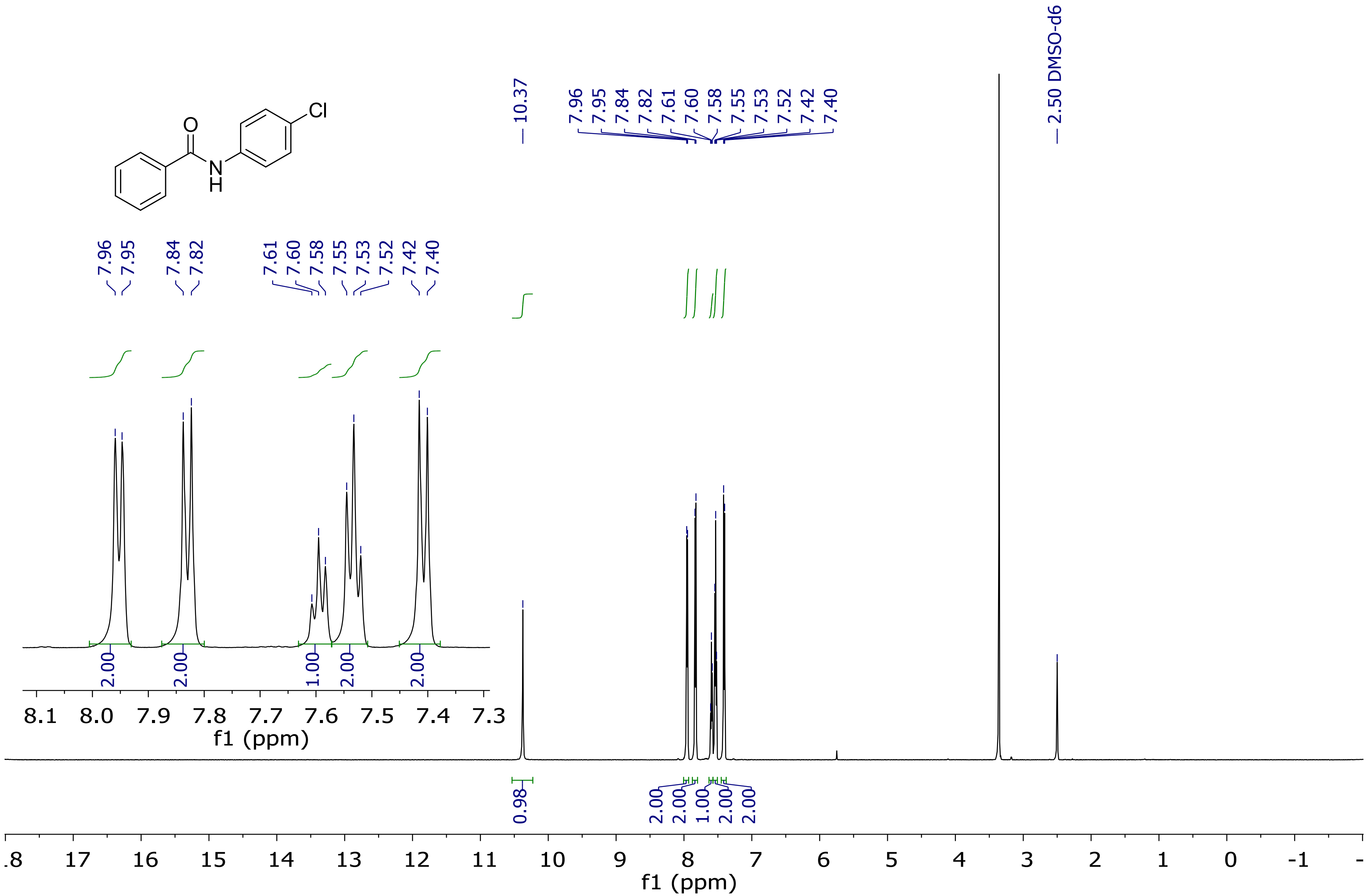

Figure S21. ^{1}H NMR (600 MHz, Chloroform-*d*) of N-(4-chlorophenyl)benzamide (3k).

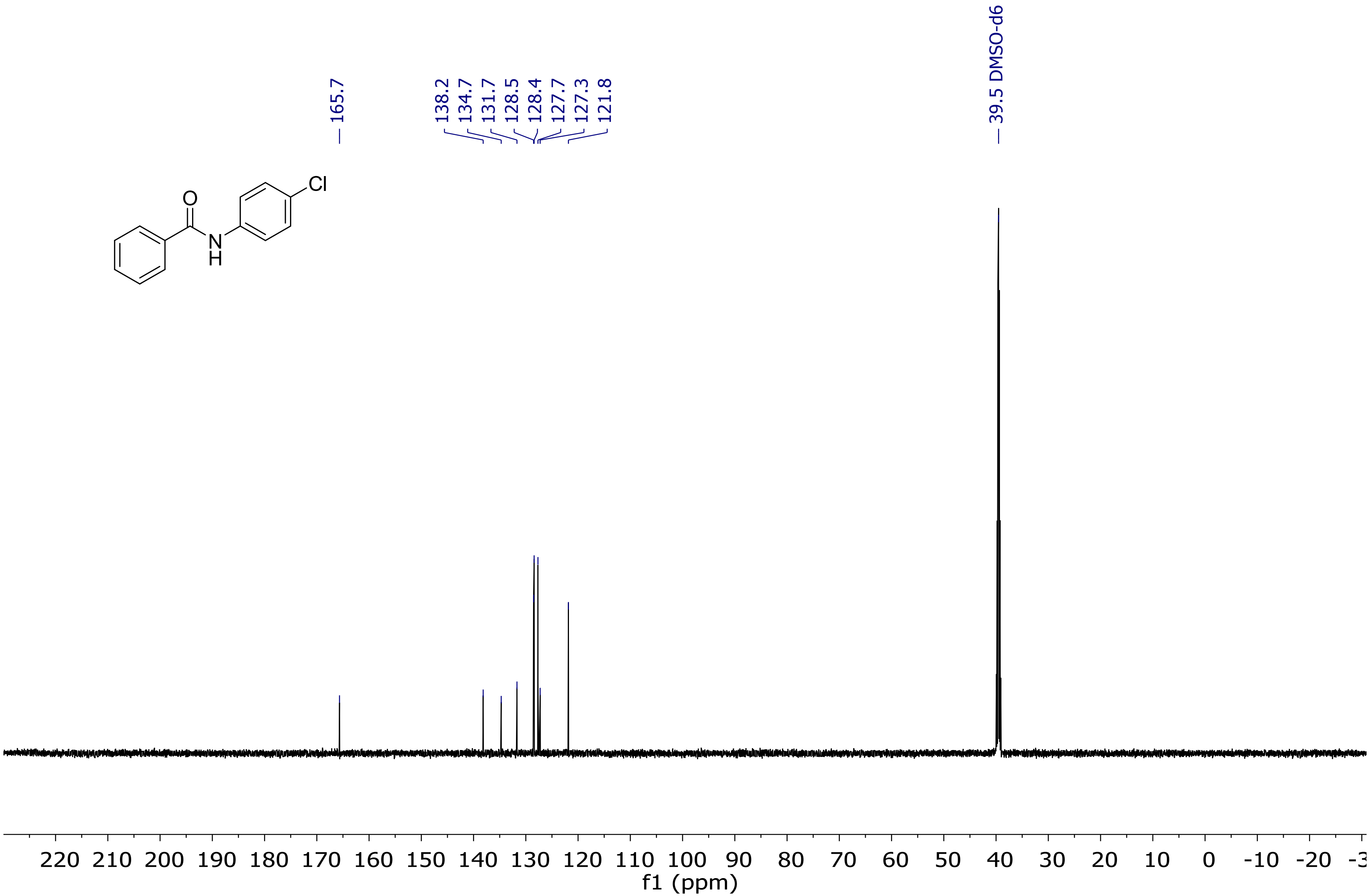

Figure S22. $^{13}C\{^{1}H\}$ **NMR (151 MHz, Chloroform-d) of N-(4-chlorophenyl)benzamide (3k).**

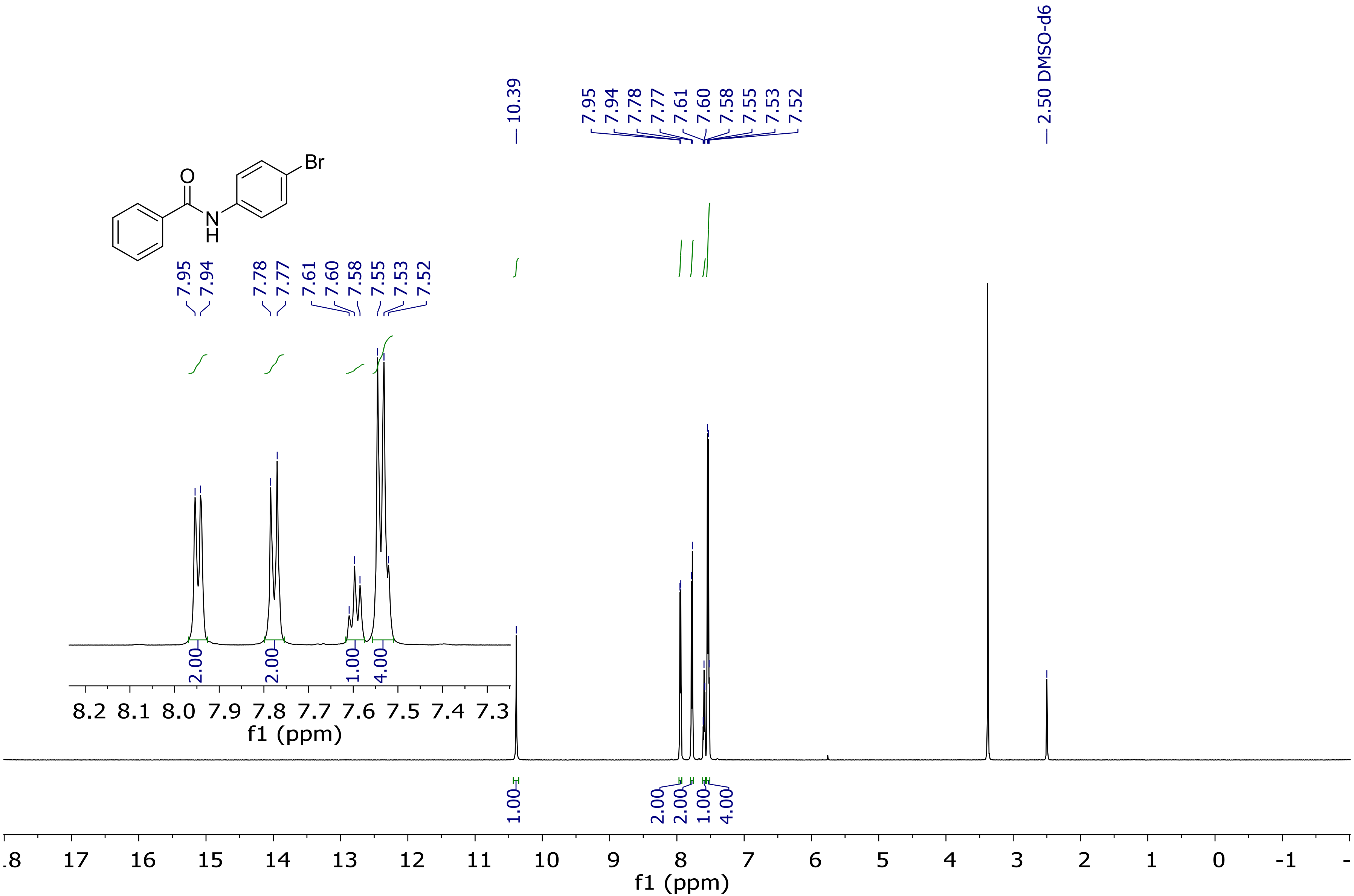

Figure S23. ^{1}H **NMR (600 MHz, DMSO-*d6*) of N-(4-bromophenyl)benzamide (3l).**

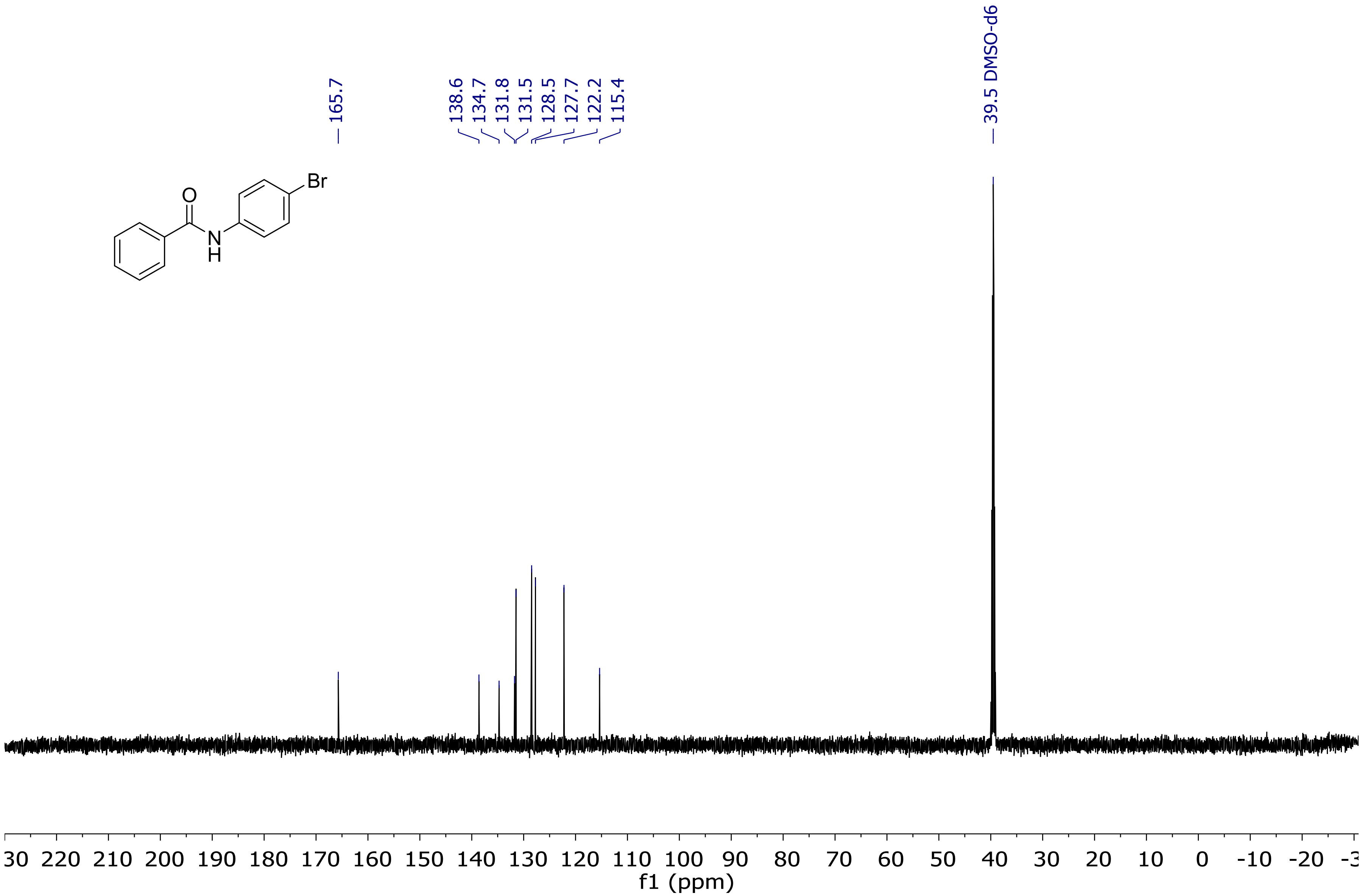

Figure S24. $^{13}C\{^{1}H\}$ **NMR (151 MHz, DMSO-*d6*) of N-(4-bromophenyl)benzamide (3l).**

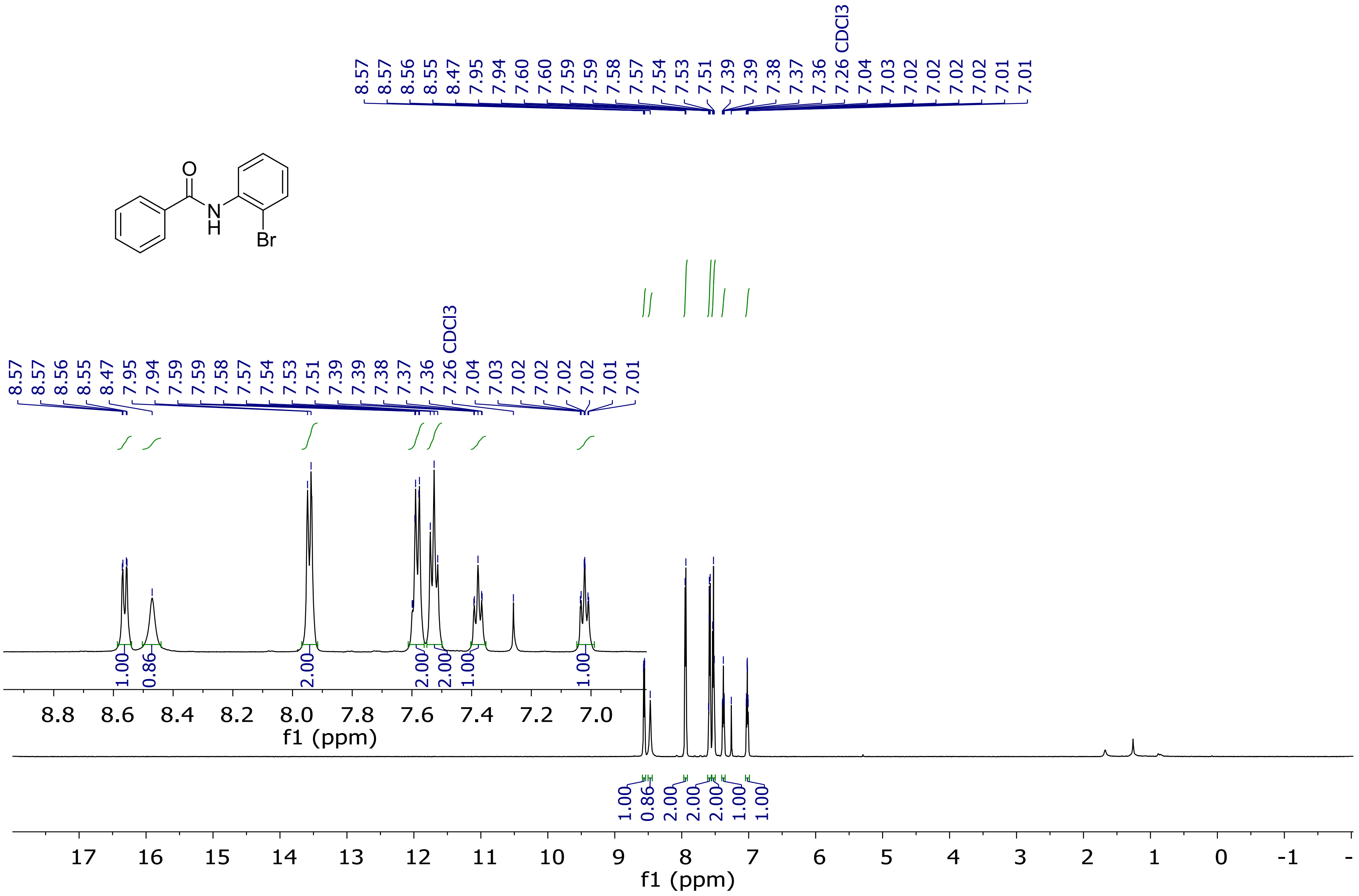

Figure S25. ^{1}H NMR (600 MHz, Chloroform-*d*) of N-(2-bromophenyl)benzamide (3m).

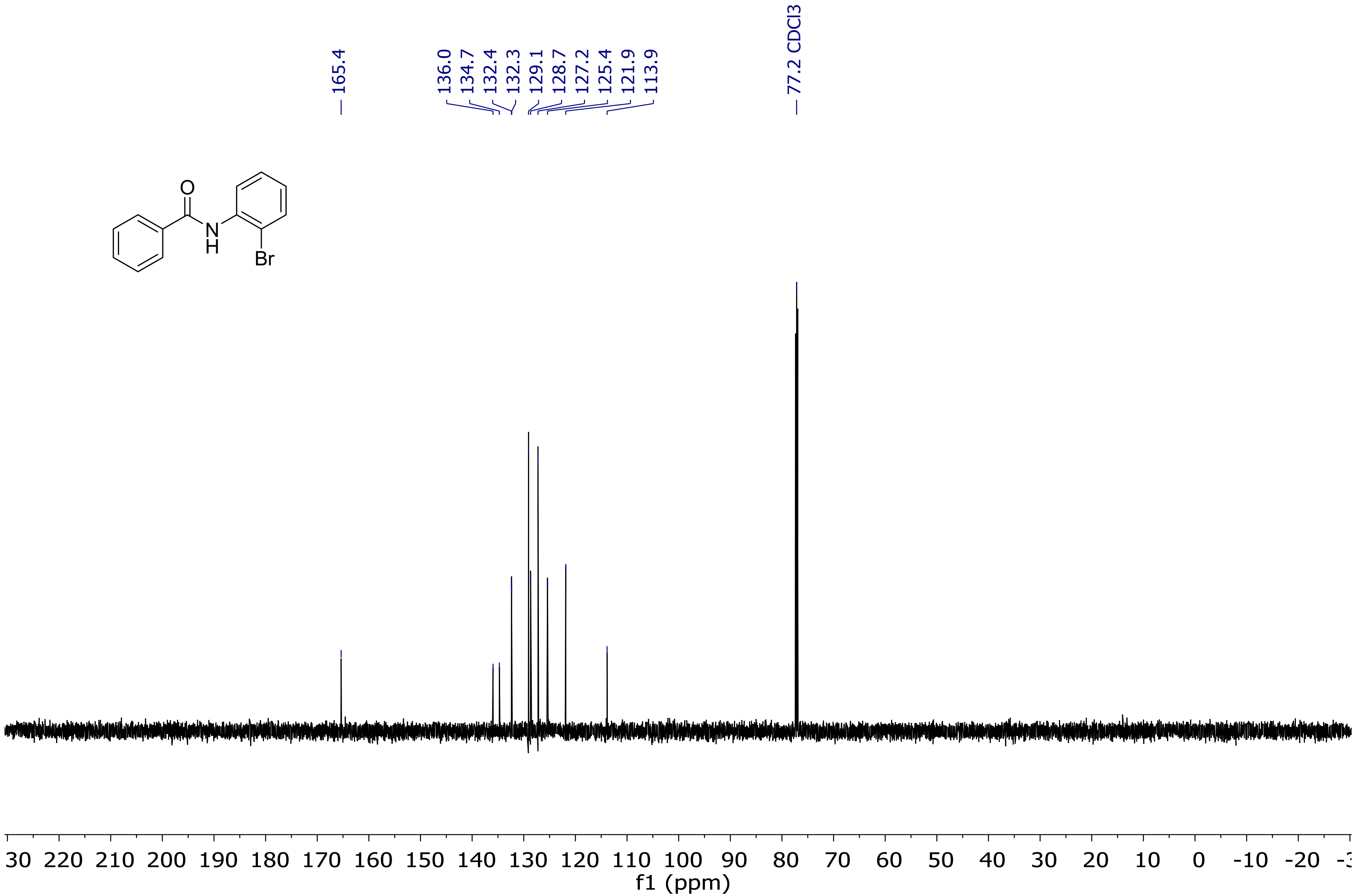

Figure S26. $^{13}C\{^1H\}$ NMR (151 MHz, Chloroform-d) of N-(2-bromophenyl)benzamide (3m).

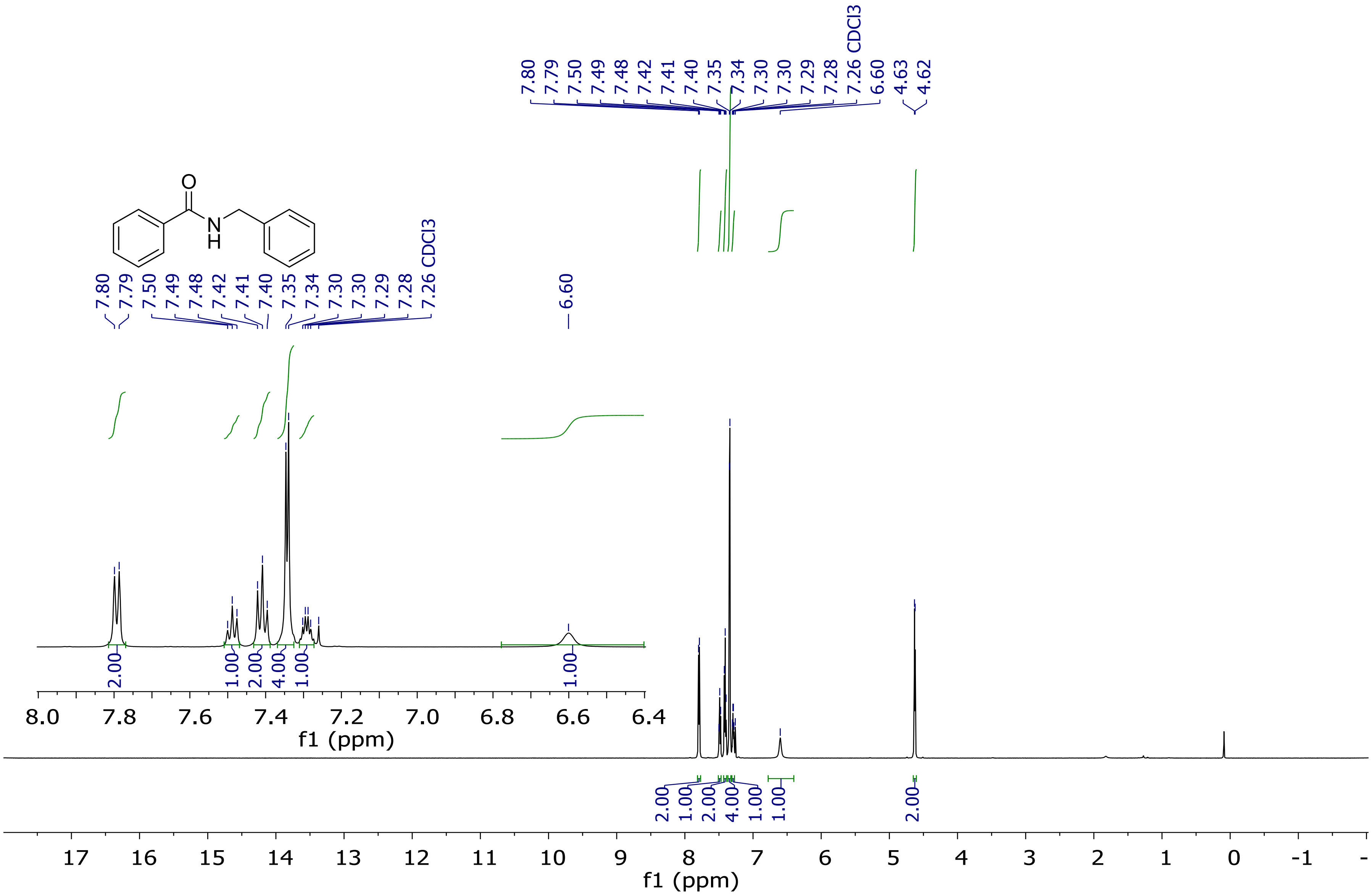

Figure S27. **¹H NMR (600 MHz, Chloroform-*d*) of N-benzylbenzamide (3n).**

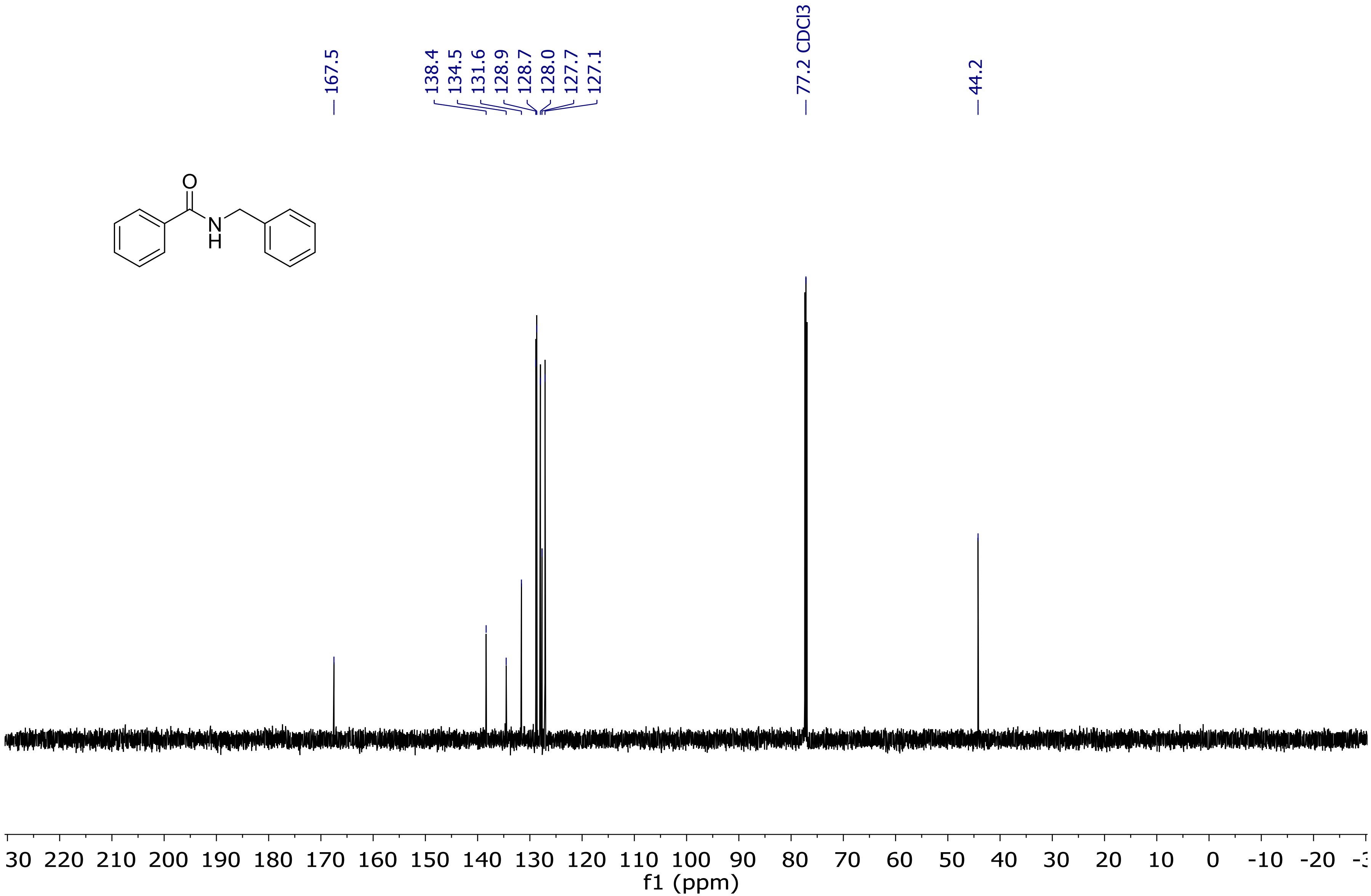

Figure S28. $^{13}C\{^1H\}$ **NMR (151 MHz, Chloroform-d) of N-benzylbenzamide (3n).**

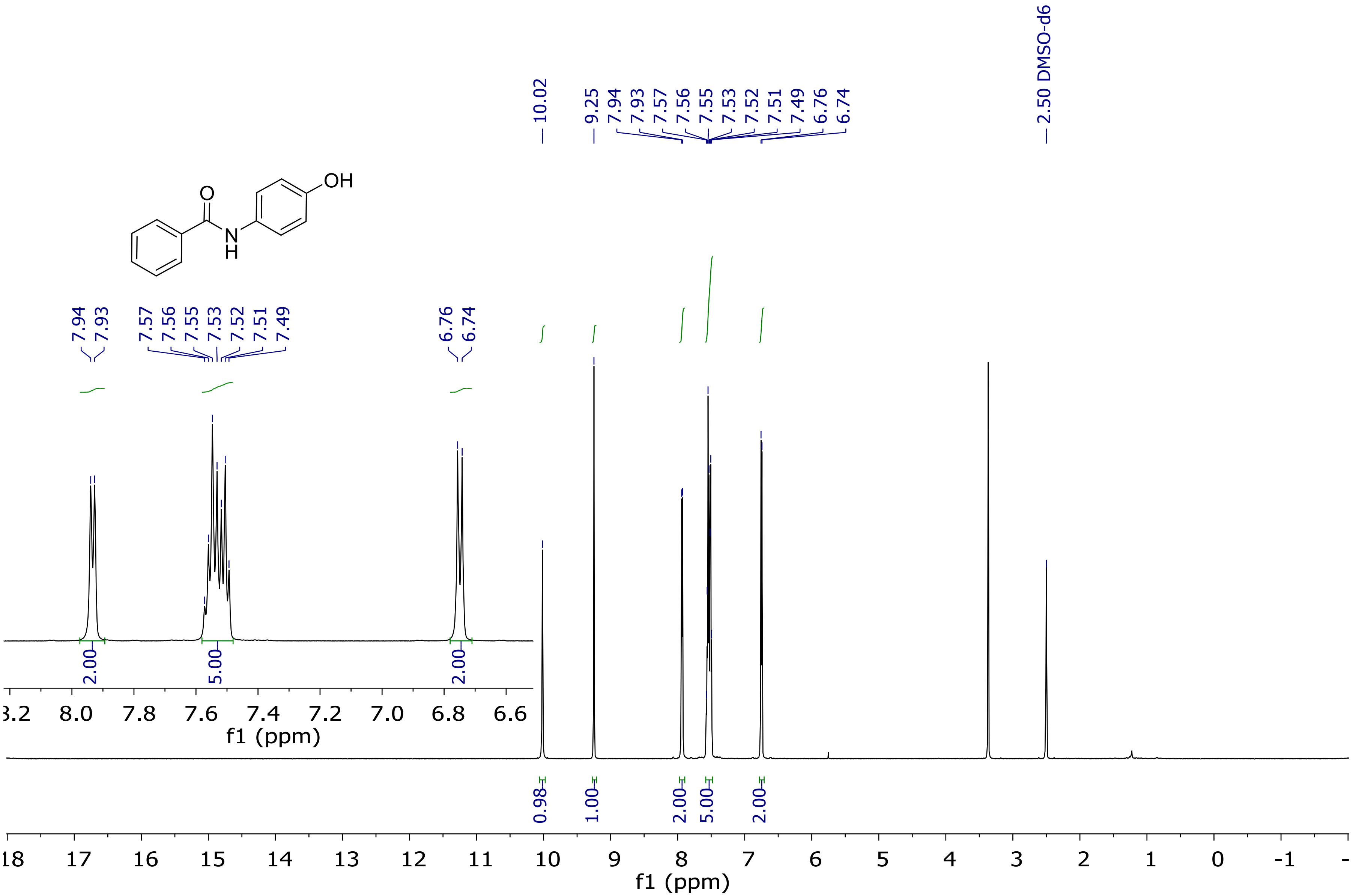

Figure S29. ^{1}H NMR (600 MHz, Chloroform-*d*) of N-(4-hydroxyphenyl)benzamide (3o).

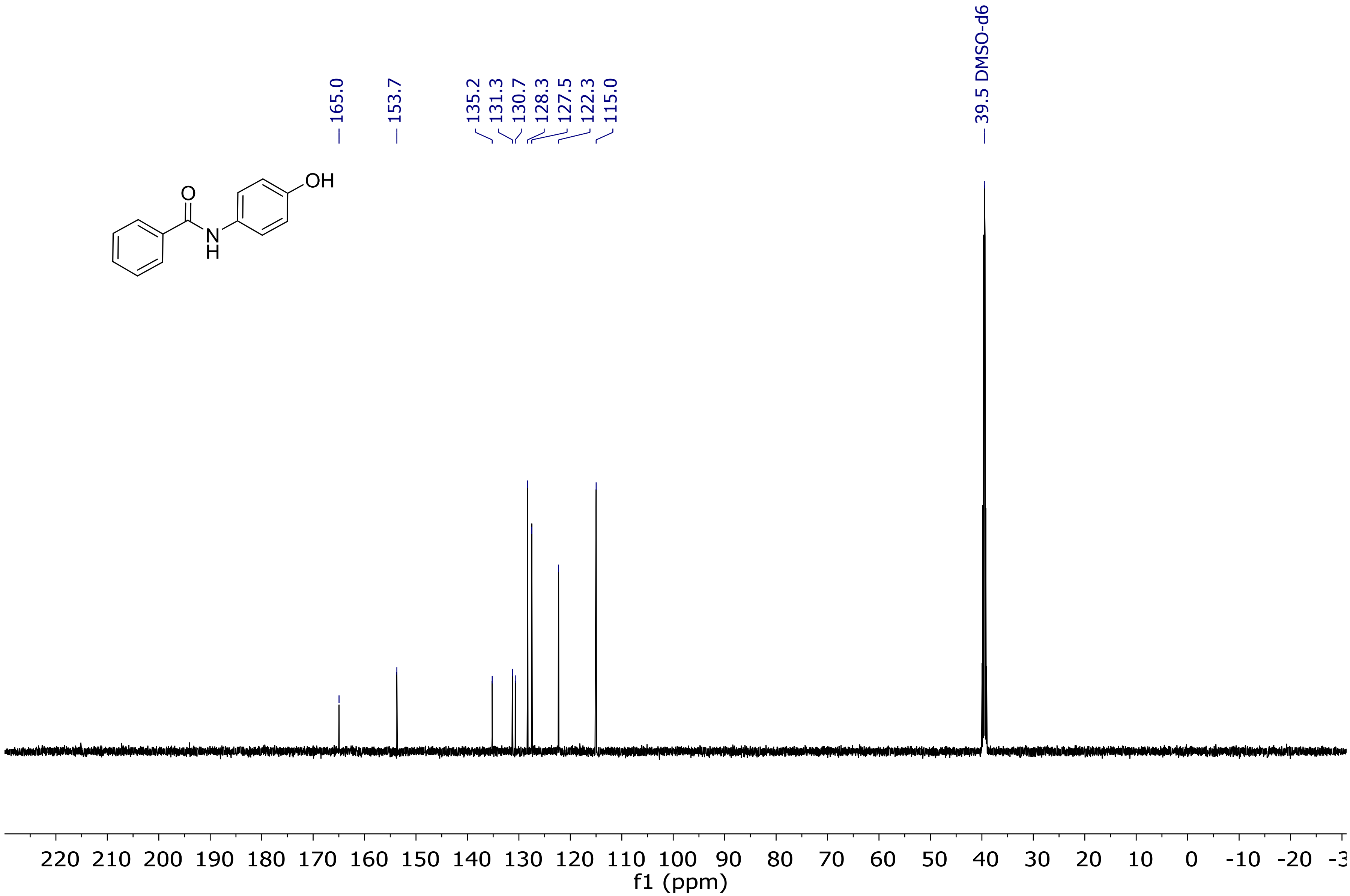

Figure S30. $^{13}C\{^{1}H\}$ NMR (151 MHz, Chloroform-d) of N-(4-hydroxyphenyl)benzamide (3o).

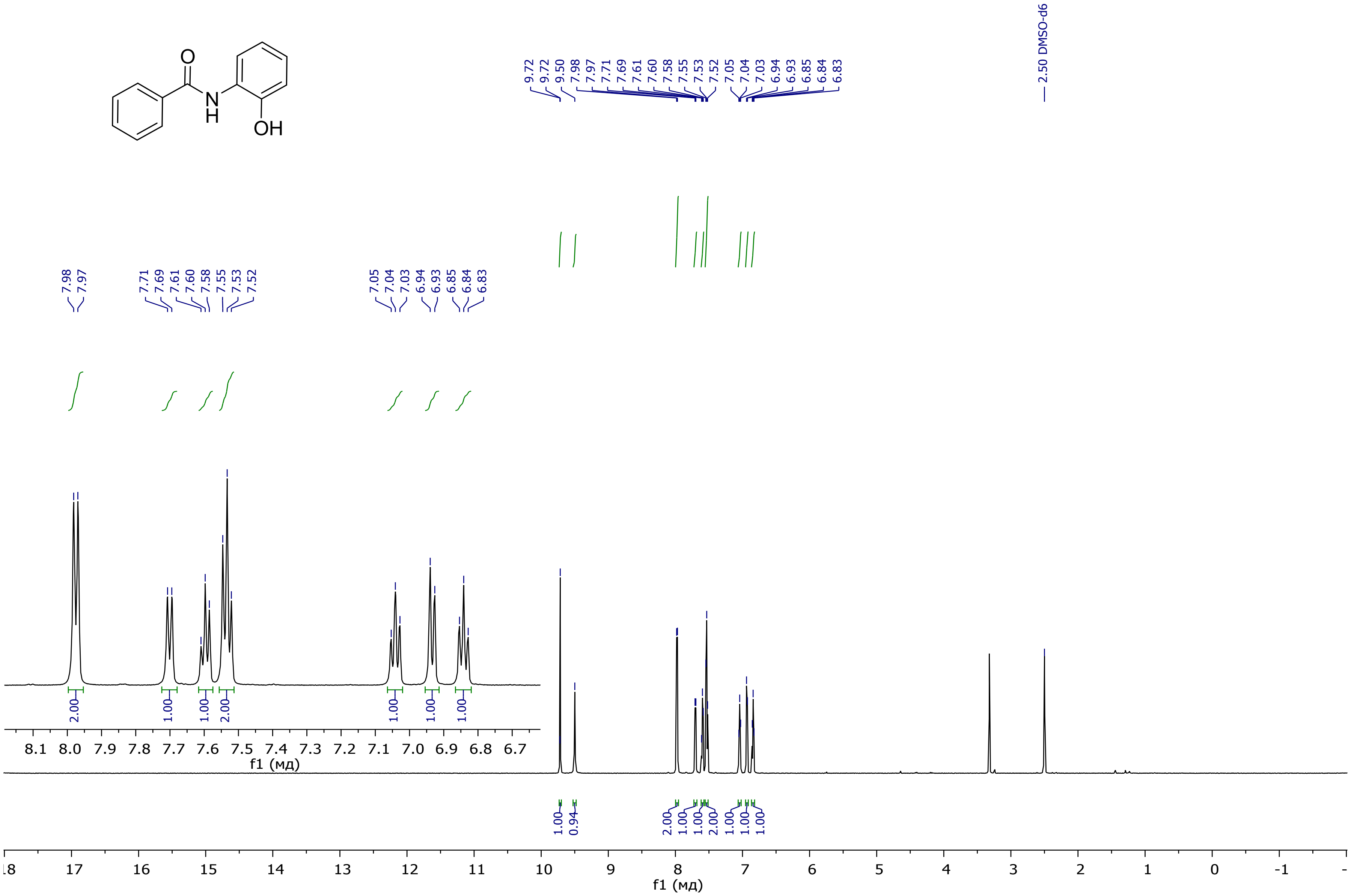

Figure S31. **^{1}H NMR (600 MHz, Chloroform-*d*) of N-(2-hydroxyphenyl)benzamide (3p).**

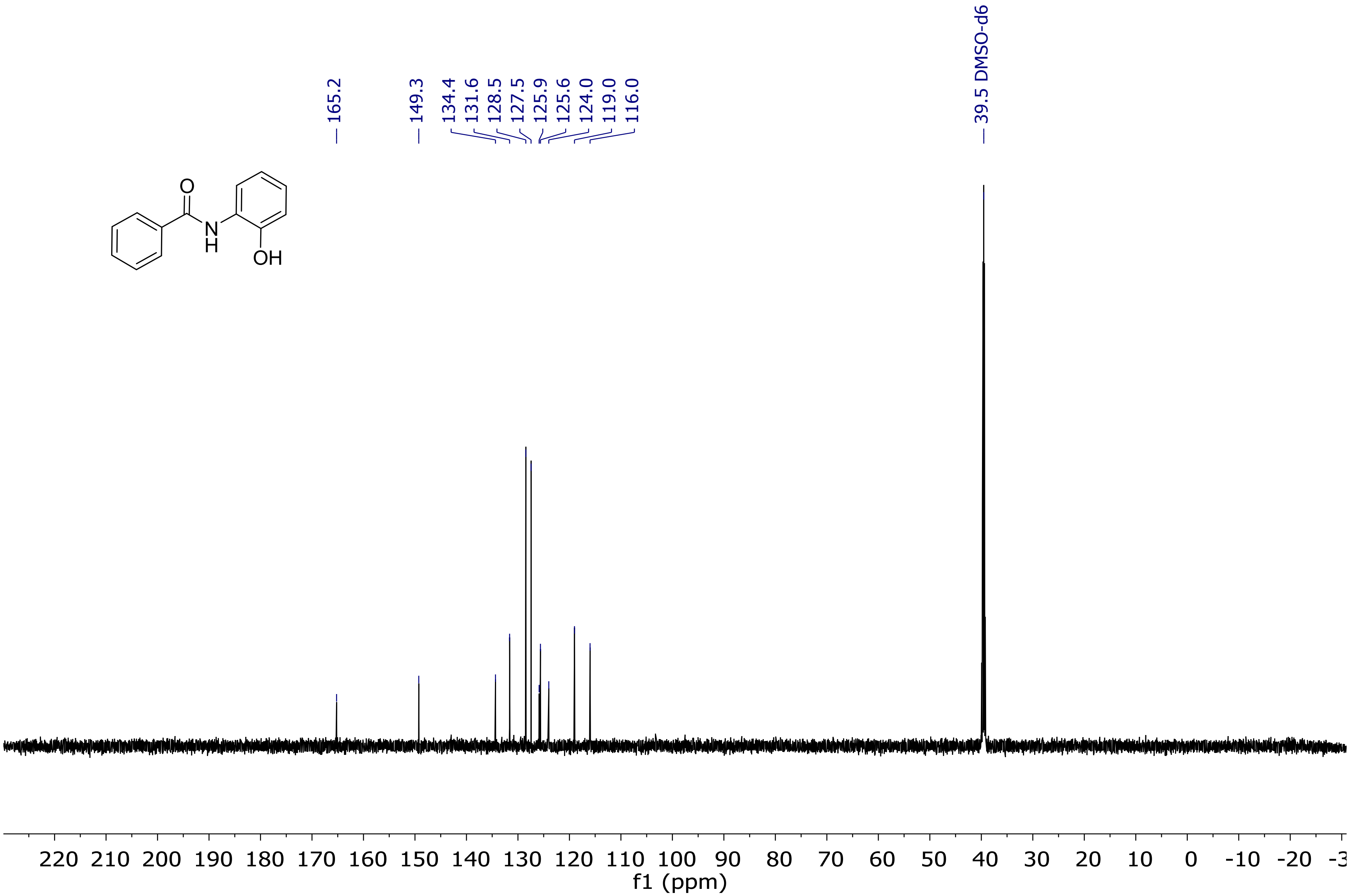

Figure S32. $^{13}C\{^{1}H\}$ NMR (151 MHz, Chloroform-d) of N-(2-hydroxyphenyl)benzamide (3p).

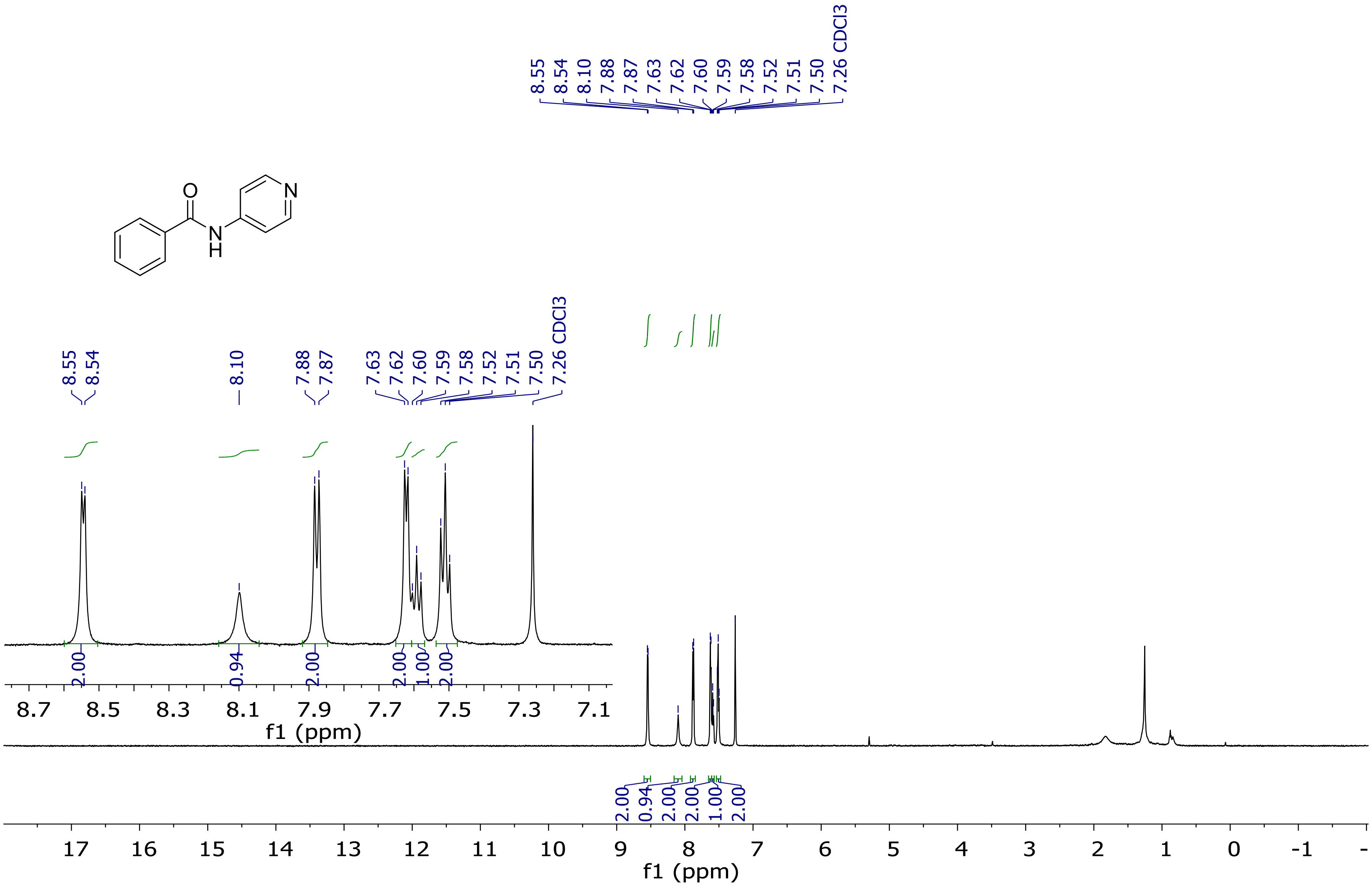

Figure S33. **^{1}H NMR (600 MHz, Chloroform-*d*) of N-(pyridin-4-yl)benzamide (3q).**

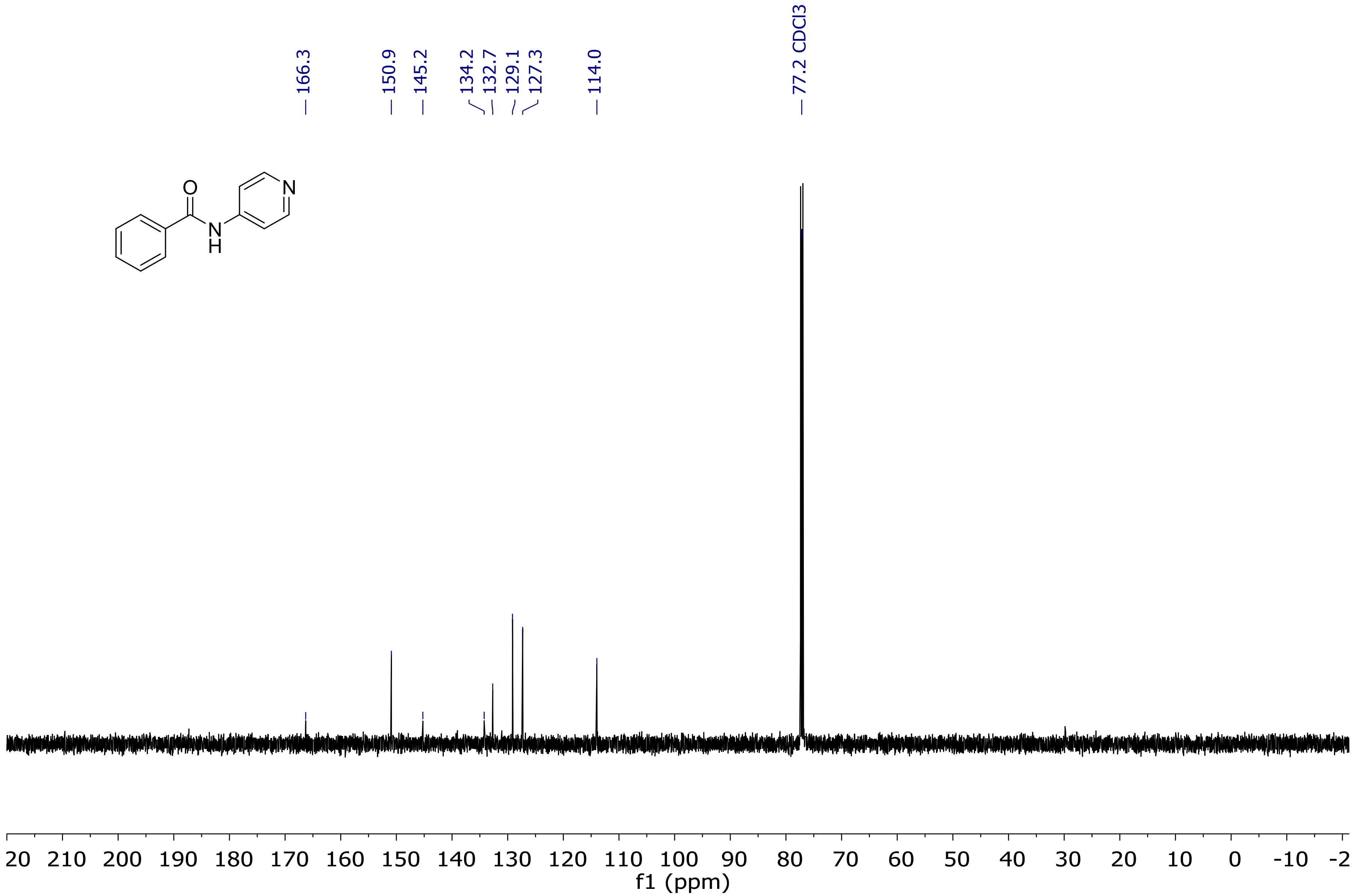

Figure S34. $^{13}C\{^1H\}$ **NMR (151 MHz, Chloroform-d) of N-(pyridin-4-yl)benzamide (3q).**

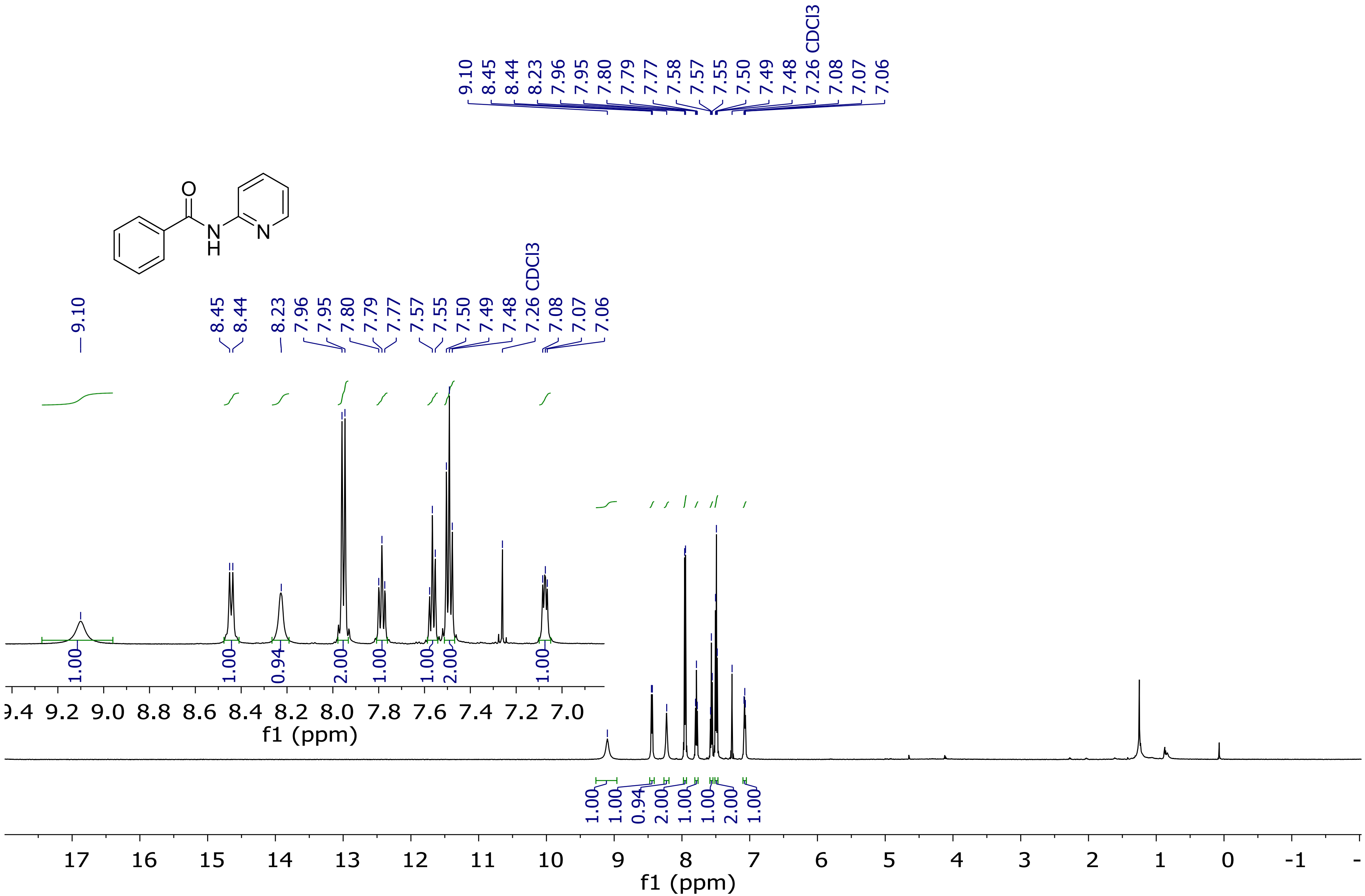

Figure S35. ^{1}H NMR (600 MHz, Chloroform-*d*) of N-(pyridin-2-yl)benzamide (3r).

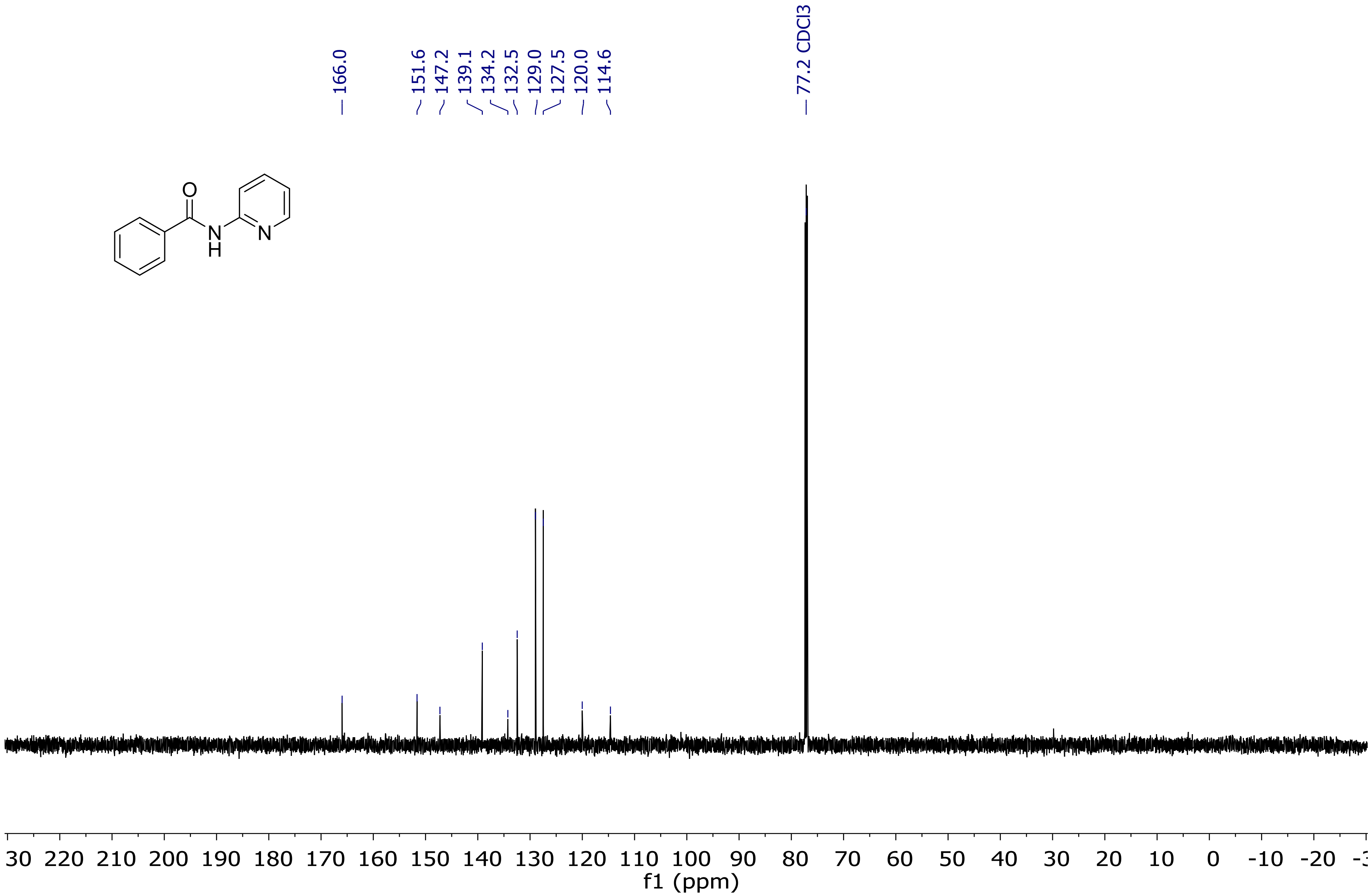

Figure S36. $^{13}C\{^{1}H\}$ **NMR (151 MHz, Chloroform-d) of N-(pyridin-2-yl)benzamide (3r).**

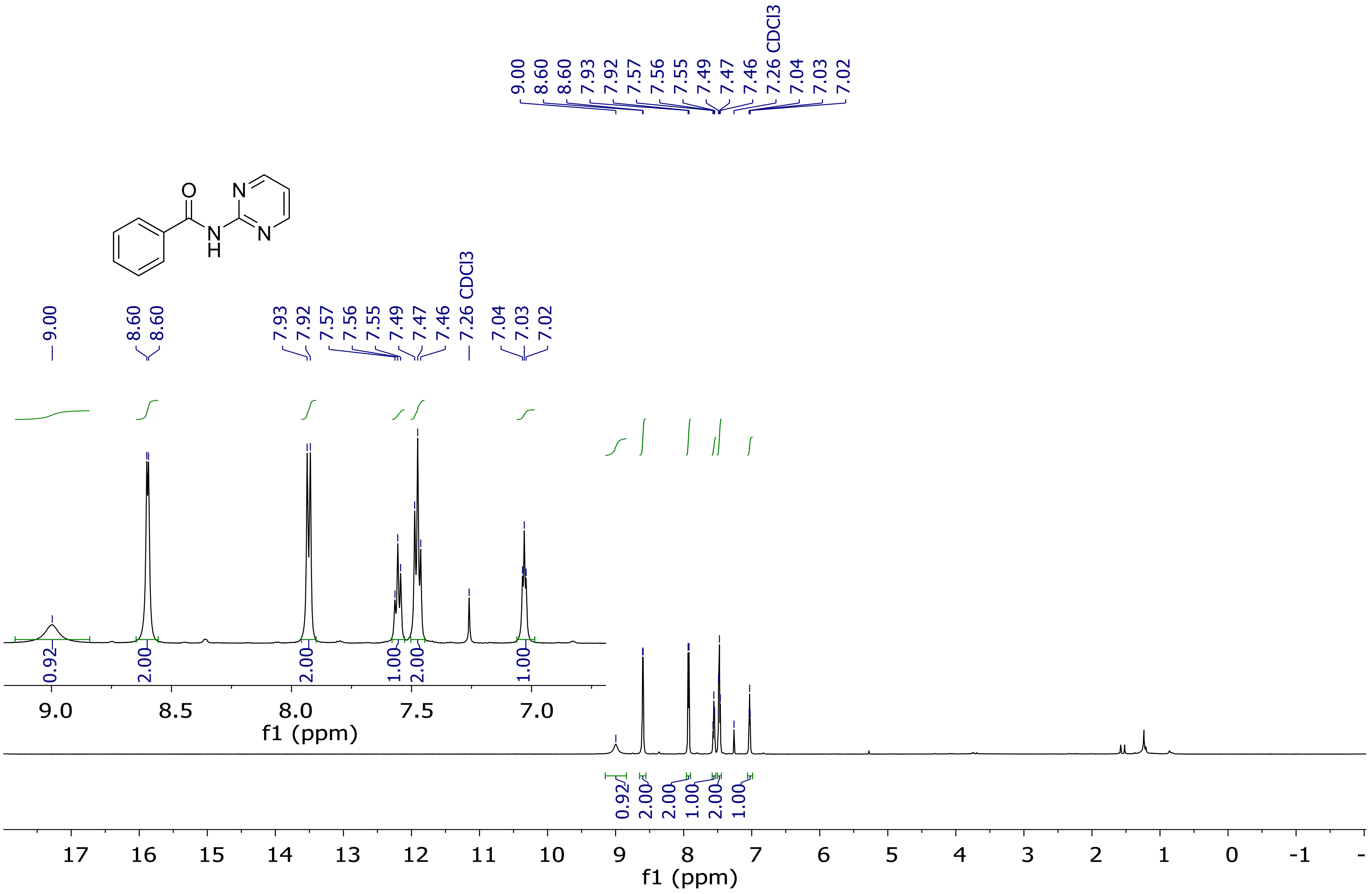

Figure S37. ^{1}H NMR (600 MHz, Chloroform-*d*) of N-(pyrimidin-2-yl)benzamide (3s).

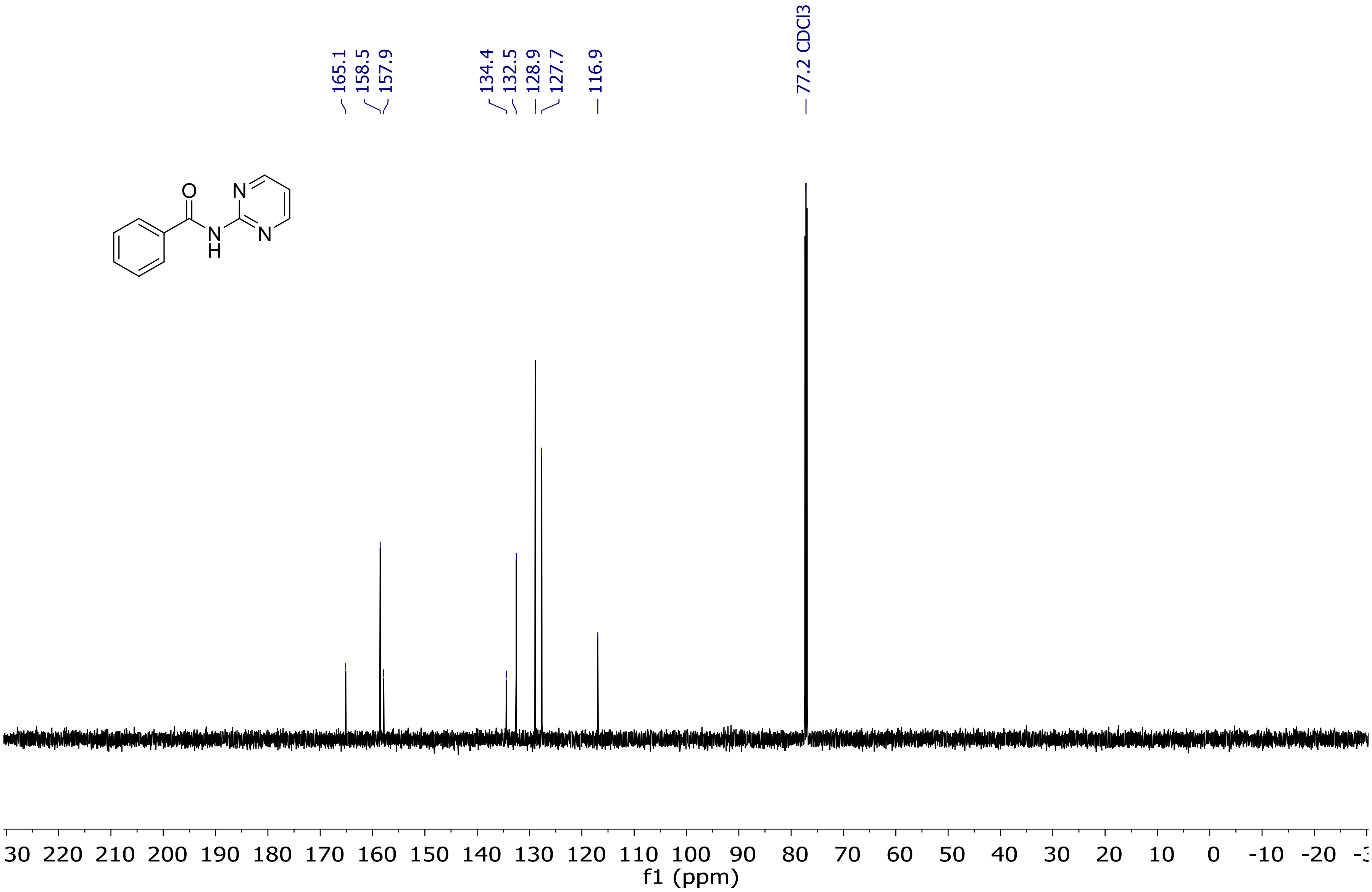

Figure S38. $^{13}C\{^{1}H\}$ **NMR (151 MHz, Chloroform-d) of N-(pyrimidin-2-yl)benzamide (3s).**

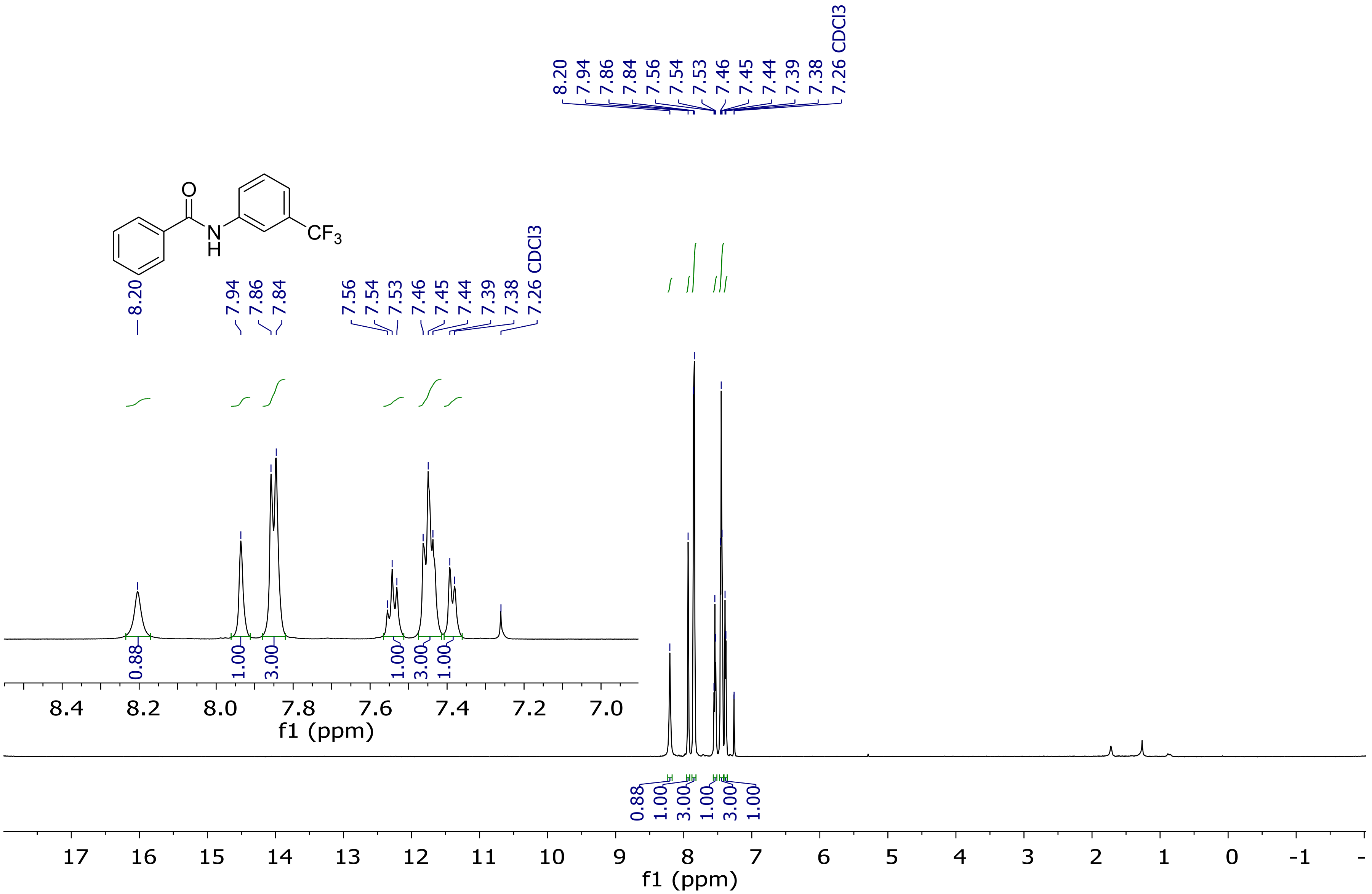

Figure S39. ¹H NMR (600 MHz, Chloroform-*d*) of N-(3-(trifluoromethyl)phenyl)benzamide (3t).

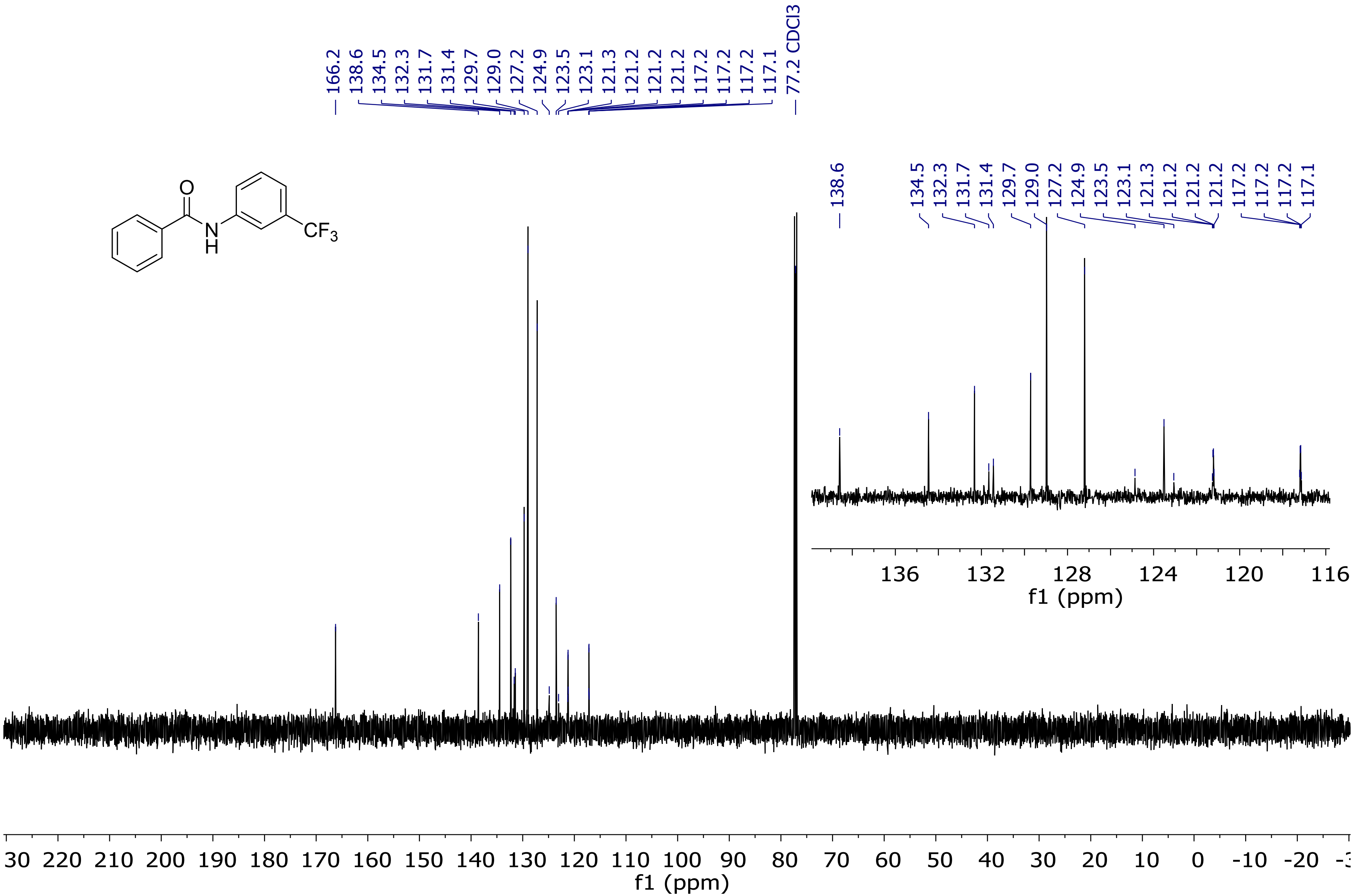

Figure S40. $^{13}C\{^{1}H\}$ **NMR (151 MHz, Chloroform-d) of N-(3-(trifluoromethyl)phenyl)benzamide (3t).**

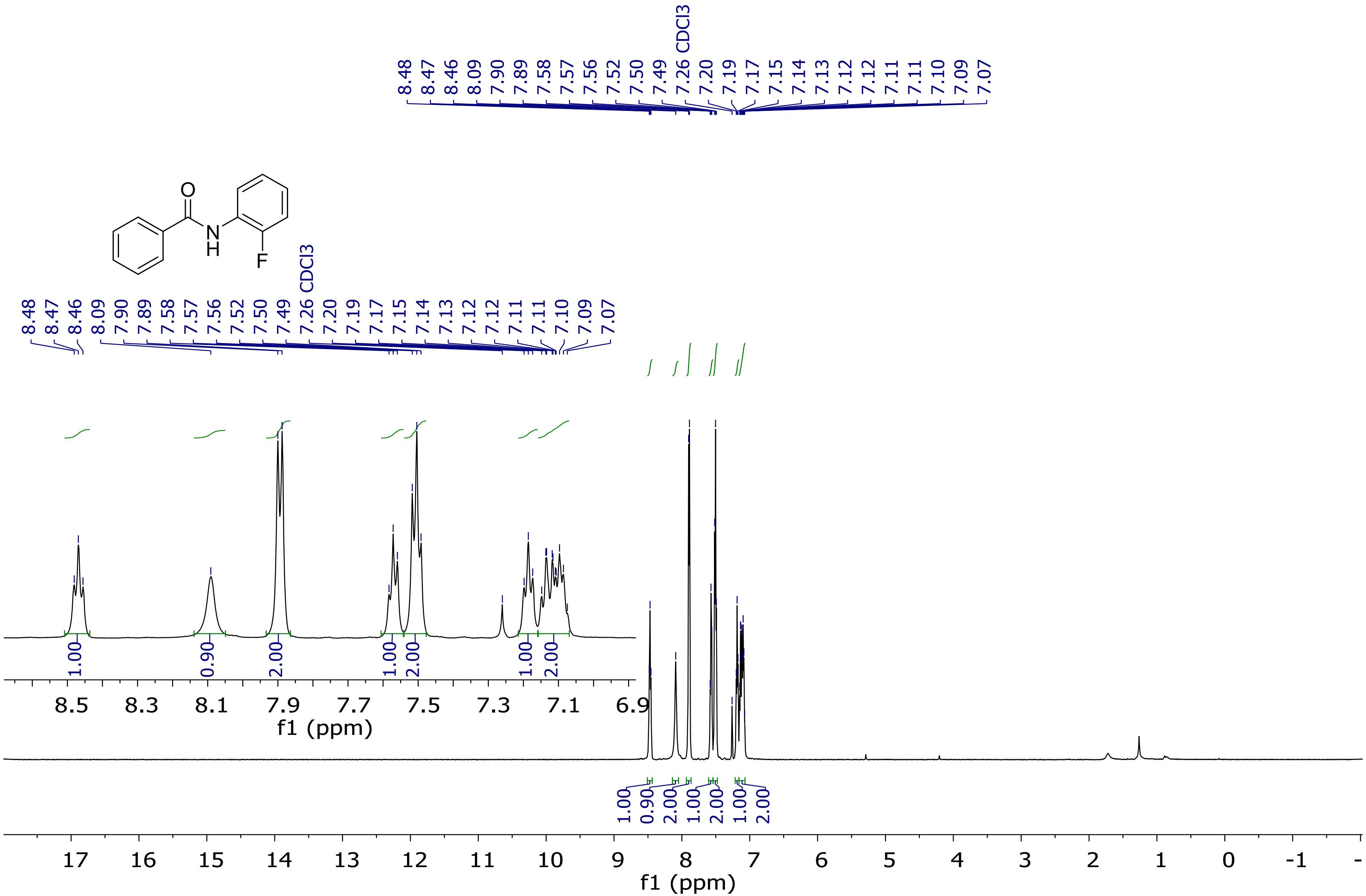

Figure S41. ¹H NMR (600 MHz, Chloroform-*d*) of N-(2-fluorophenyl)benzamide (3u).

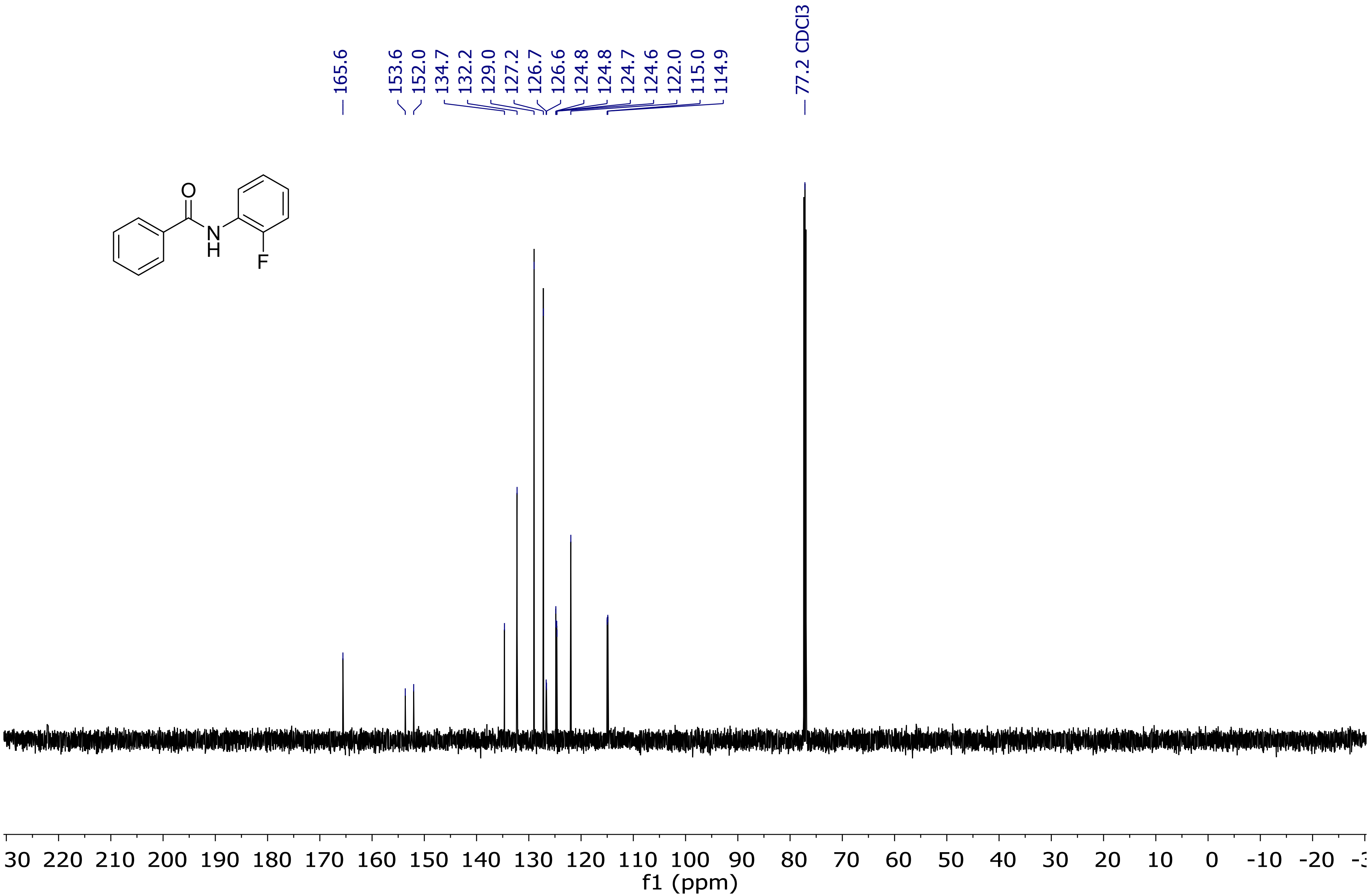

Figure S42. $^{13}C\{^{1}H\}$ **NMR (151 MHz, Chloroform-d) of N-(2-fluorophenyl)benzamide (3u).**

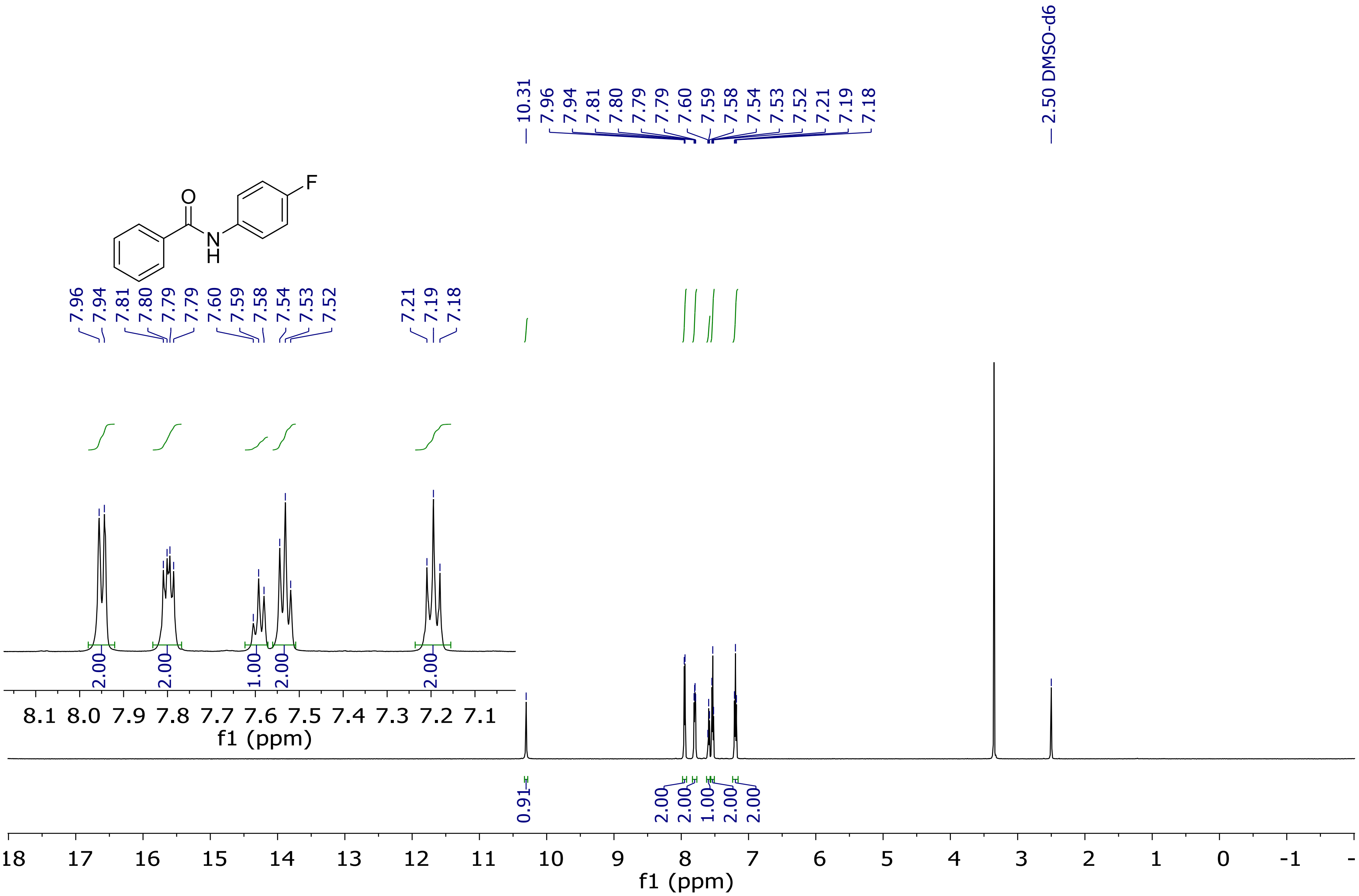

Figure S43. **^{1}H NMR (600 MHz, Chloroform-*d*) of N-(4-fluorophenyl)benzamide (3v).**

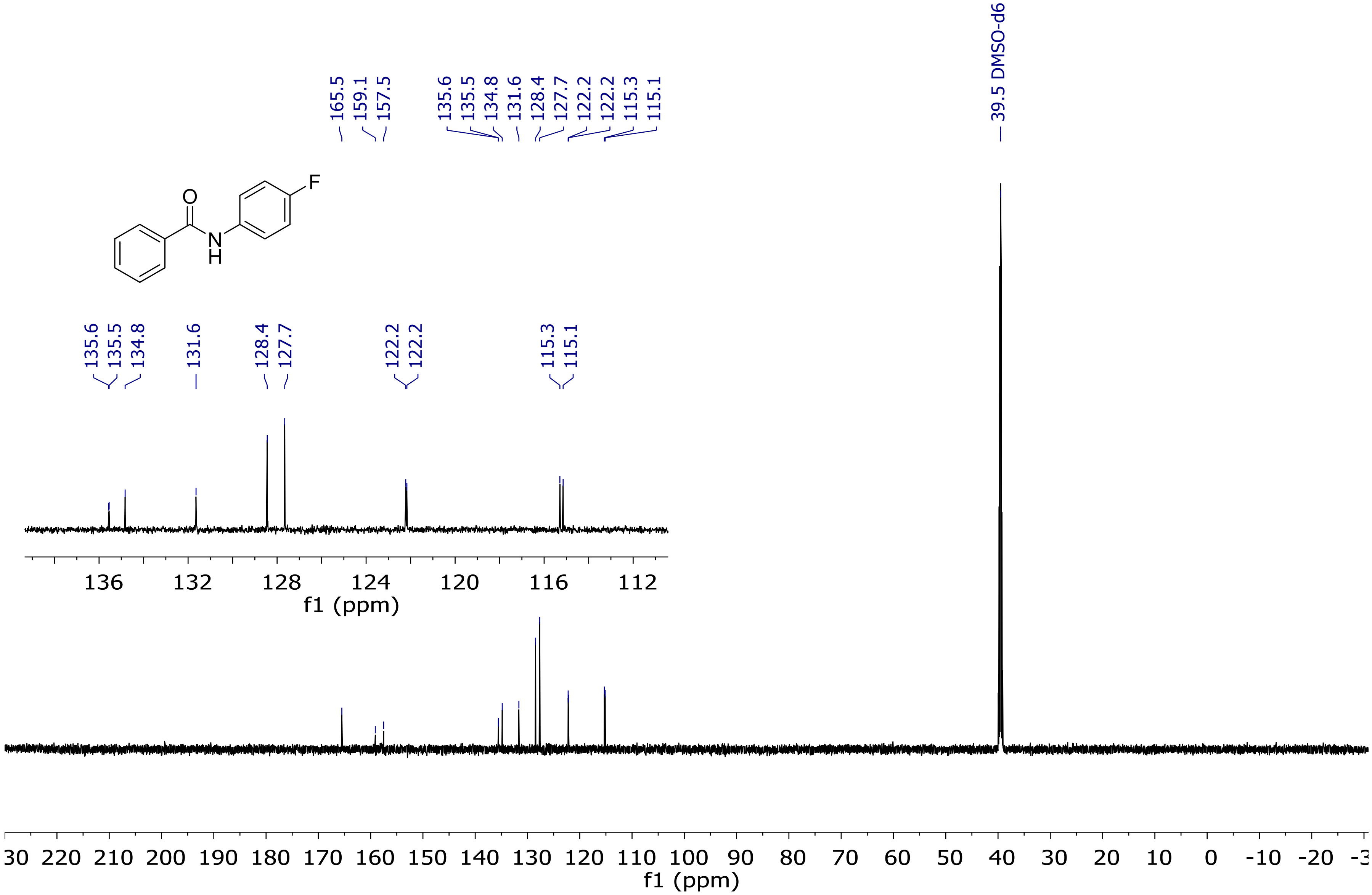

Figure S44. $^{13}C\{^{1}H\}$ NMR (151 MHz, Chloroform-d) of N-(4-fluorophenyl)benzamide (3v).

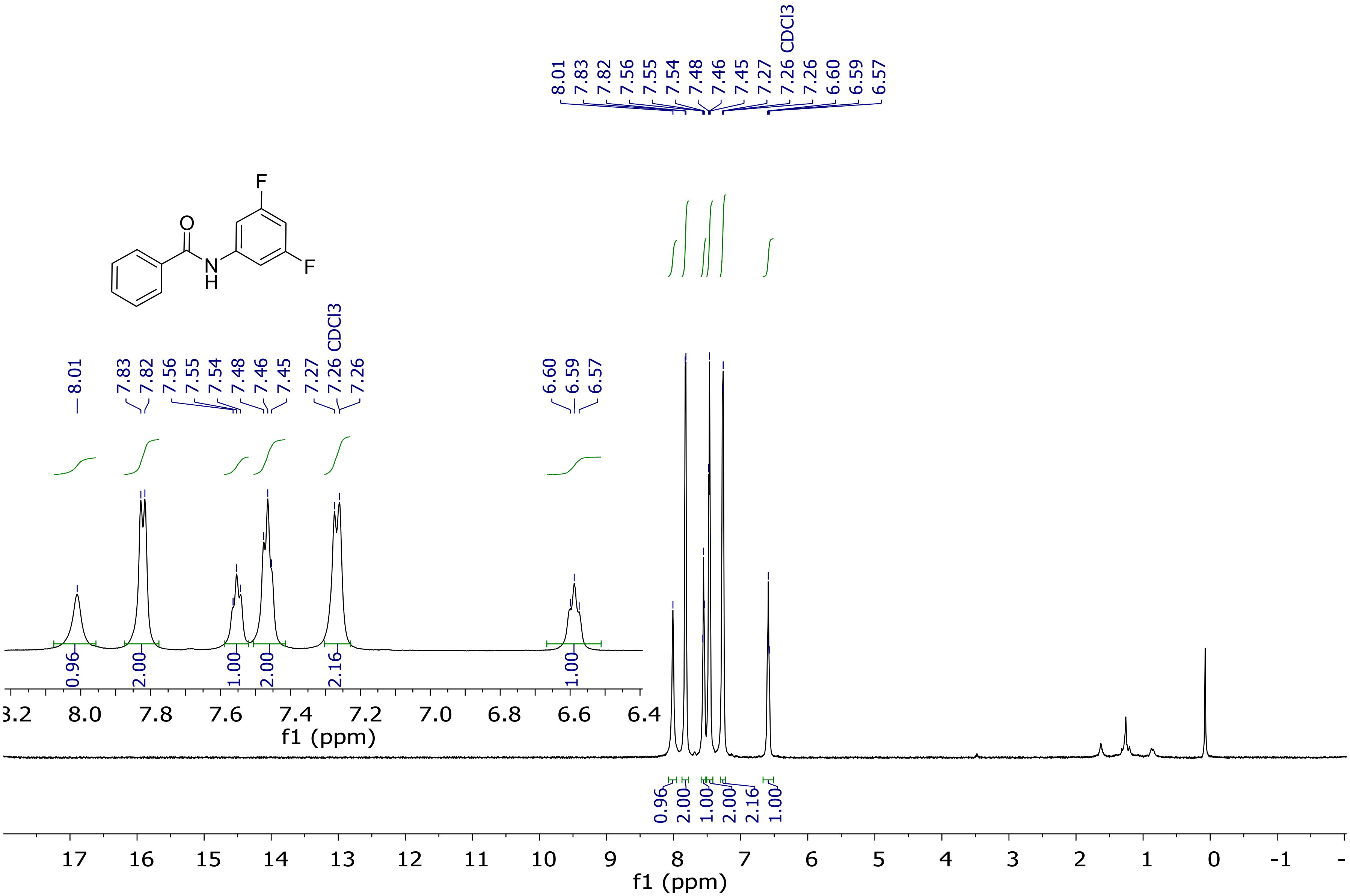

Figure S45. ^{1}H NMR (600 MHz, Chloroform-*d*) of N-(3,5-difluorophenyl)benzamide (3w).

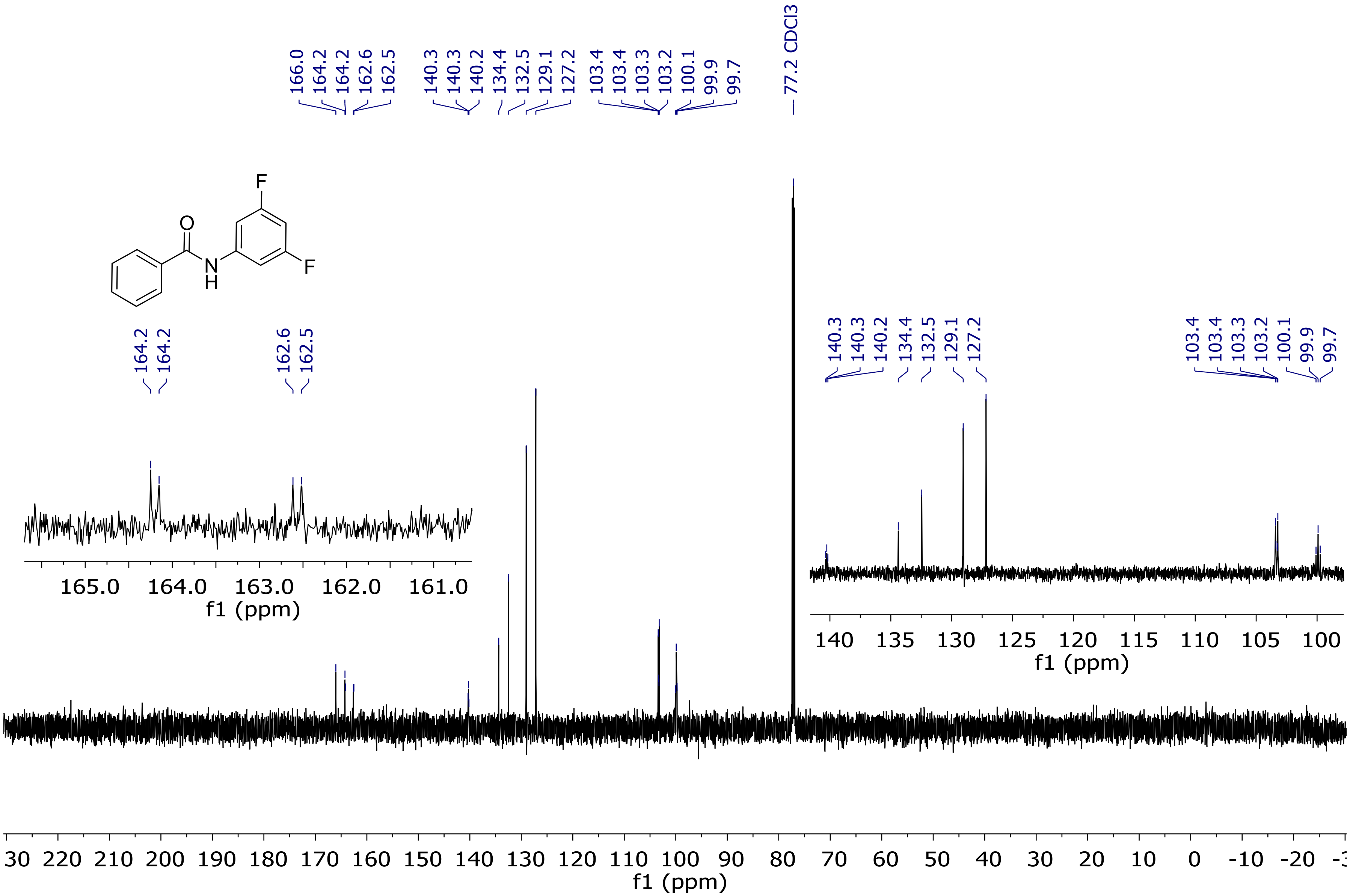

Figure S46. $^{13}C\{^{1}H\}$ **NMR (151 MHz, Chloroform-d) of N-(3,5-difluorophenyl)benzamide (3w).**

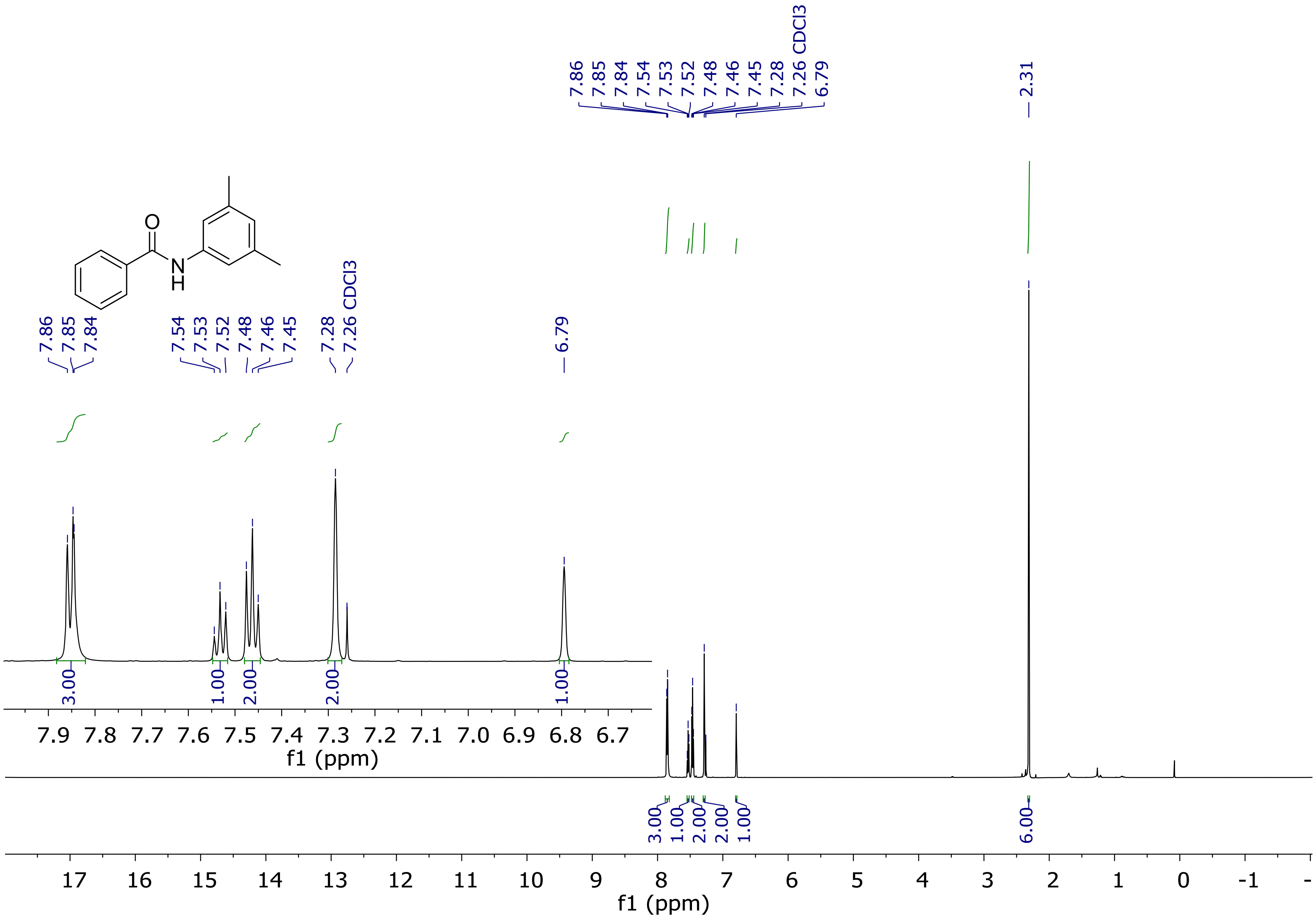

Figure S47. ¹H NMR (600 MHz, Chloroform-*d*) N-(3,5-dimethylphenyl)benzamide (3x)

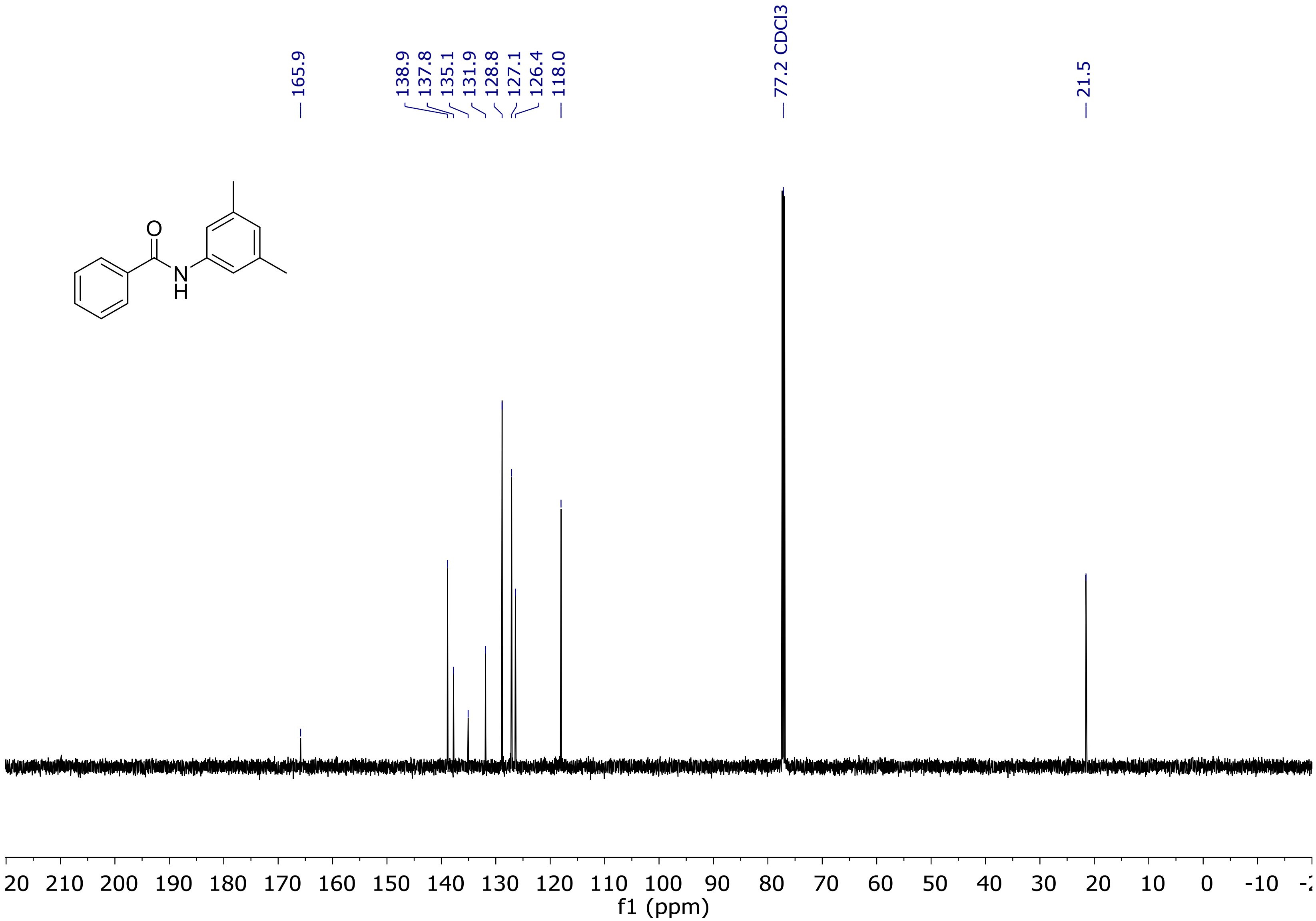

Figure S48. $^{13}C\{^{1}H\}$ **NMR (151 MHz, Chloroform-d) of N-(3,5-dimethylphenyl)benzamide (3x)**

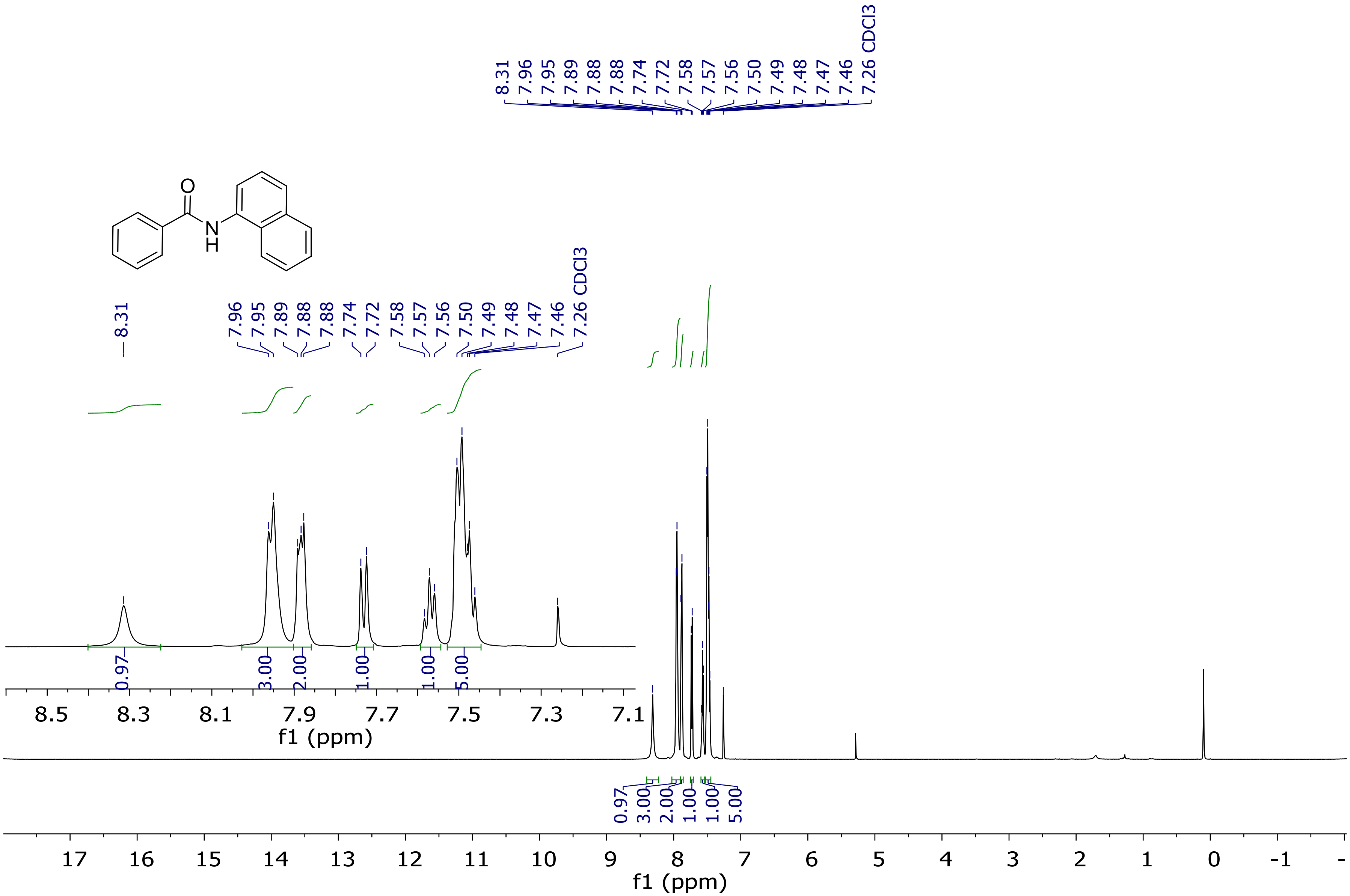

Figure S49. ^{1}**H NMR (600 MHz, Chloroform-*d*) of N-(naphthalen-1-yl)benzamide (3y).**

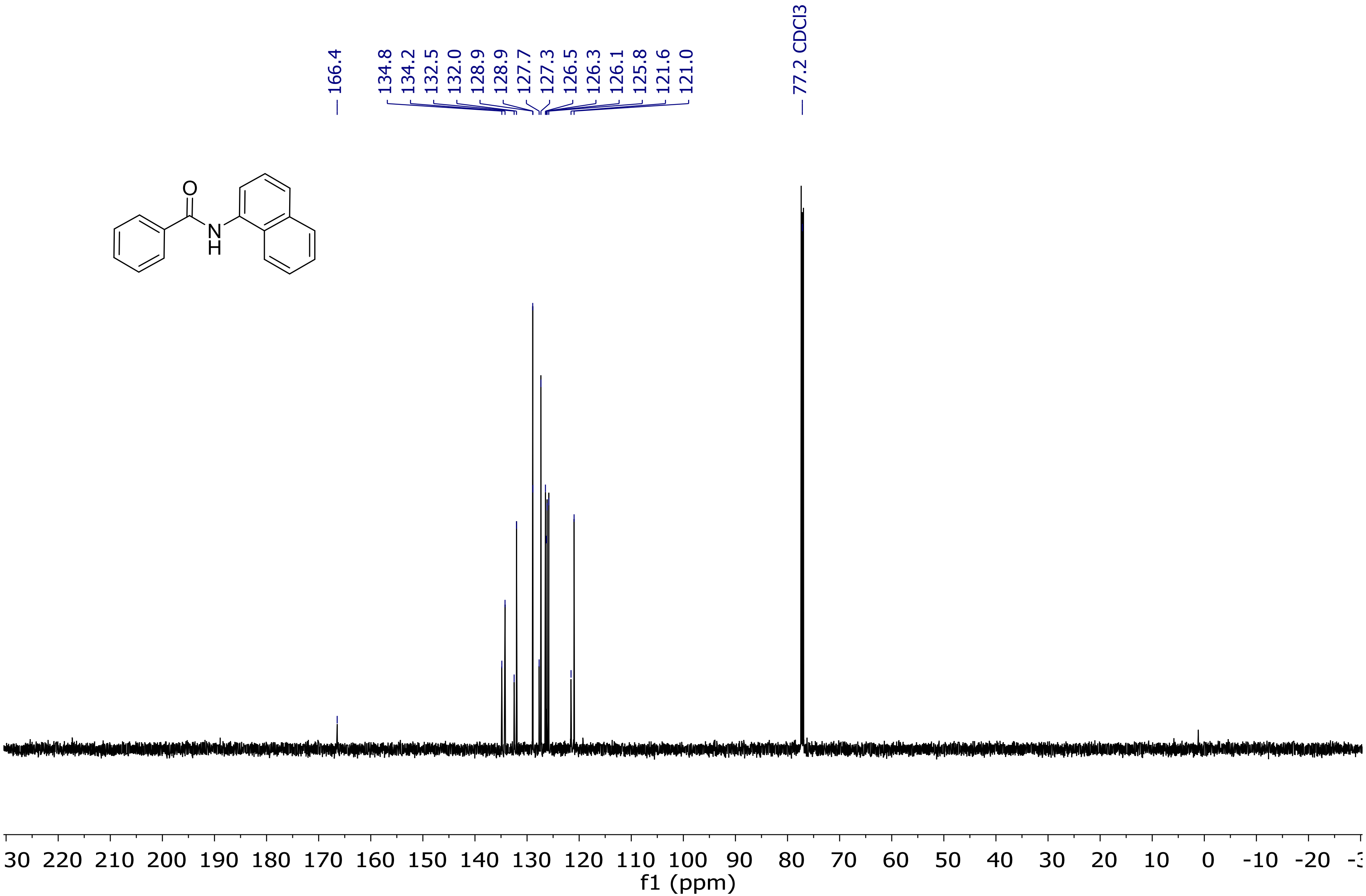

Figure S50. $^{13}C\{^{1}H\}$ **NMR (151 MHz, Chloroform-d) of N-(naphthalen-1-yl)benzamide (3y).**

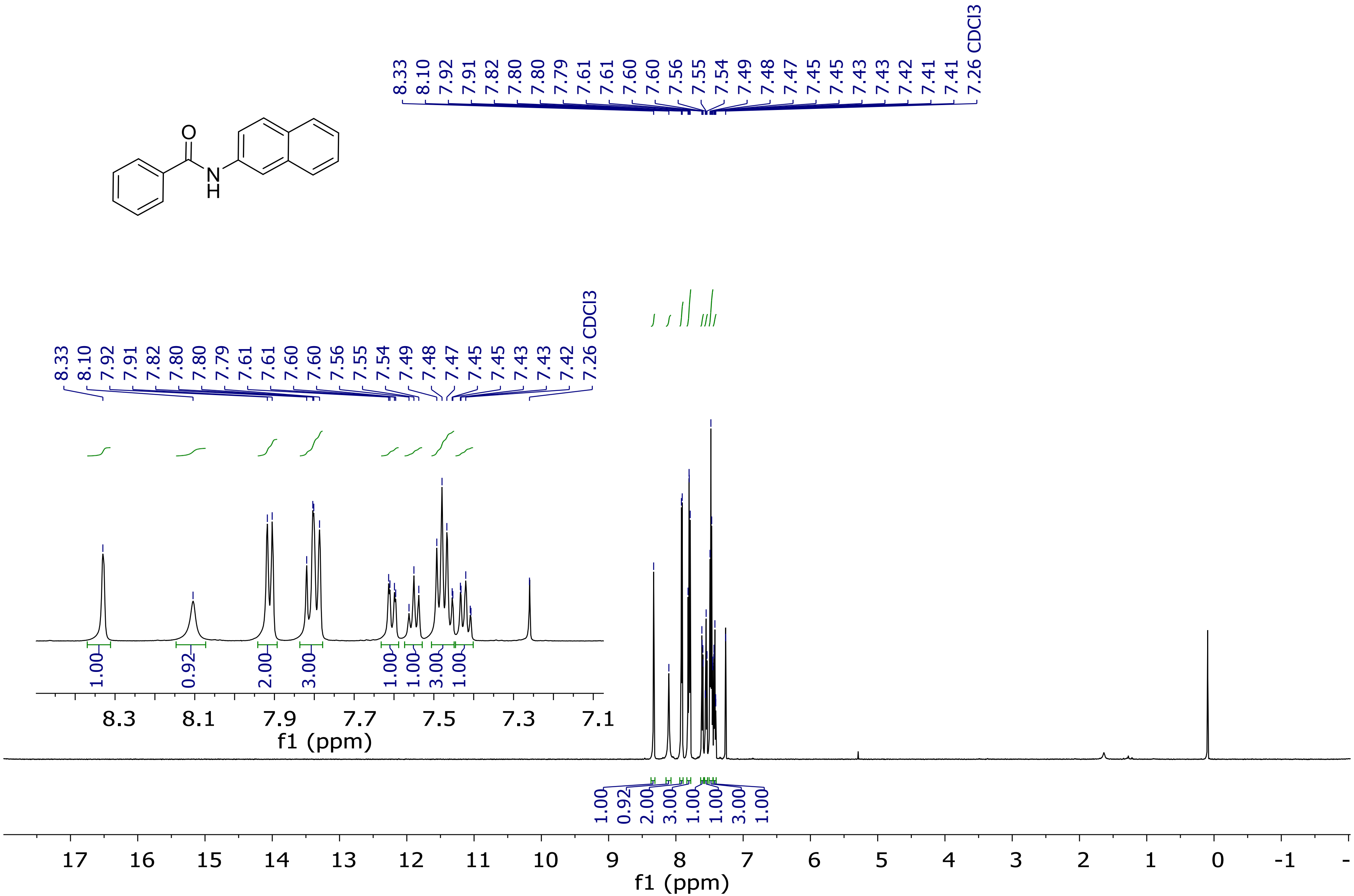

Figure S51. ^{1}**H NMR (600 MHz, Chloroform-*d*) of N-(naphthalen-2-yl)benzamide (3z).**

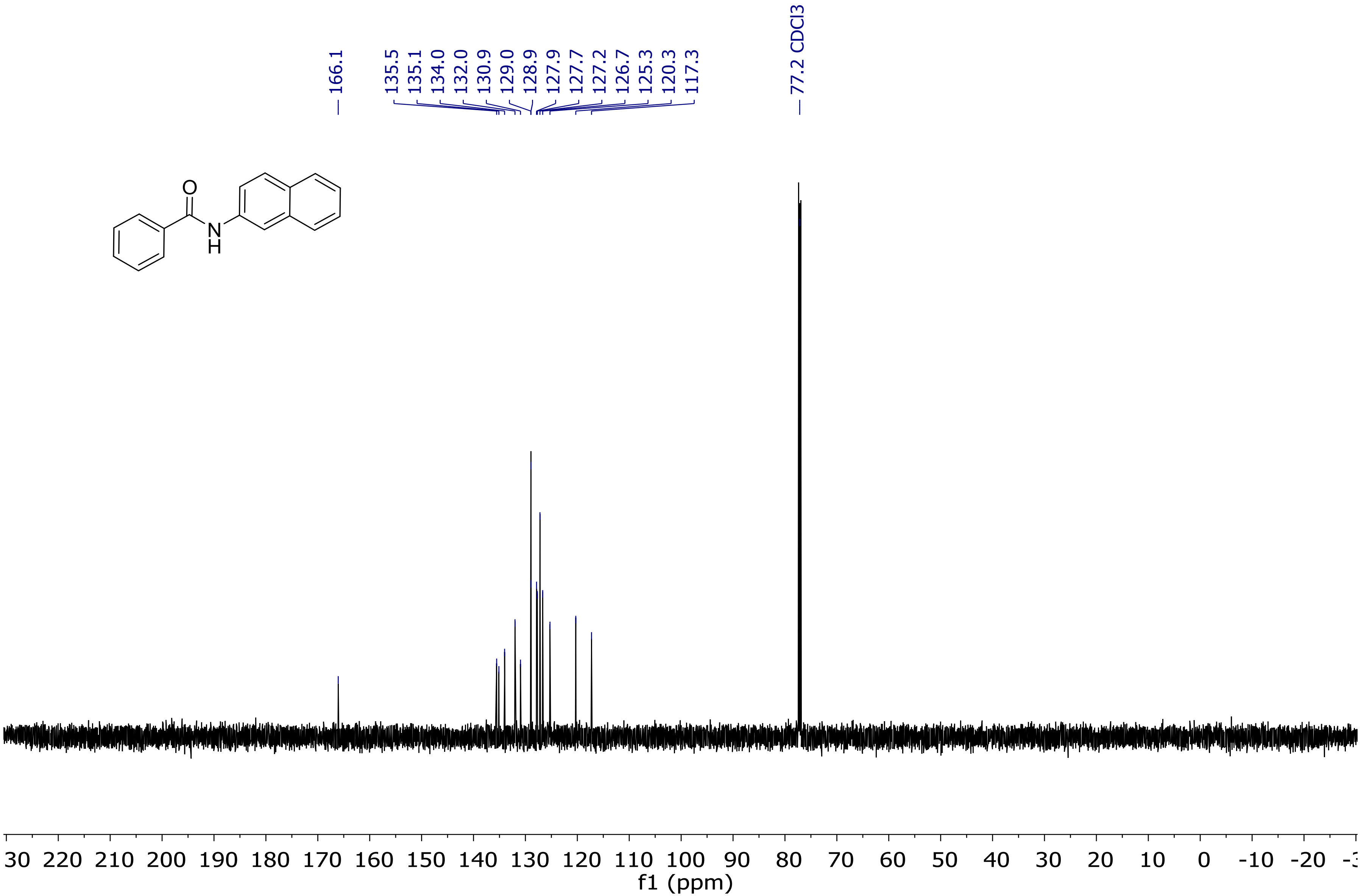

Figure S52. $^{13}C\{^1H\}$ **NMR (151 MHz, Chloroform-d) of N-(naphthalen-2-yl)benzamide (3z).**

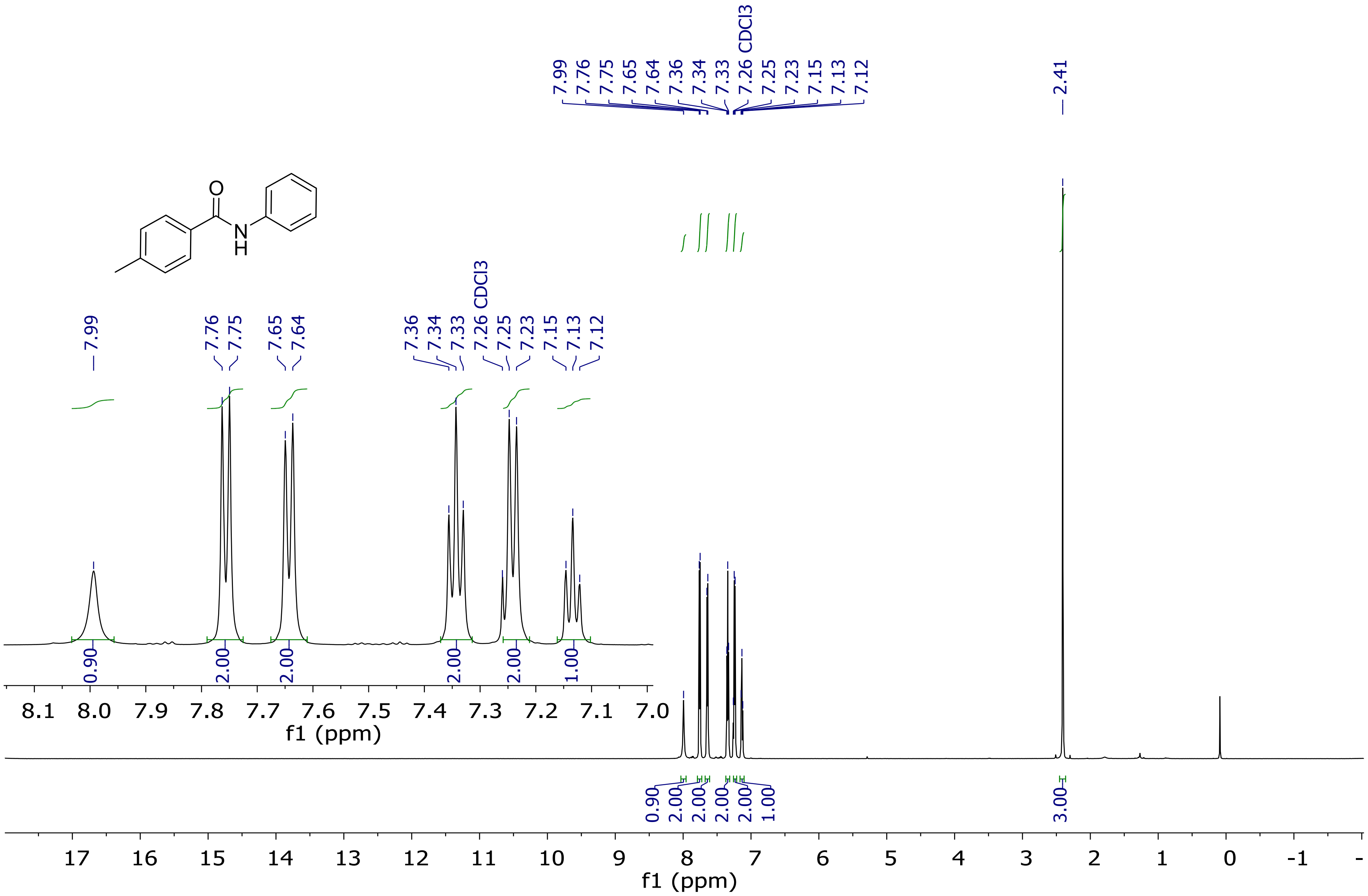

Figure S53. **^{1}H NMR (600 MHz, Chloroform-*d*) of 4-methyl-N-phenylbenzamide (3db).**

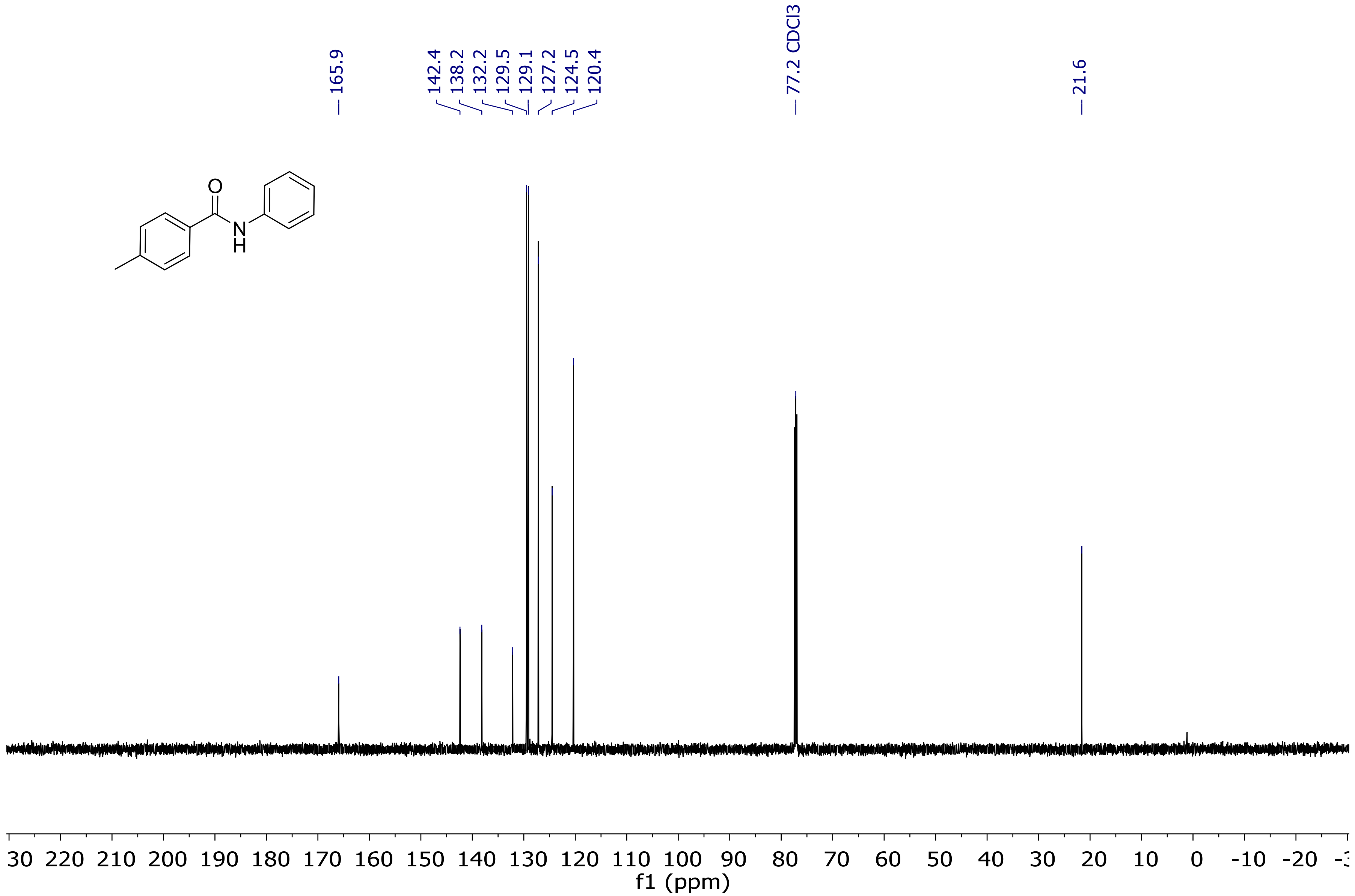

Figure S54. $^{13}C\{^1H\}$ **NMR (151 MHz, Chloroform-d) of 4-methyl-N-phenylbenzamide (3db).**

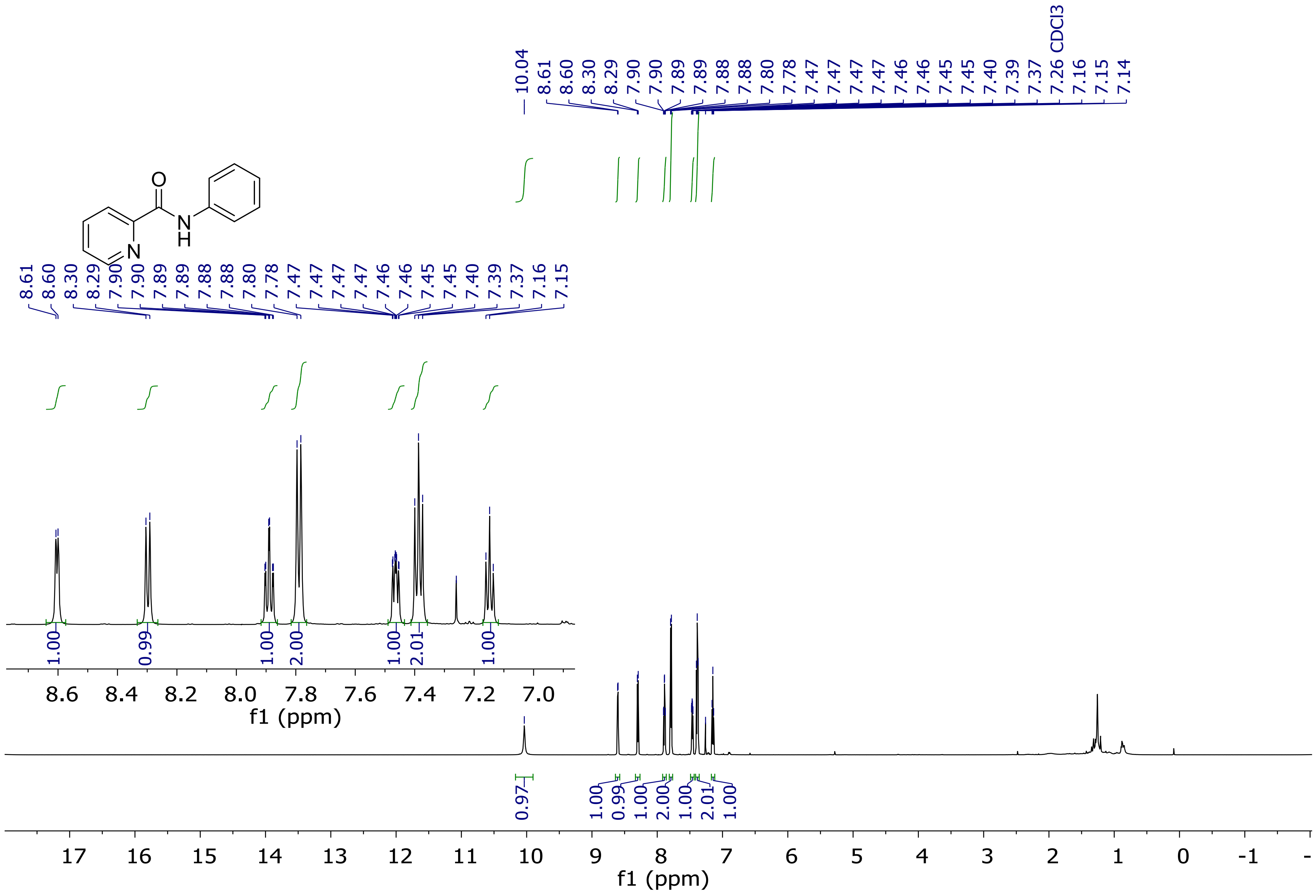

Figure S55. ^{1}H NMR (600 MHz, Chloroform-*d*) of N-phenylpicolinamide (3dc).

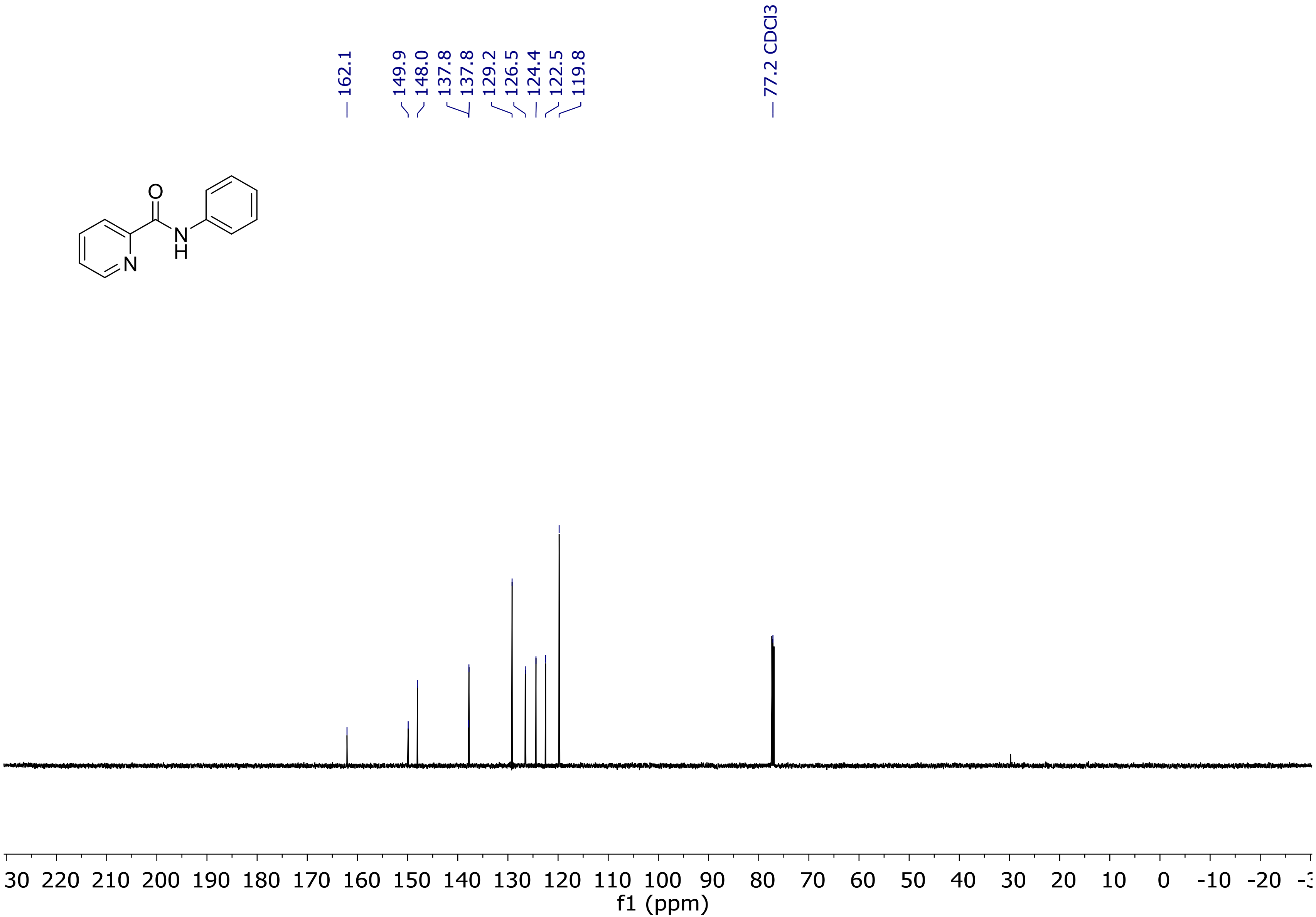

Figure S56. $^{13}C\{^{1}H\}$ **NMR (151 MHz, Chloroform-d) of N-phenylpicolinamide (3dc).**

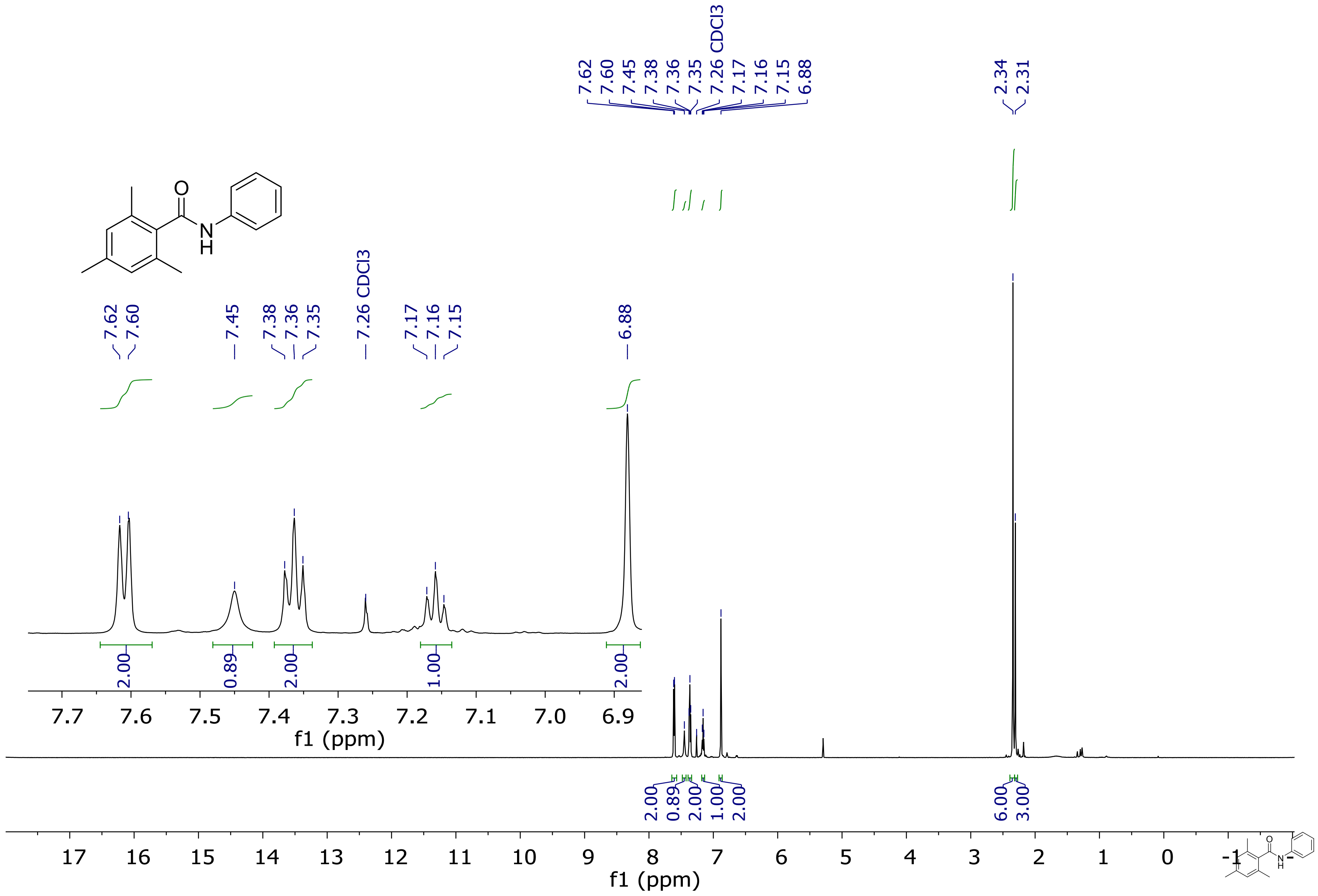

Figure S57. [1]**H NMR (600 MHz, Chloroform-*d*) of 2,4,6-trimethyl-N-phenylbenzamide (3dd).**

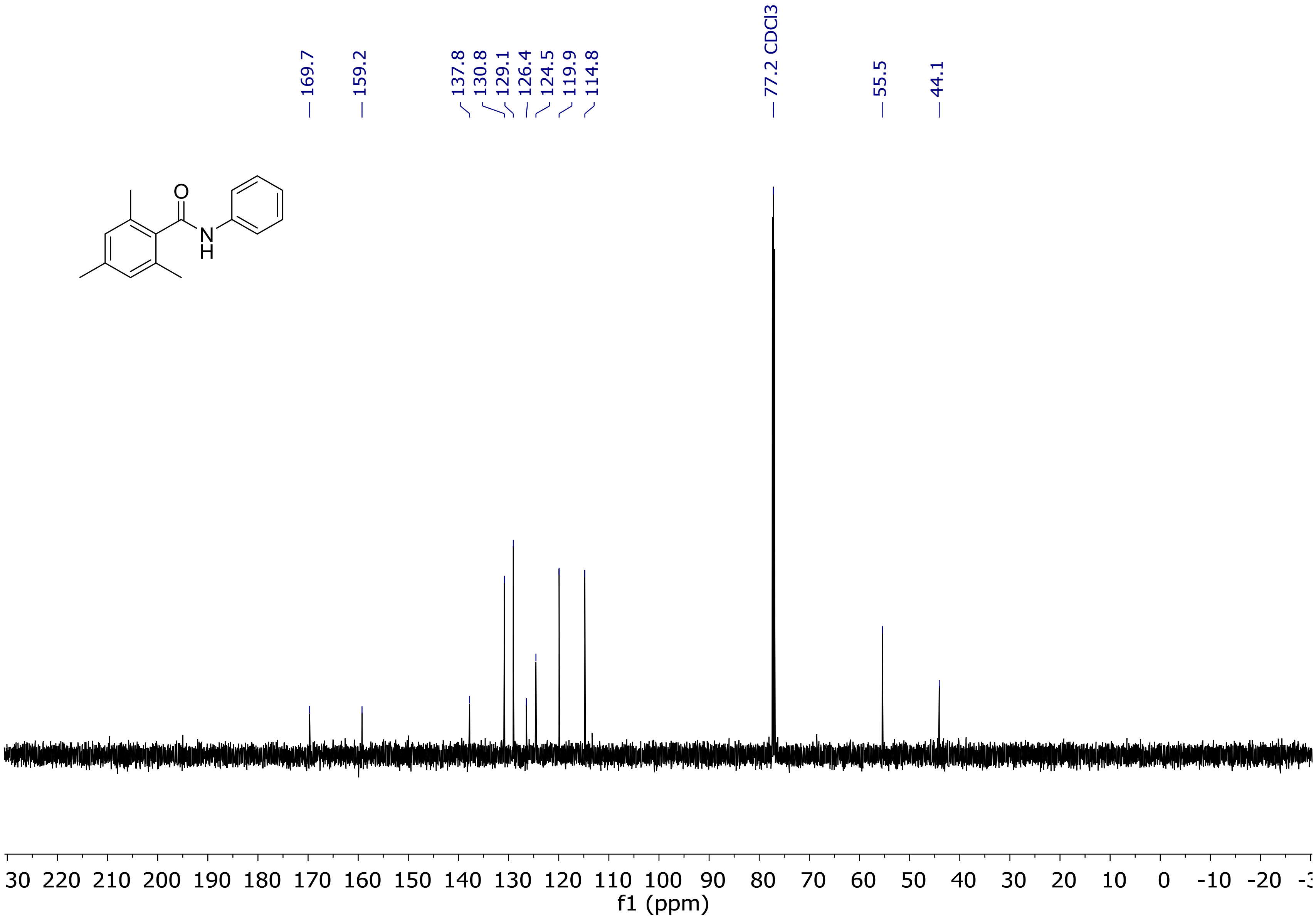

Figure S58. $^{13}C\{^1H\}$ **NMR (151 MHz, Chloroform-d) of 2,4,6-trimethyl-N-phenylbenzamide (3dd).**

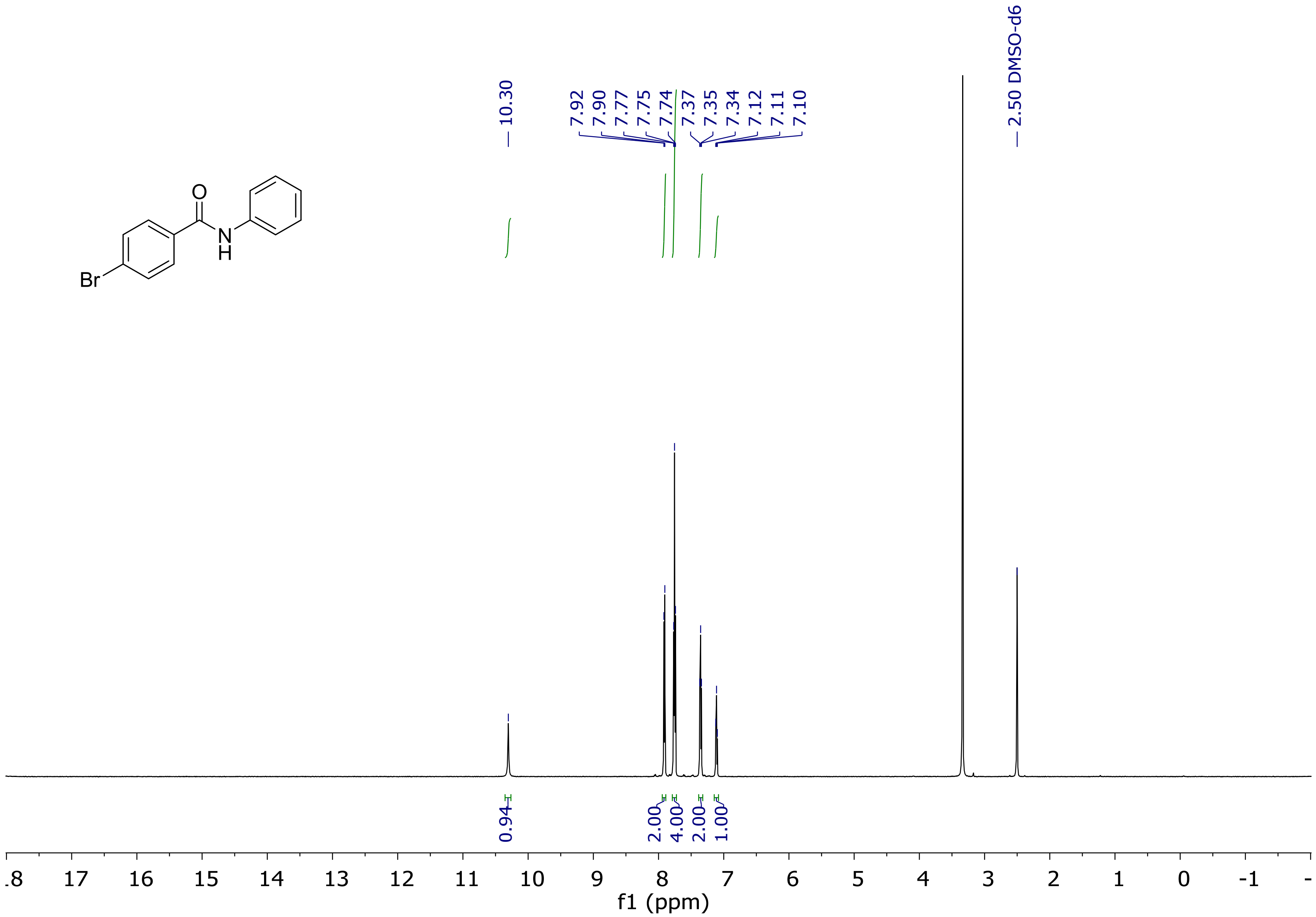

Figure S59. ^{1}H NMR (600 MHz, Chloroform-*d*) of 4-bromo-N-phenylbenzamide (3de).

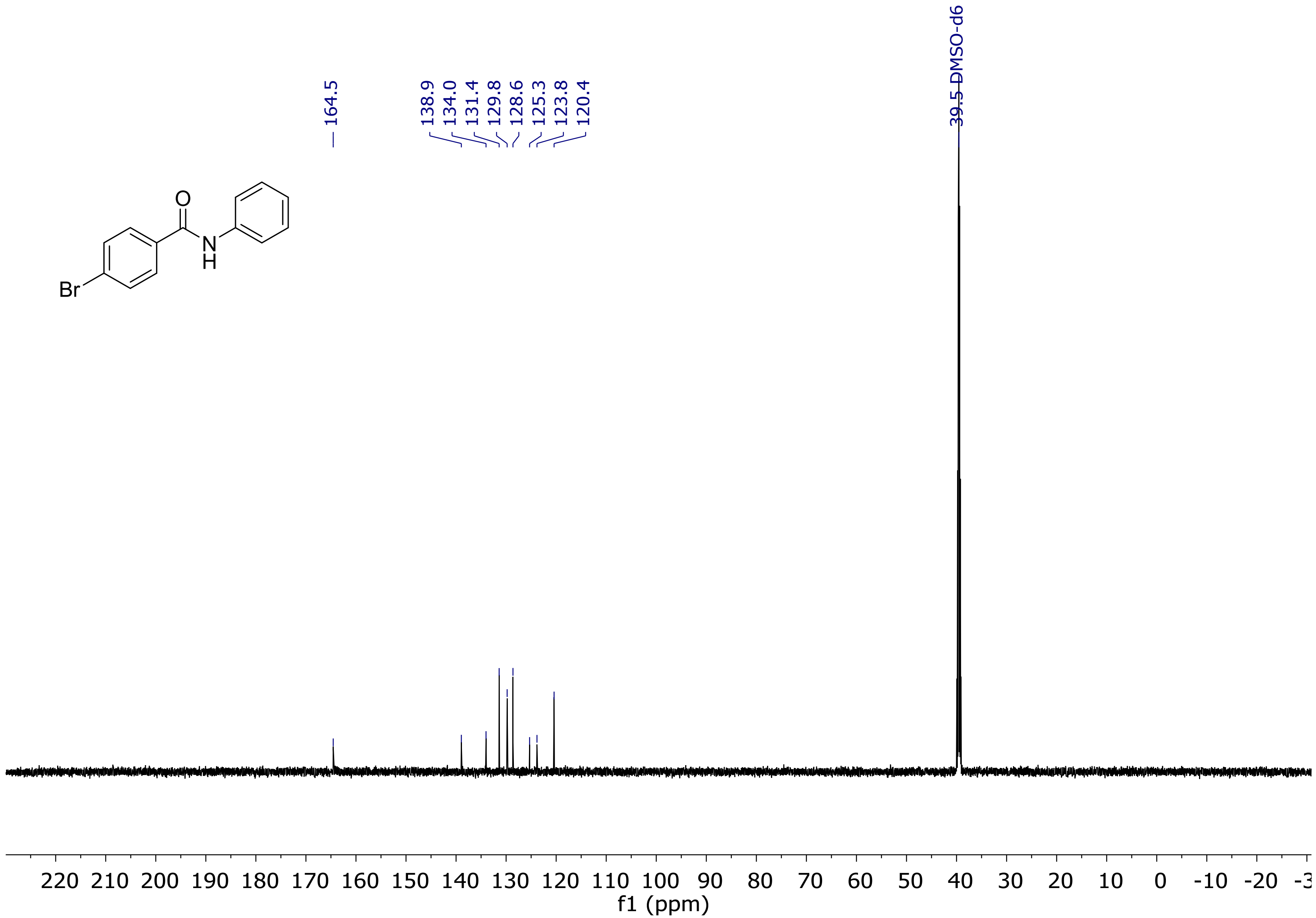

Figure S60. $^{13}C\{^{1}H\}$ NMR (151 MHz, Chloroform-d) of 4-bromo-N-phenylbenzamide (3de).

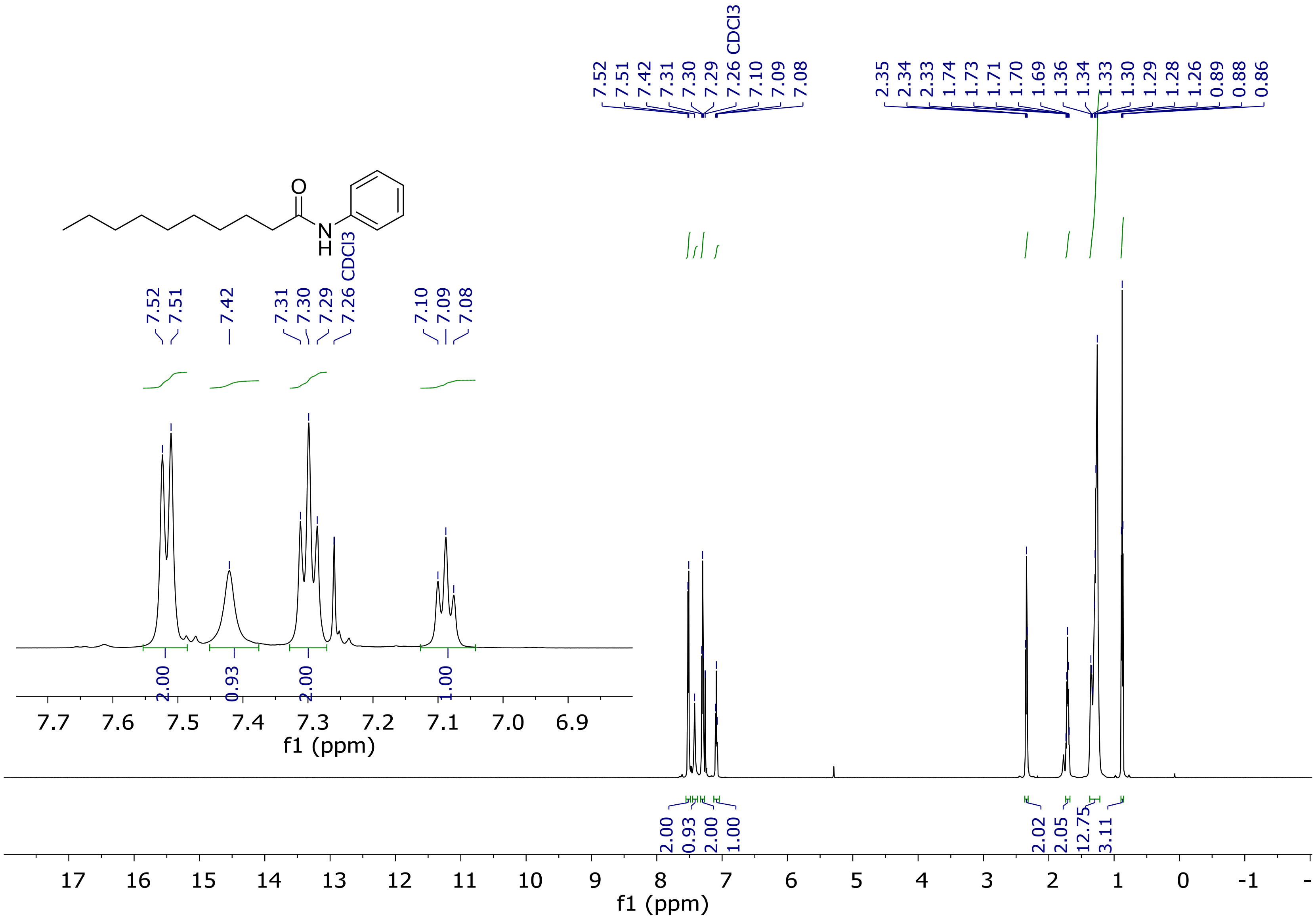

Figure S61. **^{1}H NMR (600 MHz, Chloroform-*d*) of N-phenyldecanamide (3df).**

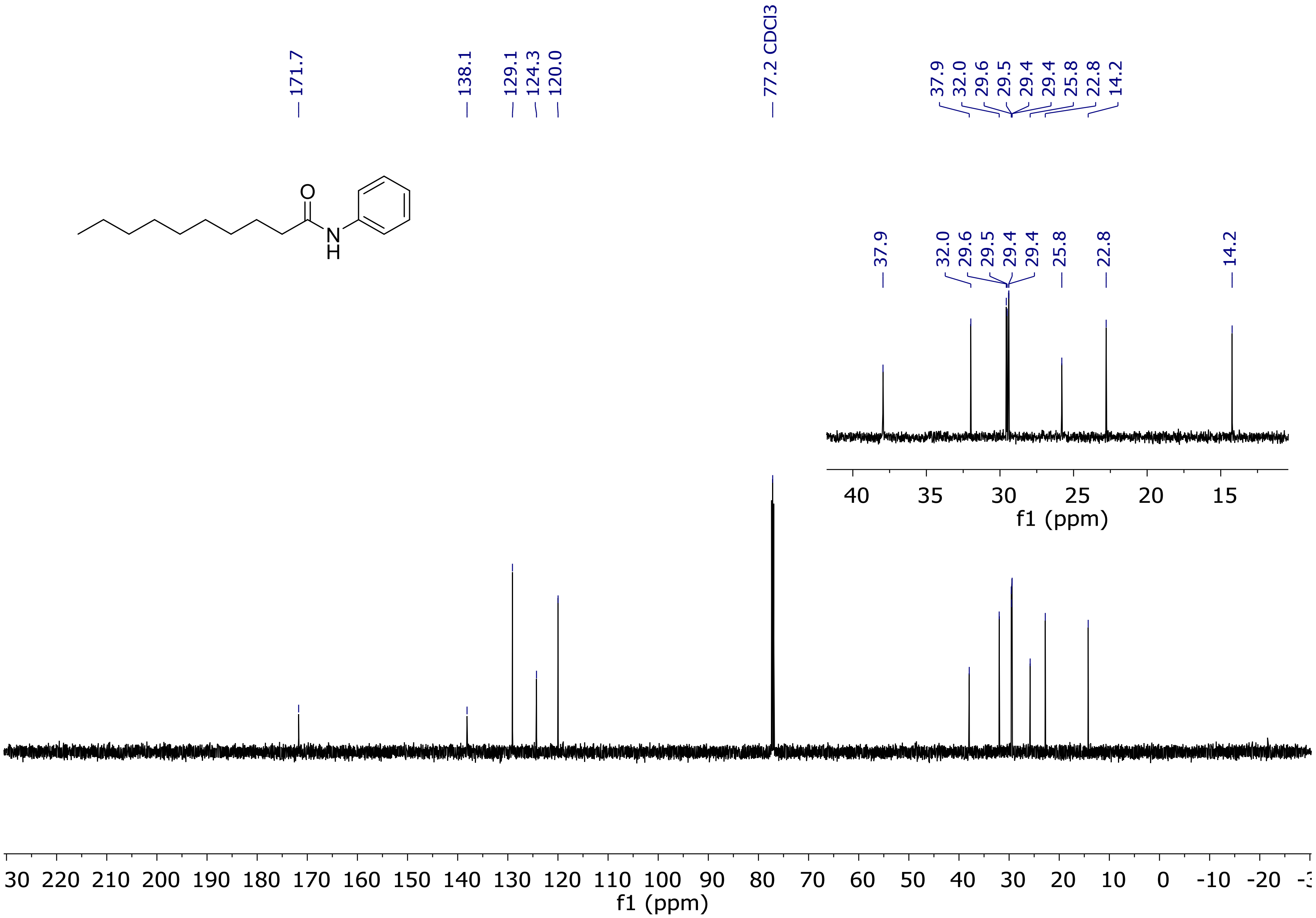

Figure S62. $^{13}C\{^1H\}$ **NMR (151 MHz, Chloroform-d) of N-phenyldecanamide (3df).**

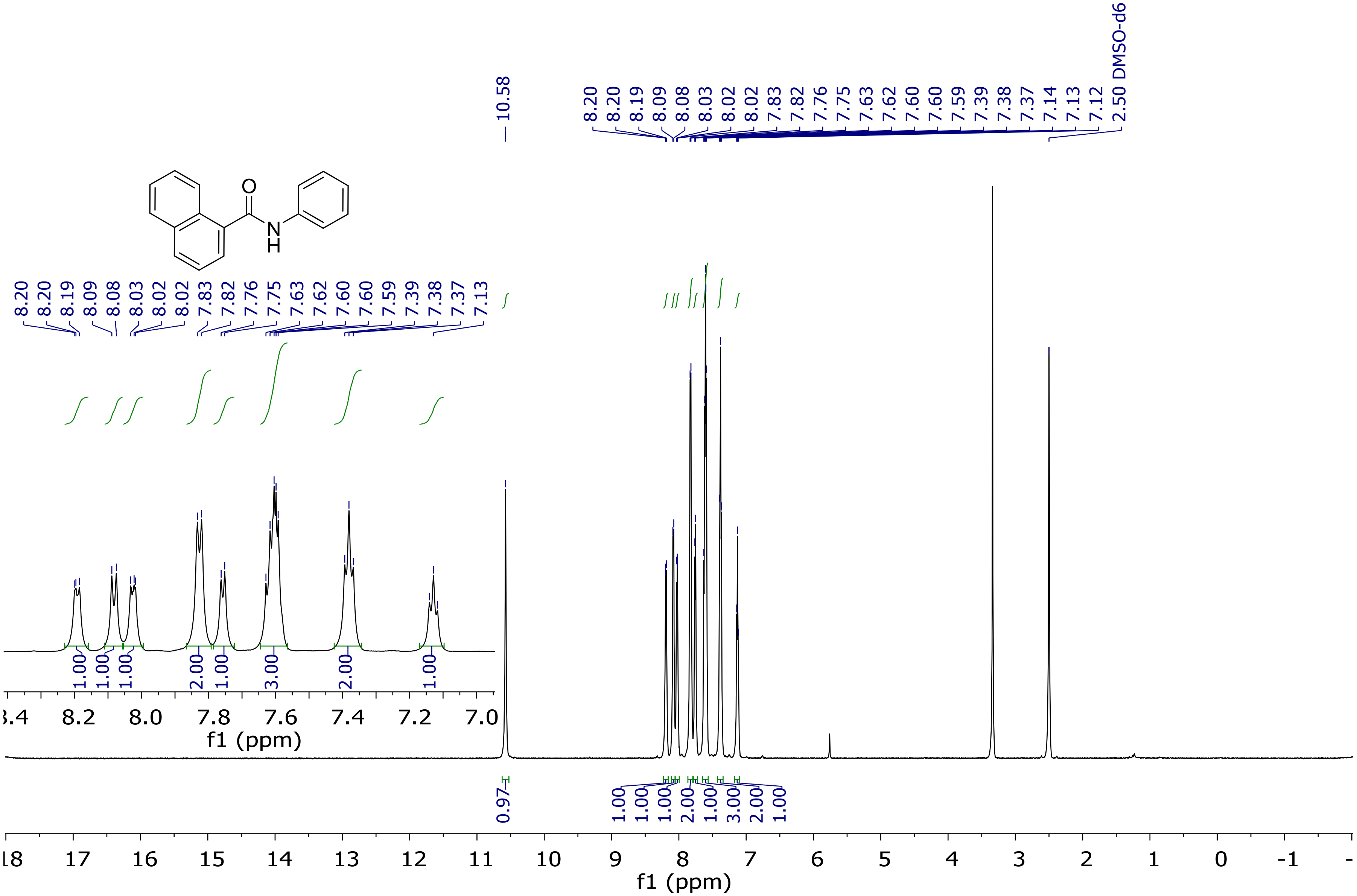

Figure S63. **^{1}H NMR (600 MHz, Chloroform-*d*) of N-phenyl-1-naphthamide (3dg).**

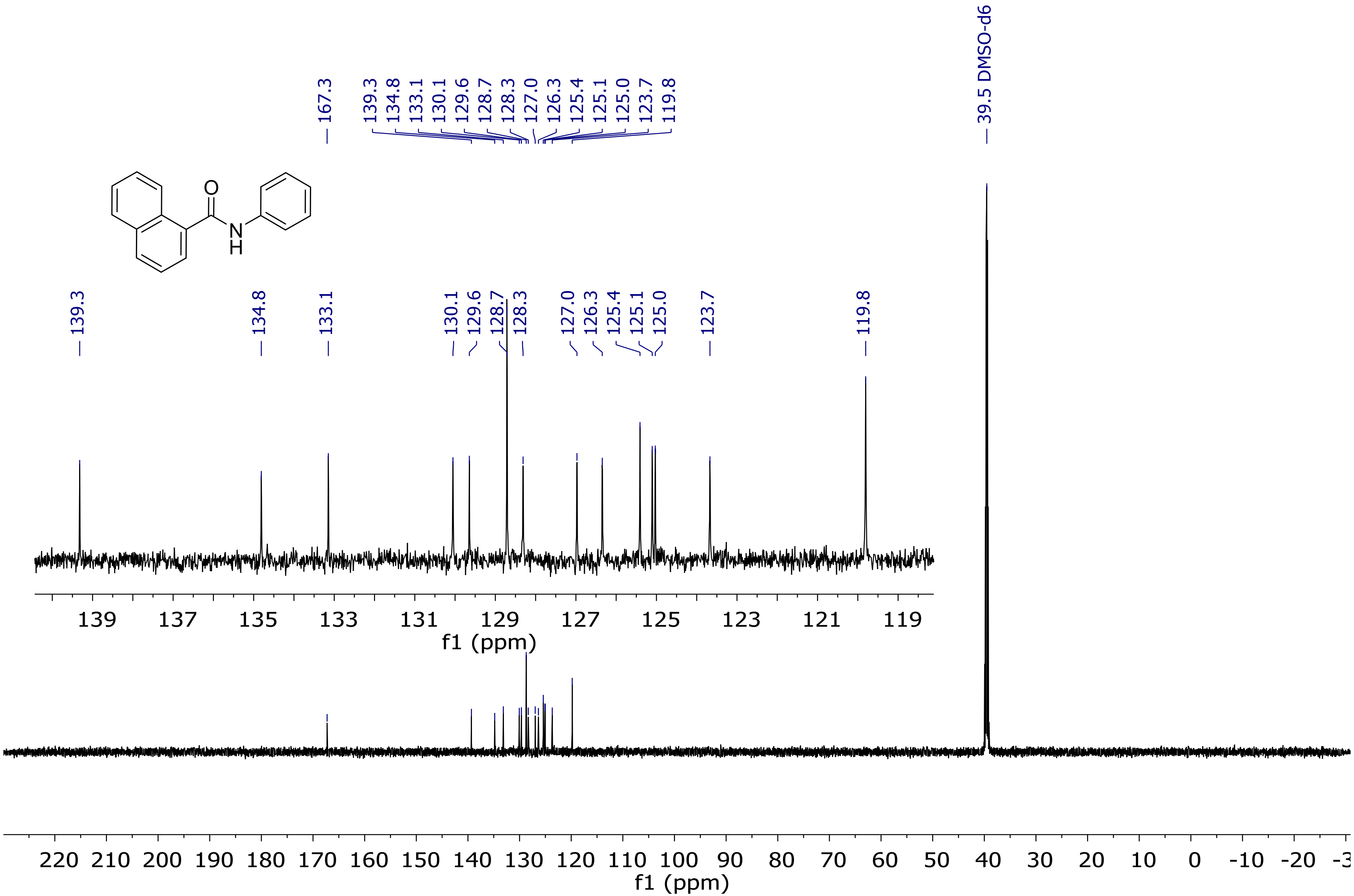

Figure S64. $^{13}C\{^1H\}$ **NMR (151 MHz, Chloroform-d) of N-phenyl-1-naphthamide (3dg).**

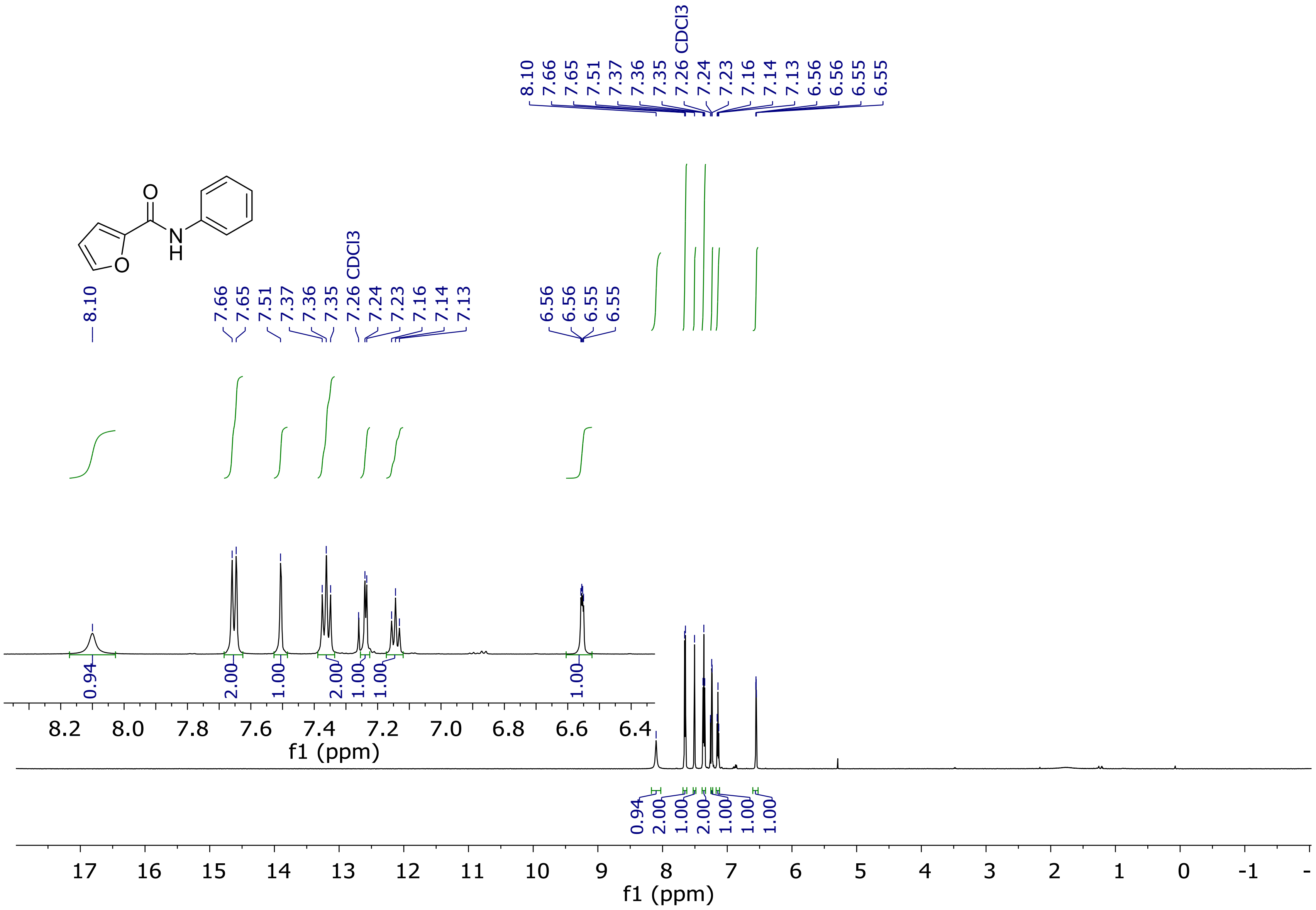

Figure S65. ¹H NMR (600 MHz, Chloroform-*d*) of N-phenylfuran-2-carboxamide (3dh).

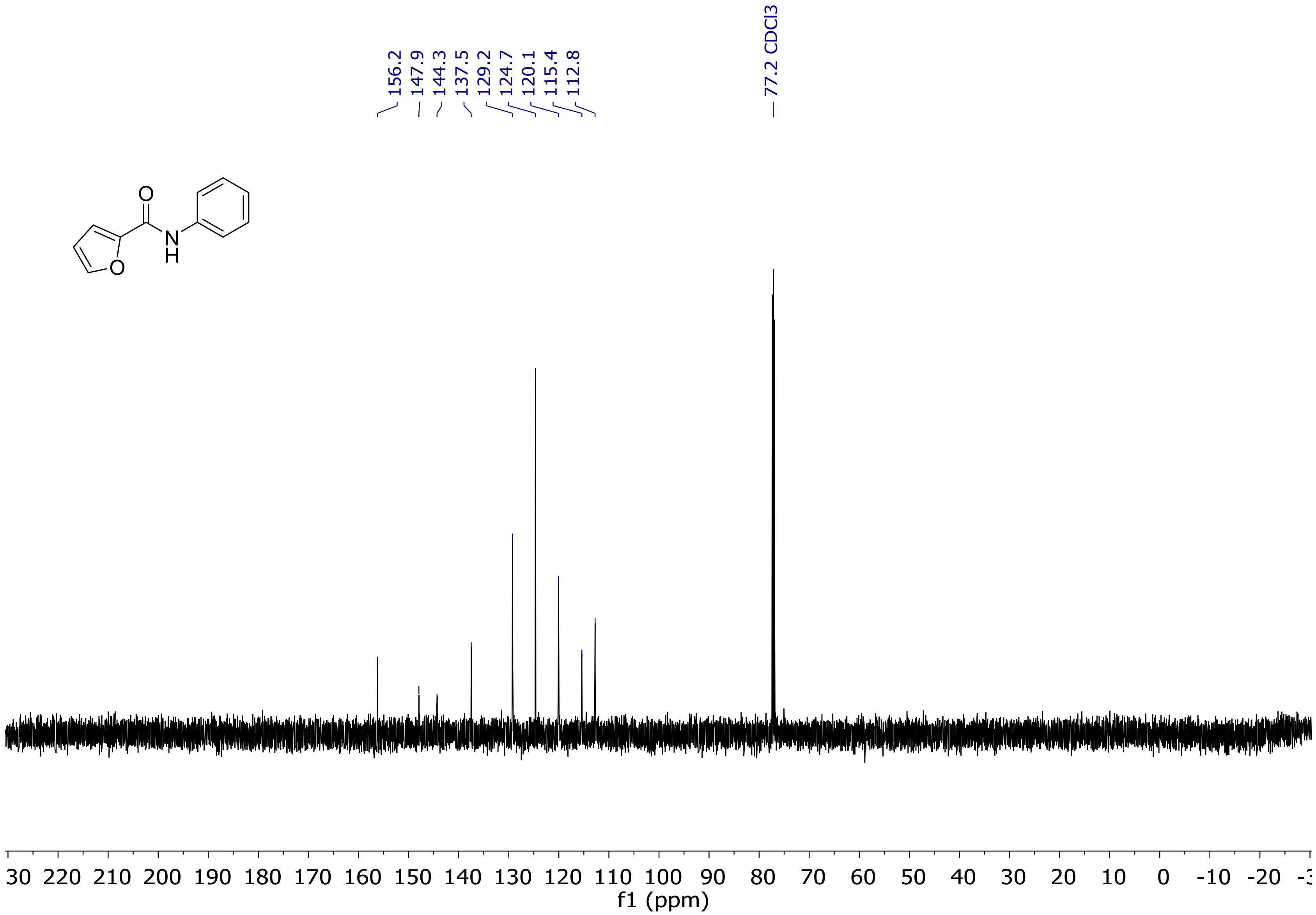

Figure S66. $^{13}C\{^{1}H\}$ **NMR (151 MHz, Chloroform-d) of N-phenylfuran-2-carboxamide (3dh).**

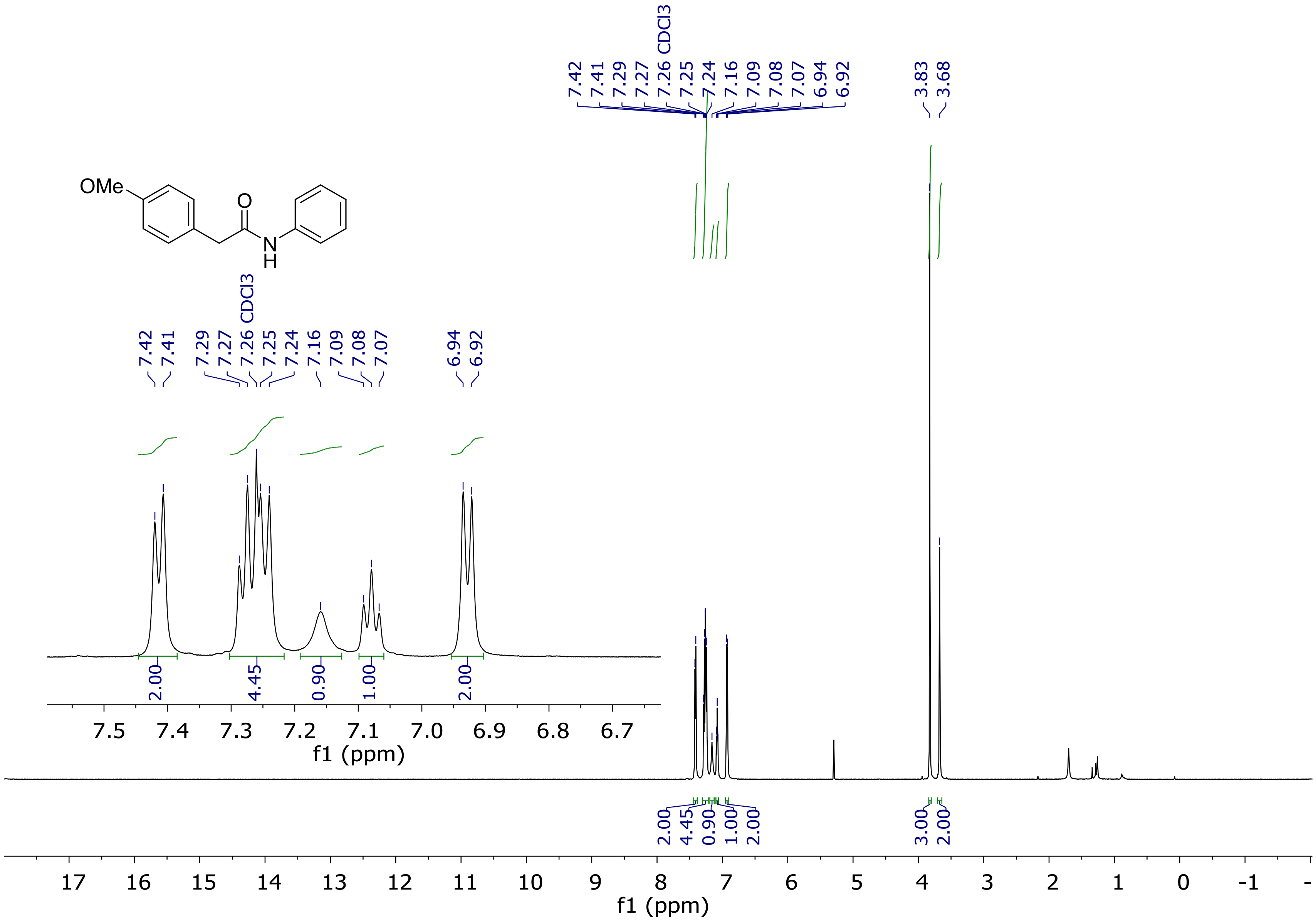

Figure S67. ^{1}**H NMR (600 MHz, Chloroform-*d*) of 2-(4-methoxyphenyl)-N-phenylacetamide (3di).**

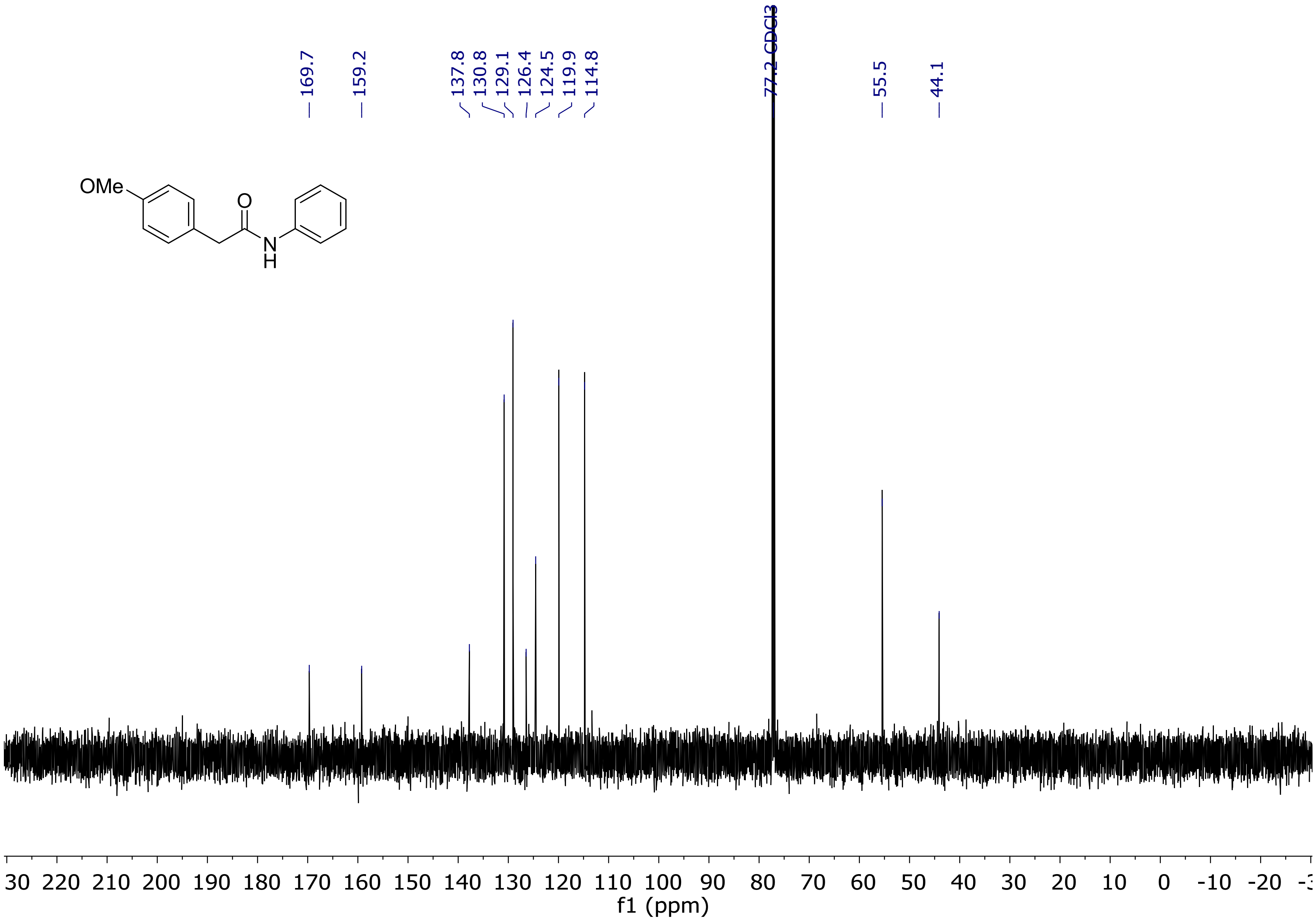

Figure S68. $^{13}C\{^{1}H\}$ **NMR (151 MHz, Chloroform-d) of 2-(4-methoxyphenyl)-N-phenylacetamide (3di).**

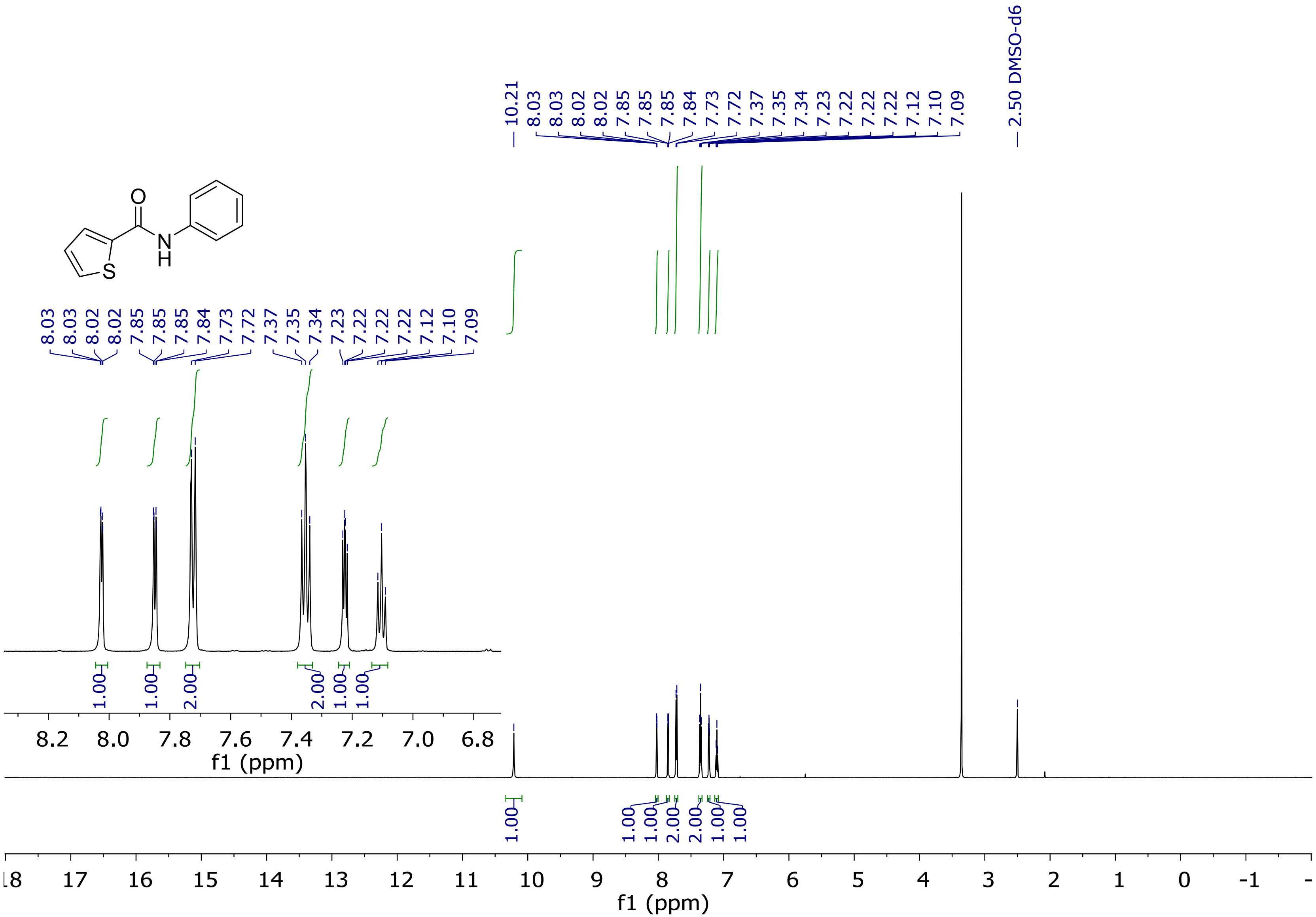

Figure S69. ^{1}H NMR (600 MHz, DMDO-*d6*) of N-phenylthiophene-2-carboxamide (3dj).

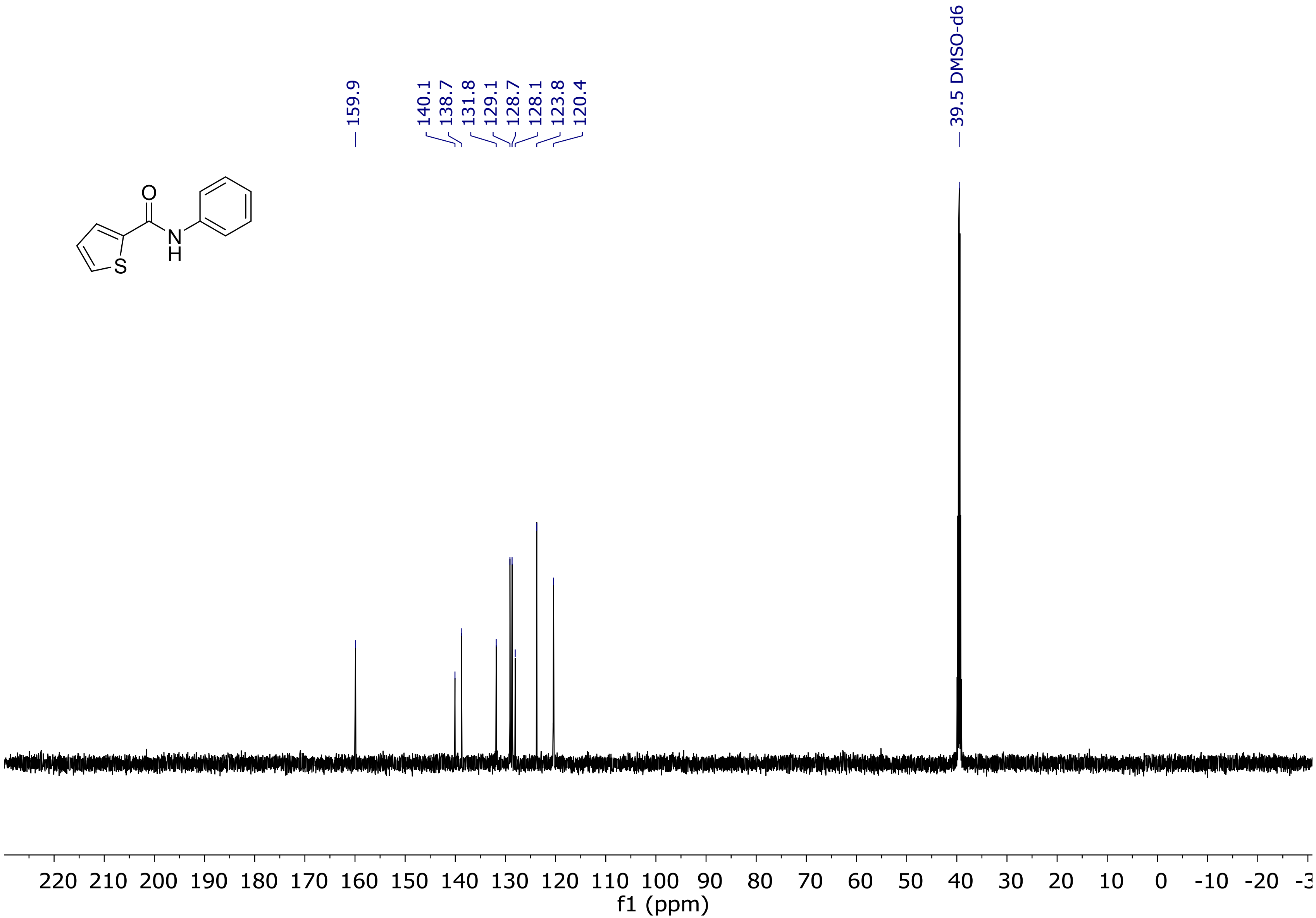

Figure S70. $^{13}C\{^{1}H\}$ **NMR (151 MHz, DMSO-*d6*) of N-phenylthiophene-2-carboxamide (3dj).**
